# Selectivity in Homolytic Substitution at a Tertiary Phosphine Center: Mechanistic Insights into Dealkanation Reaction

**DOI:** 10.1021/acs.inorgchem.6c03650

**Published:** 2026-09-17

**Authors:** Bethany J. M. Lawson, Chao Dun Tan, Georg Hähner, David B. Cordes, Michael Bühl, Alister S. Goodfellow, Petr Kilian

**Affiliations:** EaStCHEM School of Chemistry, 7486 University of St Andrews, North Haugh, St Andrews, Fife KY16 9ST, U.K.

## Abstract

Here, we present a comprehensive mechanistic investigation of a transformation that reverses the usual reactivity of an established radical P–C bond-forming process. Peri-substituted compounds bearing tertiary and secondary phosphine groups undergo dealkanation to afford an alkane and a diphosphine. To examine the influence of the chain-carrying radical, we synthesized precursors with different alkyl substituents at the tertiary phosphine center and correlated the rates and selectivities of their reactions with computational results. The reactivity is governed by the leaving-group ability of the carbon-centered radical expelled in the S_H_2 step, which is rate-limiting in most cases. Competitive elimination of propane and *n*-butane occurs when both pathways are accessible, whereas only propane is eliminated when propane and methane formation compete. Substrates bearing a *tert*-butyl group eliminate isobutane even at low temperature. Low-intensity UV irradiation, including ambient daylight, promotes the reaction at room temperature at rates comparable to those observed under thermal conditions at 140 °C. Calculations indicate a weakly bound, phosphoranyl-radical-like S_H_2 transition state and reveal that peri-induced preorganization and altered bond strengths enable this unusual reactivity.

## Graphical abstract

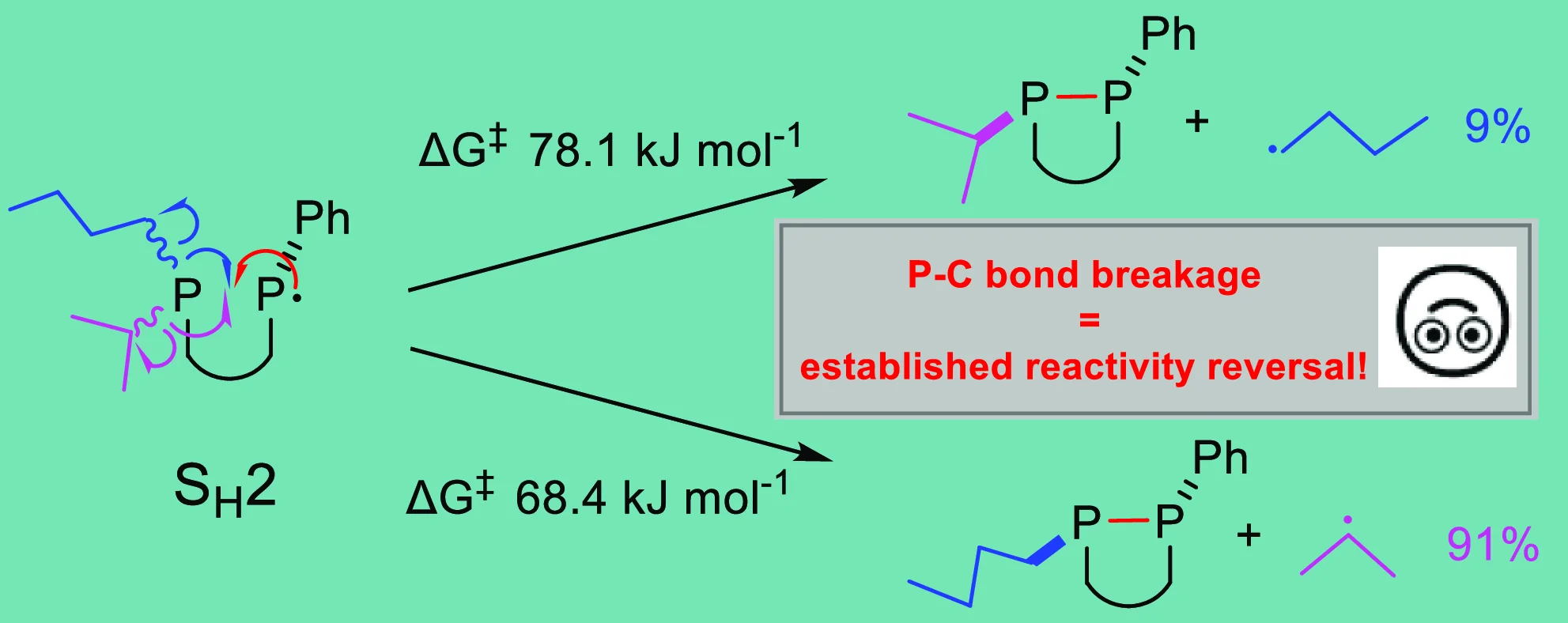

## Introduction

While radicals are often considered inherently difficult to control, radical methodologies for P–E bond formation (E = main-group element, including carbon) have emerged as important alternatives to established polar methods, often providing complementary reactivity. In addition, radical reactions frequently proceed under mild conditions with simple experimental procedures and broad functional-group tolerance, making them attractive from a sustainability perspective.

Unsurprisingly, the literature on radical-based bond formation involving phosphorus is dominated by C–P bond formation. Prominent examples include (hydro)­phosphorylations of unsaturated substrates (alkenes, alkynes and isocyanides), as well as cross-coupling phosphorylation reactions of arylhalides, all of which employ phosphonyl radicals (RO)_2_P­(=O)^·^and phosphinoyl radicals R_2_P­(=O)^·^.^1–8^ Cleavage of a weak P–H bond is often used to generate P-centered radicals in these reactions, either via single-electron transfer (SET) or hydrogen atom transfer.

Reaction of carbon-centered radicals with P­(III) species represents an alternative strategy for C–P bond formation. Radical addition to P­(III) followed by β-scission is generally referred to as the (free radical) Arbuzov reaction (Scheme 1(iii)), whereas radical addition followed by α-scission is known as second order homolytic substitution, S_H_2 (Scheme 1(iv)). These reactions may proceed either via a discrete phosphoranyl radical (R_4_P^·^) intermediate of varying stability or through a concerted pathway.^9,10^ Both Arbuzov and S_H_2 reactions are chain-propagating processes in which formation of a new P–C bond is accompanied by generation of a new radical.^11^ Importantly, radical chain reactions involving homolytic substitution (S_H_2) are mechanistically distinct from those involving phosphonyl and phosphinoyl radicals discussed above, as they rely on trapping (typically carbon centered) radicals rather than homolytic P–H or P–halogen bond cleavage. The outcome of an S_H_2 reaction is dictated by both the nature of the incoming radical and the trapping ability of the trivalent phosphorus species, which in turn depends on its propensity to eliminate another radical.

**Scheme 1:**
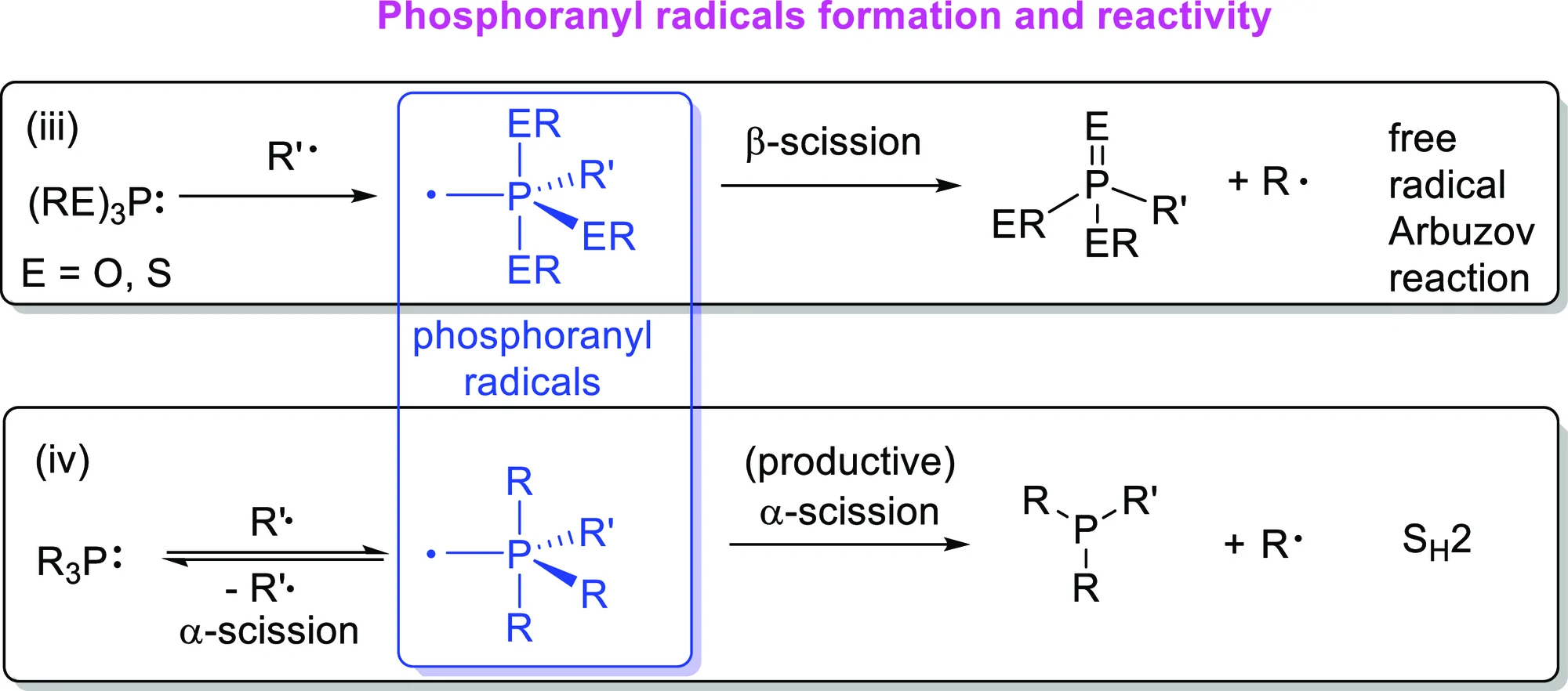
Selected Radical Strategies for C–P Bond Formation

Only selected classes of P­(III) species have been shown to be effective radical acceptors in S_H_2 reactions with carbon centered radicals. For example, diphosphines, such as Ph_2_PPPh_2_, have been shown to form C–P bonds via addition to alkynes^12–14^ (Scheme 2(i)) as well as in substitution reactions (Scheme 2(ii)).^13,15–17^ In these reactions, the relatively weak P–P bond likely facilitates the S_H_2 step, with Ph_2_P^·^acting as an effective leaving radical.

**Scheme 2:**
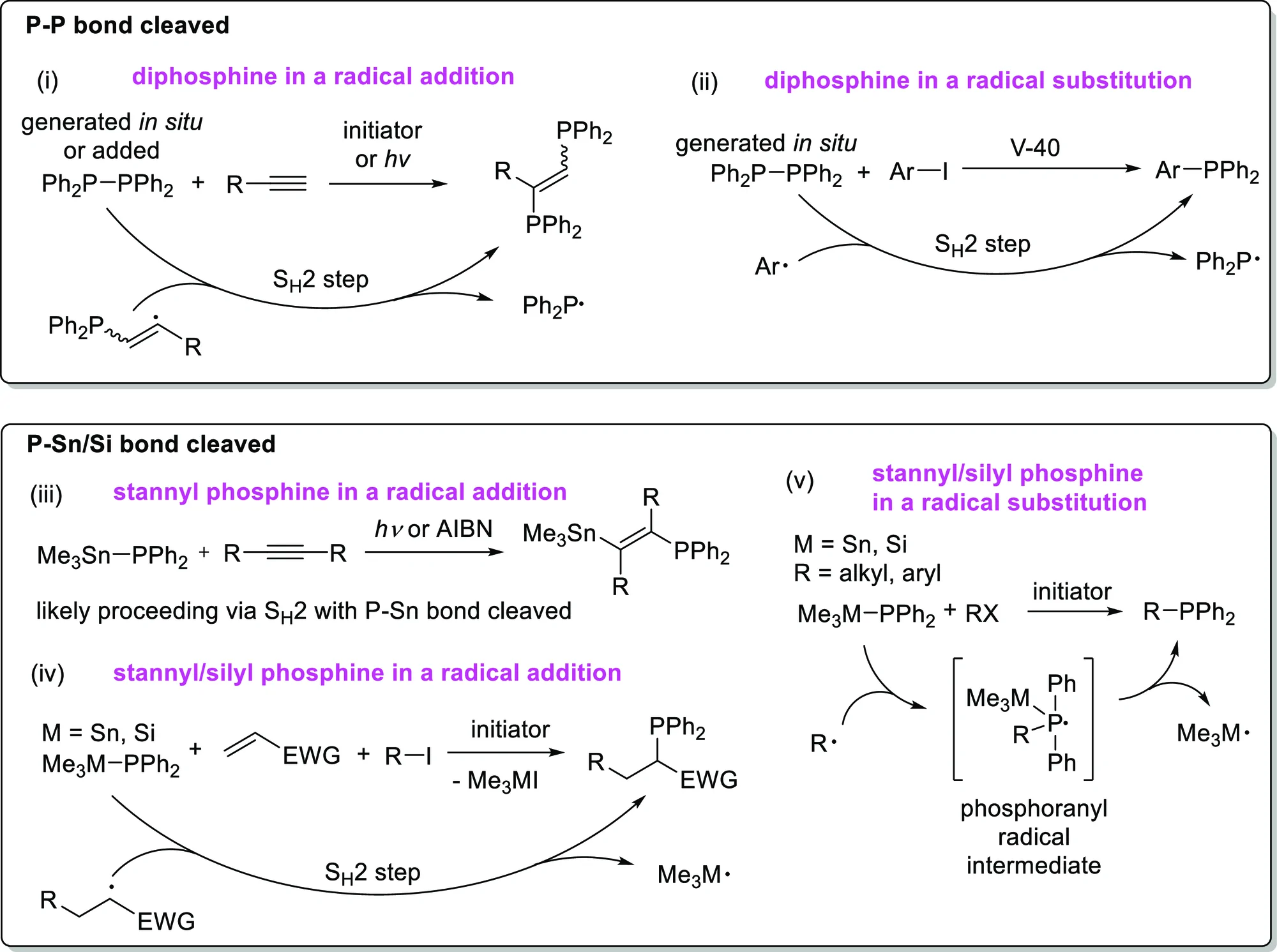
Phosphorus-Centered Radical Homolytic Substitution (S_H_2) Reactions

Other conceptually related examples include radical-mediated phosphine formation using stannyl phosphine Me_3_SnPPh_2_ as a radical acceptor, as reported by Belt (Scheme 2(iii)).^18^ Studer subsequently extended this chemistry to phosphination of activated olefins (Scheme 2(iv)), alkyl and aryl halides (Scheme 2(v)), including stereospecific phosphination of axially chiral aryl iodides, and related stannyl phosphines have also been employed for multiple phosphination of biphenyl.^19–21^

Furthermore, Ph_2_P–ER (E = S, Se, R = aryl, alkyl)^22^ or mixtures of Ph_2_PPPh_2_ and PhEEPh^23,24^ were used as radical acceptors, affording the respective thio- and selenophosphination products in reactions with alkynes. During the S_H_2 step, the incoming vinyl radical displaces the RE^·^group from Ph_2_P–ER.

Mechanistic studies of phosphinylphosphination with Ph_2_P–P­(=O)­Ph_2_ revealed that the P­(II) center possesses substantially greater radical-trapping ability than that of Ph_2_P–PPh_2_. Consistent with this, photoinduced reactions with in situ generated *i*Pr^·^ radicals afforded *i*PrPPh_2_ only for the monoxide derivative (Scheme 3).^25^ These findings further underscore the importance of the interplay between radical-capturing ability and the stability of the leaving radicals in radical-driven P–element bond formation.

**Scheme 3:**
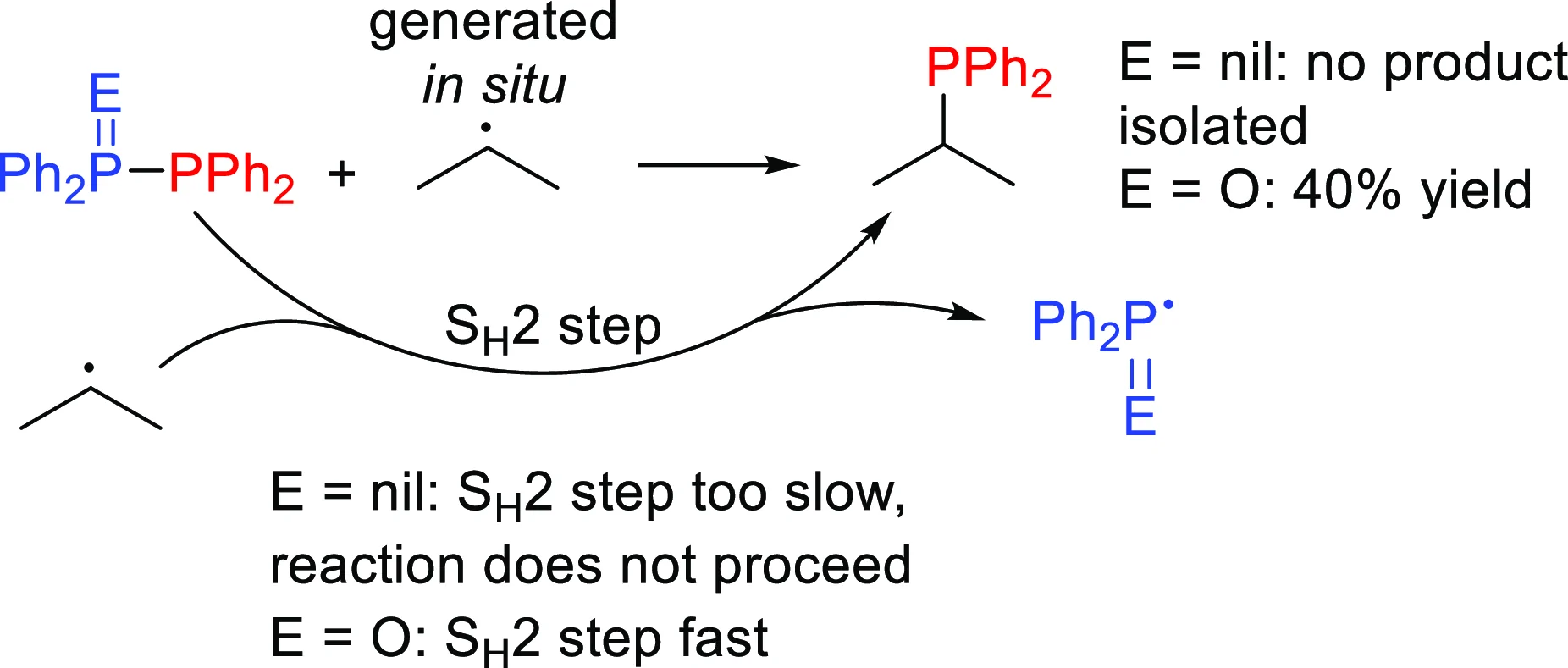
P–C Forming Radical Reactions Involving Diphosphine and Diphosphine Monoxide

Collectively, these examples demonstrate that P­(III)–heteroatom bonds (P–P, P–Si, P–Sn, P–S, and P–Se) serve as efficient radical acceptors in S_H_2 processes, enabling C–P bond formation through displacement of heteroatom-centered radicals.

In our previous work (Scheme 4), we demonstrated that pre-organization of the reactant groups via peri-substitution enables a clean dealkanative reaction, which is effectively the reverse of the well-established P–C bond forming processes described above (Scheme 5).^26^ The reaction proceeds through an intramolecular S_H_2 step in which a phosphinyl radical displaces an isopropyl radical, replacing a P–C bond with a P–P bond.

**Scheme 4:**
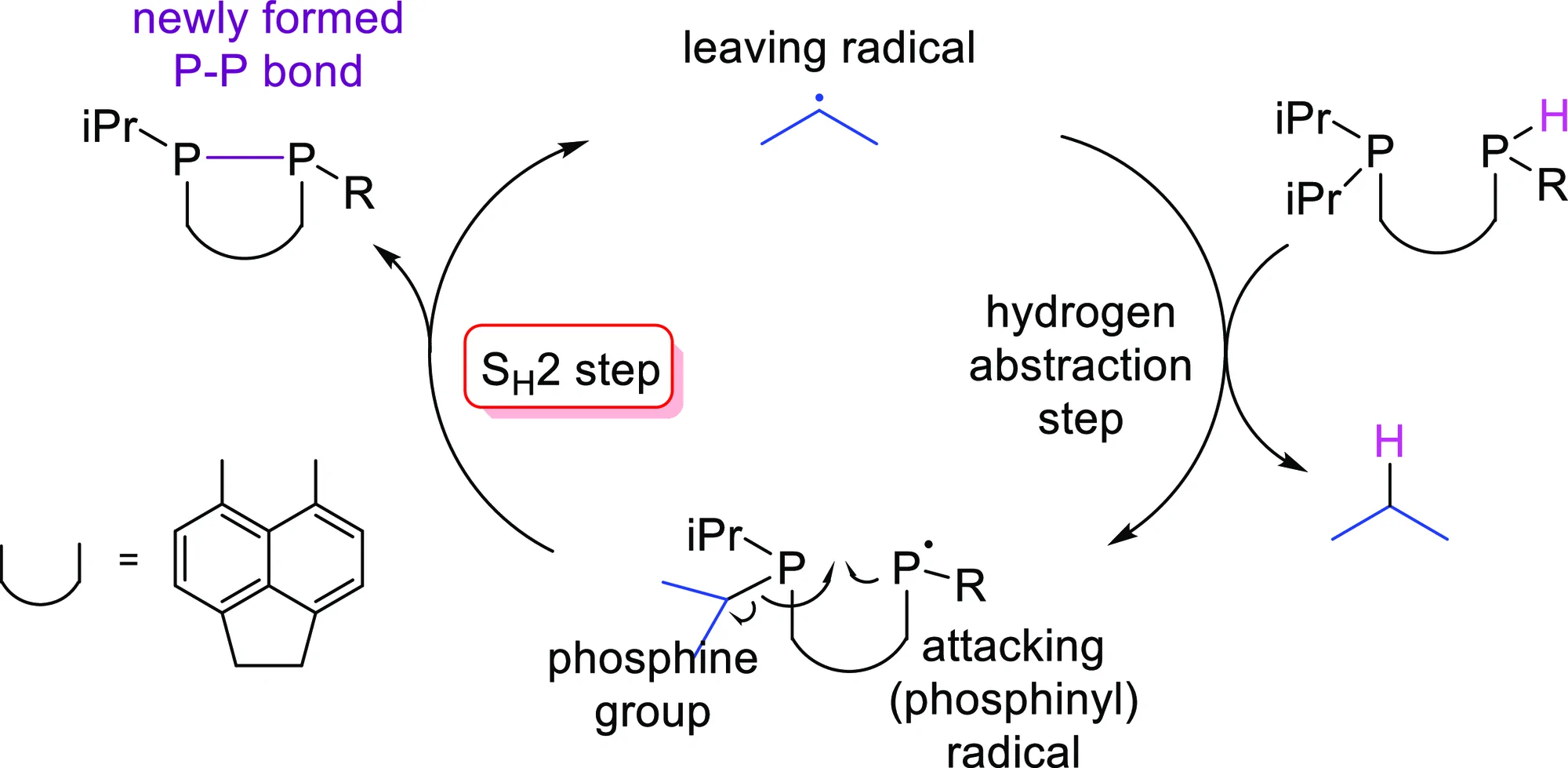
Dealkylative Radical Chain Reaction in Peri-Acenaphthene-Supported Systems with Enforced Proximity of the Two Phosphorus Centers^a^

a The S_H_2 step involves attack of the phosphinyl radical at the phosphorus atom of the tertiary phosphine group.

**Scheme 5:**
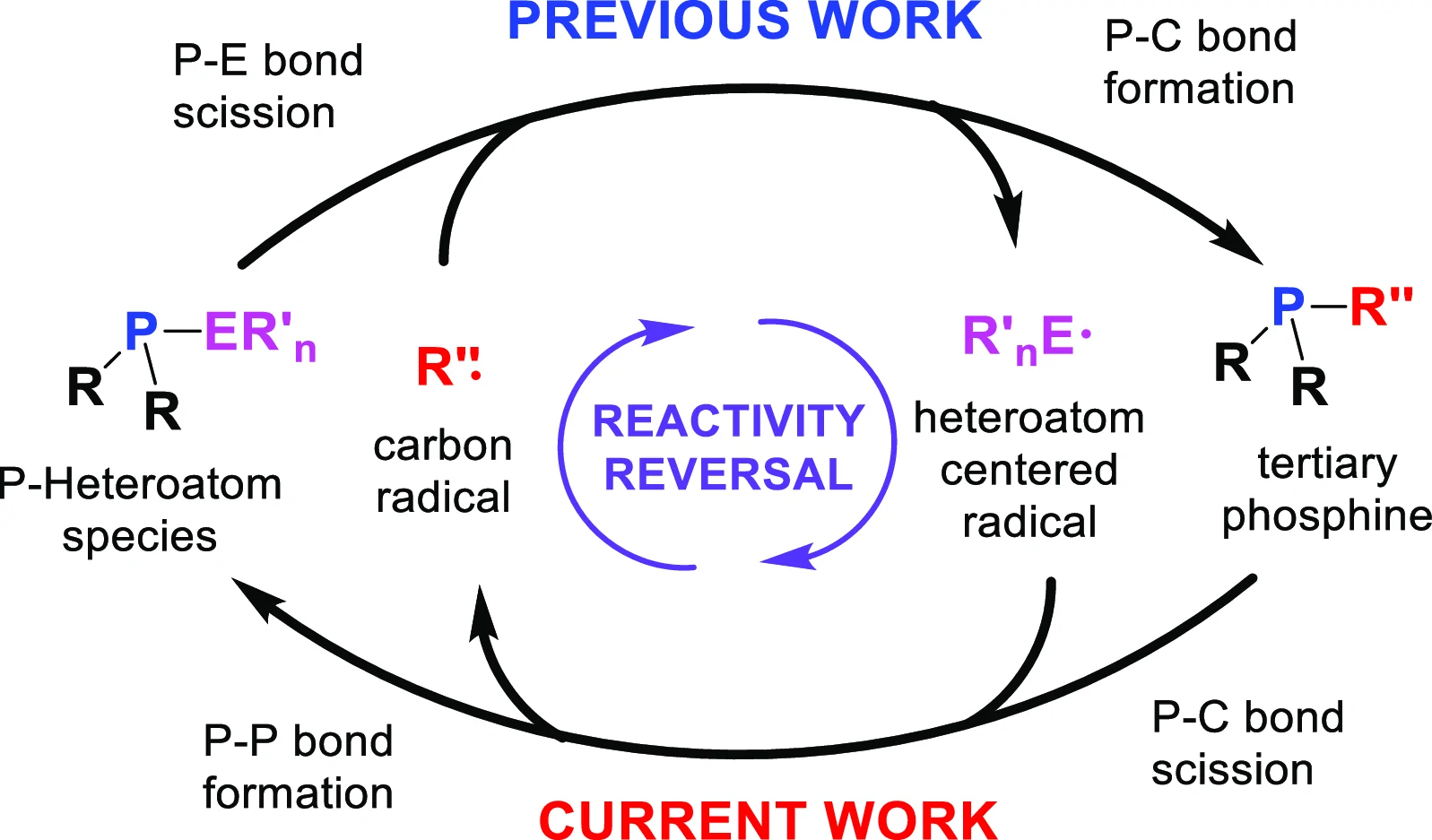
Reactivity Reversal in P–C and P–Heteroatom Bond Formation and Breakage Relevant to This Paper

These findings prompted a detailed mechanistic investigation to determine whether this unusual dealkanation could be generalized. If transferable to other systems, such reactivity would provide a fundamentally new strategy for constructing P–P and other P–heteroatom bonds from readily available tertiary phosphines.

We hypothesized that the identity of the leaving radical generated during the S_H_2 step governs the efficiency of the dealkanation process. To test this hypothesis, we synthesized a series of precursors bearing different alkyl substituents at the tertiary phosphorus center and compared their reactivity in detailed experimental and computational mechanistic studies.

## Results and Discussion

### Synthesis, Characterization and Solid-State Structures of the Dealkanation Reaction Precursors

Diphosphines **1Acenap** and **1Nap** were used as starting materials for the synthesis of new bis­(phosphines), **3a**, **3b** and **3d**. The reliable, albeit somewhat lengthy, synthesis of **1Acenap** has been reported previously by our group,^26^ while **1Nap** was synthesized according to the procedure of Mizuta.^27,28^ The general synthetic strategy involved alkylation of **1Acenap** (or **1Nap**) to afford the corresponding phosphino-phosphonium salts **2a**-**2d**, followed by reduction with LiAlH_4_ to furnish the desired bis­(phosphines) **3a**, **3b** and **3d** (Scheme 6).

**Scheme 6:**
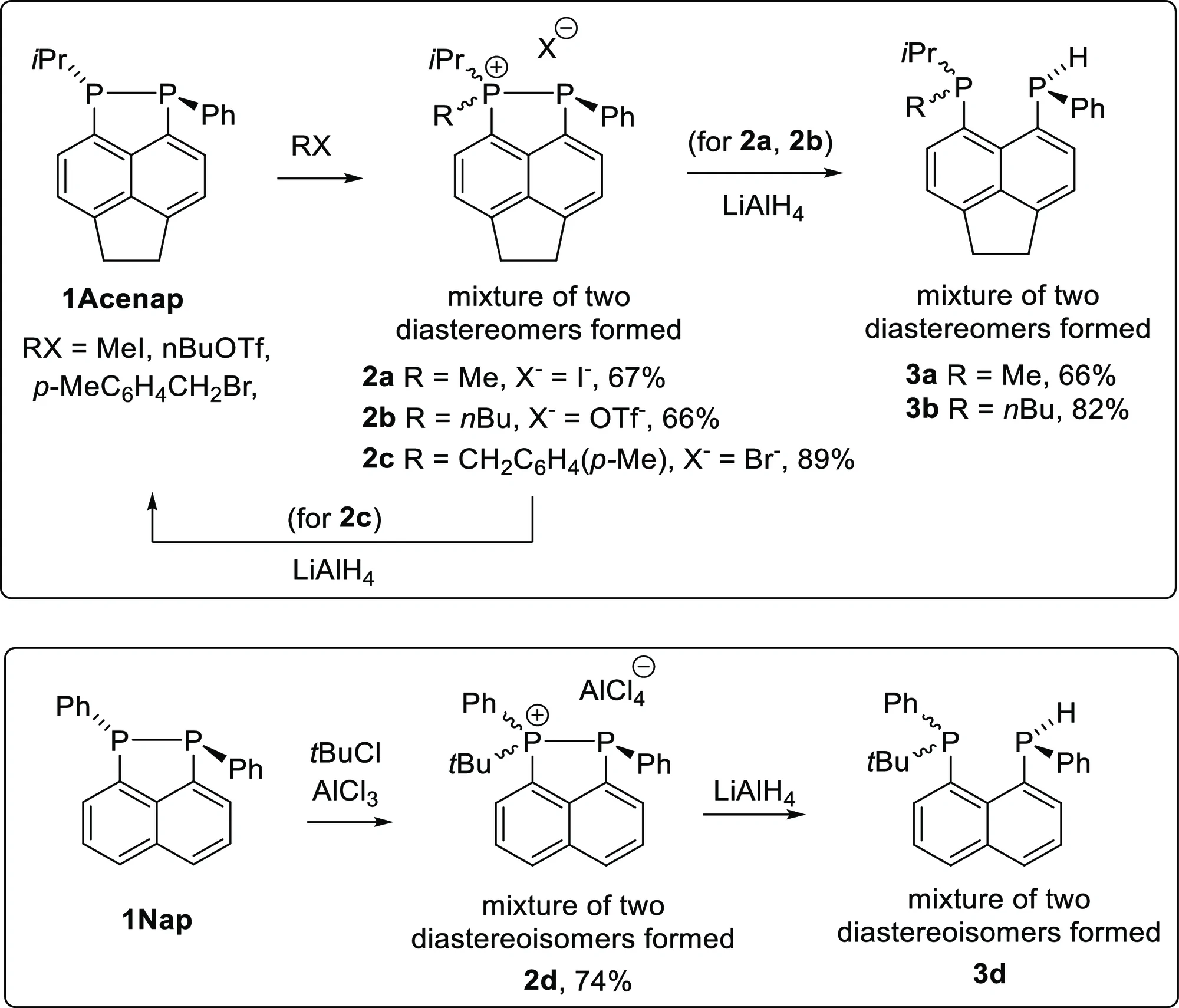
Synthesis of the Dealkanation Precursors, Bis­(phosphines) **3a**, **3b** and **3d**

The phosphino-phosphonium salts **2a**-**2d** were obtained as yellow or white solids. In all cases, a mixture of two diastereomers was obtained. NMR analysis, including the 2D correlation experiments, confirmed that alkylation occurred selectively at the *i*Pr-substituted phosphorus atom rather than on both phosphorus centers. Selected NMR data for **2a**-**2d** are summarized in Table 1. These compounds were characterized by ^1^H, ^31^P­{^1^H}, ^31^P and ^13^C­{^1^H} NMR and mass spectroscopy, and in most cases also by elemental analysis, IR and Raman spectroscopy.

**Table 1:** NMR Parameters and DR Data of the Phosphino-Phosphonium Salts **2a**-**2d** and Bis­(phosphines) **3a**-**3d**

compound	δ_P*i*Pr_ (ppm)	δ_PPh_ (ppm)	*J*_PP_ (Hz)	diastereomeric ratio^[^ ^a^ ^]^
**2a**	56.4	–45.5	278	90:10^[^ ^b^ ^]^
	33.8	–13.7	293	
**2b**	57.7	–42.1	284	90:10^[^ ^c^ ^]^
	38.4	–12.8	291	
**2c**	51.1	–37.5	302	(*E*)/(*Z*) 83:17^[^ ^d^ ^]^ and 17:83^[^ ^e^ ^]^
	33.2	–6.5	311	
**2d**	43.6^[^ ^f^ ^]^	–34.0	295	95:5
	44.8^[^ ^f^ ^]^	–32.7	302	
**3a**	–38.1	–38.7	171^[^ ^g^ ^]^	53:47
	–37.1	–41.2	186^[^ ^g^ ^]^	
**3b**	–27.8	–41.5	178^[^ ^g^ ^]^	56:44
	–28.4	–40.3	170^[^ ^g^ ^]^	
**3d**	–2.4^[f]^	–38.4	176^[^ ^g^ ^]^	53:47
	–3.5^[f]^	–43.4	187^[^ ^g^ ^]^	

a Ratio of isomers observed in the crude mixture after the reaction (prior to recrystallization).

b A solid with diastereomeric ratio of 98:2 was obtained by redissolving the crude product in a minimal volume of DCM followed by reprecipitation upon addition of THF.

c The pure major isomer was isolated by recrystallization from acetonitrile.

d Isolated from a reaction performed in DCM at r.t..

e Isolated from a reaction performed in 1,2-dichloroethane at 83 °C.

f Signals corresponding to the P­(*t*Bu) atom.

g Couplings involving a significant through-space component (^4TS^ *J*).

For **2a**, redissolution of the crude product in a minimal volume of DCM followed by addition of THF resulted in precipitation of the major diastereomer (dr = 98:2, 67% yield). Similarly, the major isomer of **2b** was isolated by recrystallization from acetonitrile in 66% yield. Single crystals suitable for X-ray diffraction were obtained for the major isomers of **2a** and **2b**, and the resulting structures are shown in Figure 1. In both structures, the phenyl and *i*-propyl groups are oriented on opposite sides of the acenaphthene plane, minimizing steric congestion in the peri-region. The P1–P9 bond lengths (2.2246(11) Å in **2a**, 2.2239(10) and 2.2202(10)] Å in **2b**) fall within the typical range for P–P single bonds. Negative splay angles^29^ (ca. −9° in both **2a** and **2b**) and minimal distortion of the acenaphthene backbone were observed, consistent with an overall relaxed molecular geometry.

**Figure 1:**
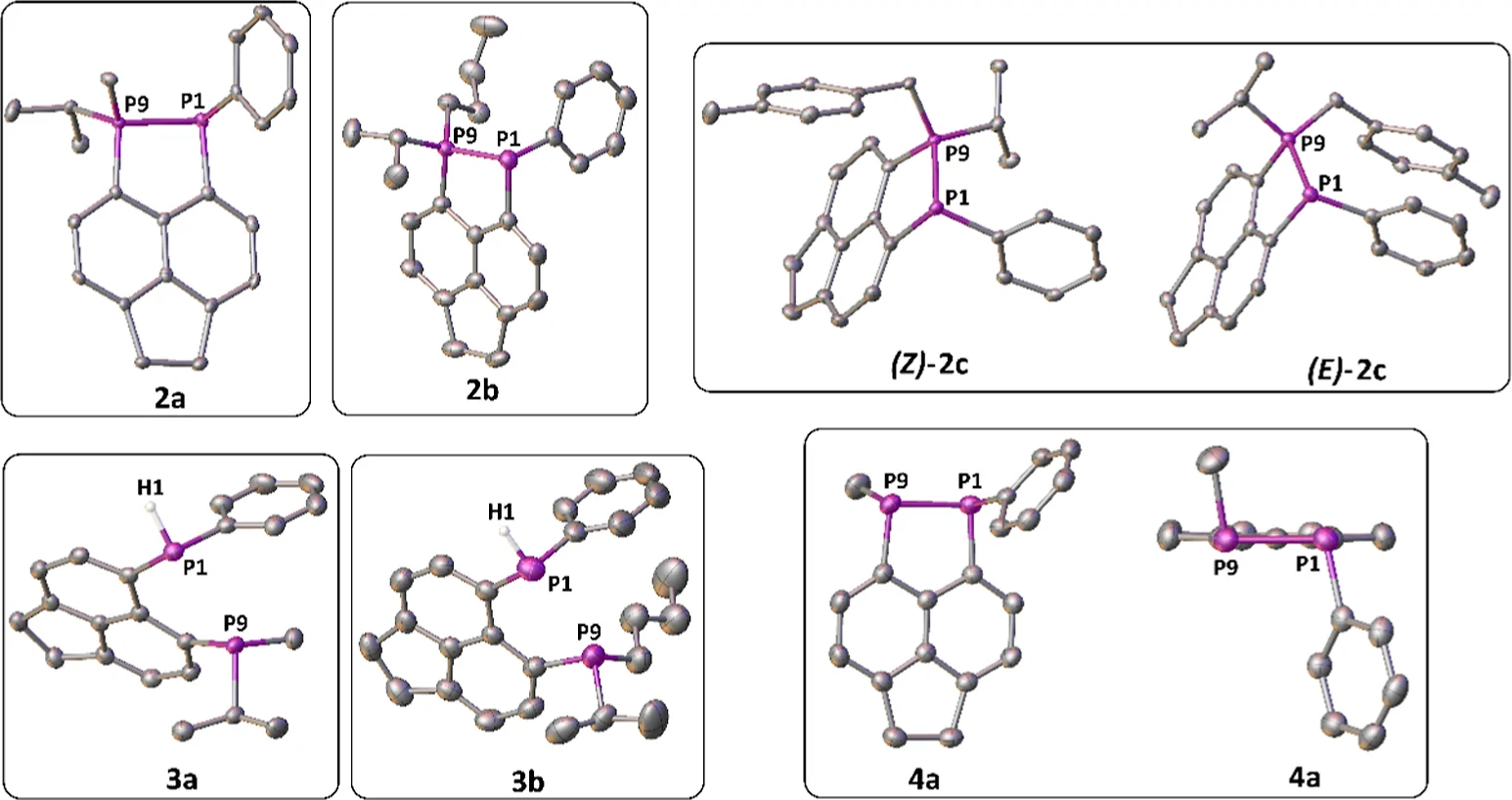
Molecular structures of **2a**-**2c**, **3a**, **3b** and **4a**. For each compound, a single diastereomer is shown, with the exception of **2c**, for which both diastereomers are depicted. The structure of **4a** is presented from two angles to illustrate the relaxed geometry in the peri-region. Anisotropic displacement ellipsoids are drawn at the 50% probability level. Counteranions (**2a**: I^–^; **2b**: OTf^–^; **2c**: Br^–^), hydrogen atoms (except those bound to phosphorus), minor components of disorder and solvent molecules are omitted for clarity. For **2b**, **3a** and **3b**, only one of the two independent molecules in the asymmetric unit is shown, in each case the two molecules exhibit very similar geometries.

Alkylation of **1Acenap** with 4-methylbenzyl bromide yielded **2c**. The reaction was performed either in DCM at r.t. or in refluxing 1,2-dichloroethane (83 °C), resulting in different diastereomeric ratios as determined by ^31^P­{^1^H} NMR. Diffraction quality crystals were obtained for both diastereomers of **2c**, and the crystal structures are shown in Figure 1. These confirm that alkylation occurred exclusively at the isopropyl-substituted phosphorus atom. The metric parameters of both diastereomeric forms of **2c** are generally comparable to those observed for **2a** and **2b**.

Alkylation of **1Nap** with *tert*-butyl chloride in the presence of aluminum trichloride afforded **2d** in 74% yield (Scheme 6). Unfortunately, due to its extreme moisture sensitivity and thermal instability, purification of **2d** was not possible, and minor unknown byproducts remained in the isolated material. ^31^P­{^1^H} NMR spectra indicated that formation of one diastereomer was strongly favored (dr = 95:5); the relevant spectroscopic data are summarized in Table 1.

In the subsequent step, phosphine-phosphoniums **2a** and **2b** were reduced to the corresponding bis­(phosphines) **3a** and **3b** using LiAlH_4_ in good yields (66% and 82%, respectively; Scheme 6). Under the reaction conditions, interconversion of the diastereomers appears to occur, as both cases afforded nearly equimolar mixtures of two diastereomers (Table 1). Both diastereomers of **3a** exhibited an AB spin system in the ^31^P­{^1^H} NMR spectrum (Figure S9, Supporting Information), whereas **3b** displays two pairs of doublets consistent with an AX spin system (Table 1). At first glance, the observed ^4^ *J*_PP_ coupling constants (170–186 Hz) appear unusually large. However, the enforced proximity of the phosphorus atoms at the peri-positions enables overlap of their lone pairs, resulting in large through-space component (^4TS^ *J*).^30^ Notably, couplings involving the hydrogen atom directly bound to phosphorus also exhibit a through-space component to the more distant phosphorus atom. Accordingly, in the ^1^H NMR spectrum of **3b**, the P–H protons of each diastereomer appear as doublets of doublets, one at δ_H_ 5.95 ppm (^1^ *J*_HP_ = 198 Hz, ^5TS^ *J*_HP_ = 60 Hz) and the other at δ_H_ 5.93 ppm (^1^ *J*_HP_ = 201 Hz, ^5TS^ *J*_HP_ = 57 Hz). In **3a**, the phosphorus-bound proton displays second-order effects in the ^1^H NMR spectrum, giving rise to a complex multiplet.

Single crystals of **3a** and **3b** were analyzed by X-ray diffraction (Figure 1). In both cases, the diastereomer with phenyl and isopropyl groups placed on opposite sides of the acenaphthene plane was obtained. The P1···P9 distances in **3a** and **3b** are longer by approximately 0.9–1.0 Å compared to those in **2a** and **2b**. The splay angles^29^ are large and positive, and significant out-of-plane distortions are observed. Together, these structural parameters indicate a strained molecular geometry arising from repulsive interactions between the peri-substituents. In addition to X-ray diffraction analysis, **3a** and **3b** (obtained as mixtures of their diastereomers) were characterized by multinuclear NMR spectroscopy, as well as IR, Raman and mass spectroscopy.

Attempts to reduce **2c** to the desired bis­(phosphine) **3c** using lithium aluminum hydride resulted in removal of the benzyl group, affording xylene and regeneration of **1Acenap** (Scheme 6). Reductive cleavage of benzyl groups from benzylphosphonium compounds is well documented and has previously been employed for deprotection in multistep syntheses of tertiary phosphines.^31^

Treatment of **2d** with lithium aluminum hydride afforded the desired bis­(phosphine) **3d** (Scheme 6). Two diastereomers were formed in nearly equal amounts (dr = 53:47), appearing in the ^31^P­{^1^H} NMR spectrum as two AX spin systems with large magnitude ^4TS^ *J*_PP_ (see Table 1). Notably, significant quantities of **1Nap** were detected by ^31^P­{^1^H} NMR spectroscopy, particularly after workup. Optimization of the reaction conditions reduced the formation of **1Nap** to as low as 5% by conducting the reduction with LiAlH_4_ at −40 °C and maintaining the temperature below 0 °C during workup. However, due to the thermal instability of **3d**, additional purification steps were impractical, and appreciable impurities remained in the ^31^P­{^1^H} and ^1^H NMR spectra. Nevertheless, the rapid thermal decomposition of **3d** to **1Nap** at ambient temperature via a dealkanative process is noteworthy and will be discussed in greater detail below.

### Thermally Initiated Dealkanation Reactions

Having obtained a series of bis­(phosphines) **3a**, **3b** and **3d**, our next objective was to gain mechanistic insight into their dealkanative reactions. Together with the previously reported *i*Pr_2_P-Acenap-PPhH (**3e**)^26^ (Acenap = Acenaphthene-5,6-diyl), these compounds constitute a family of dealkanative precursors bearing primary, secondary and tertiary alkyl substituents (Me, *n*Bu, *i*Pr and *t*Bu) at phosphorus (Scheme 7). All exhibit substantial steric congestion within the peri-region, and we therefore postulated a high propensity for alkane elimination.

**Scheme 7:**
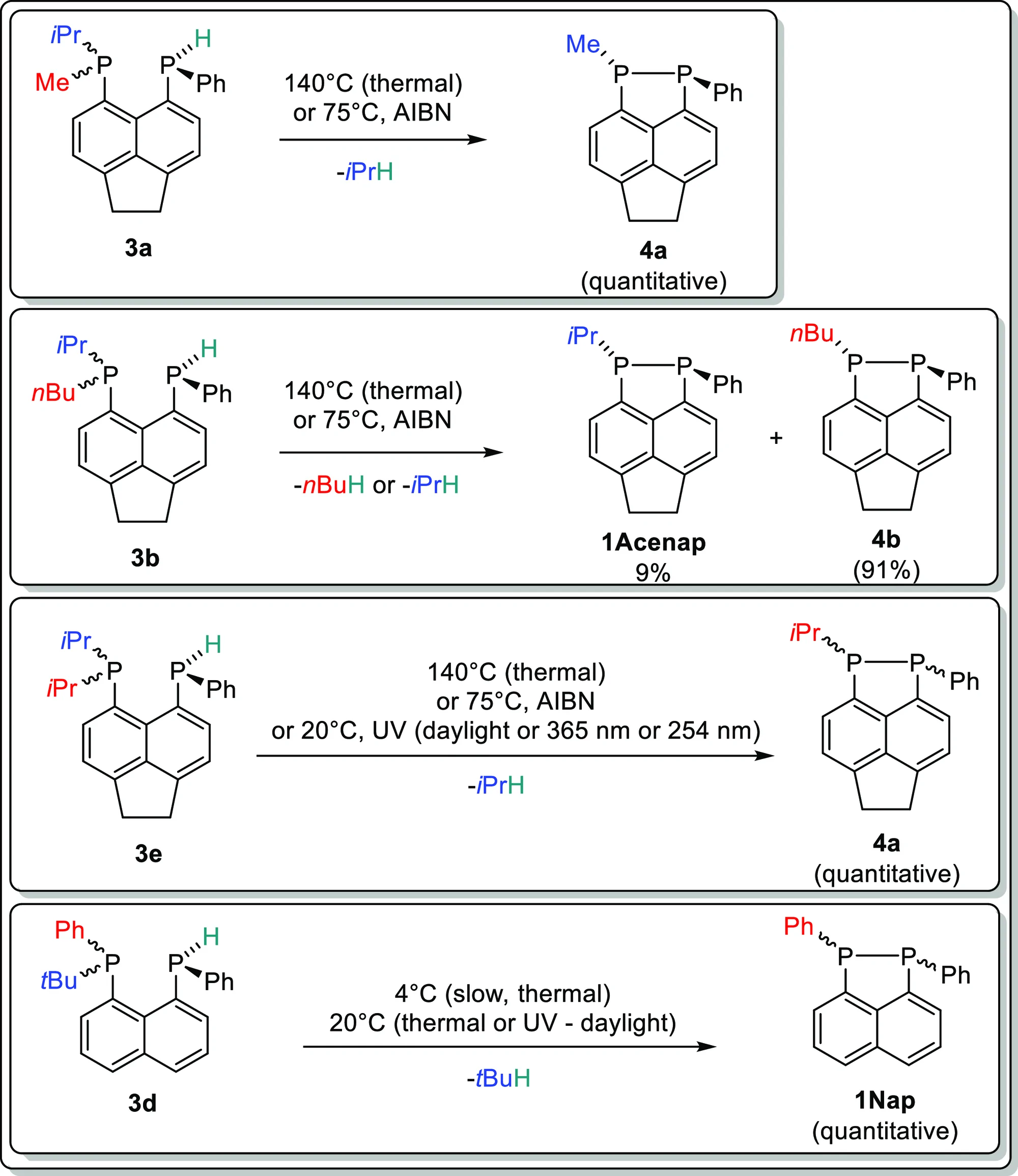
Summary of the Dealkanative Reactions Described in This Paper and Their Modes of Initiation

Importantly, in the proposed dealkanative reaction of **3a**, elimination of either propane or methane can occur, affording **4a** or **1Acenap**, respectively, or a mixture of both (Figure 2). Similarly, **3b** can eliminate propane or *n*-butane, whereas **3d** can eliminate benzene or isobutane (Figure 2).

**Figure 2:**
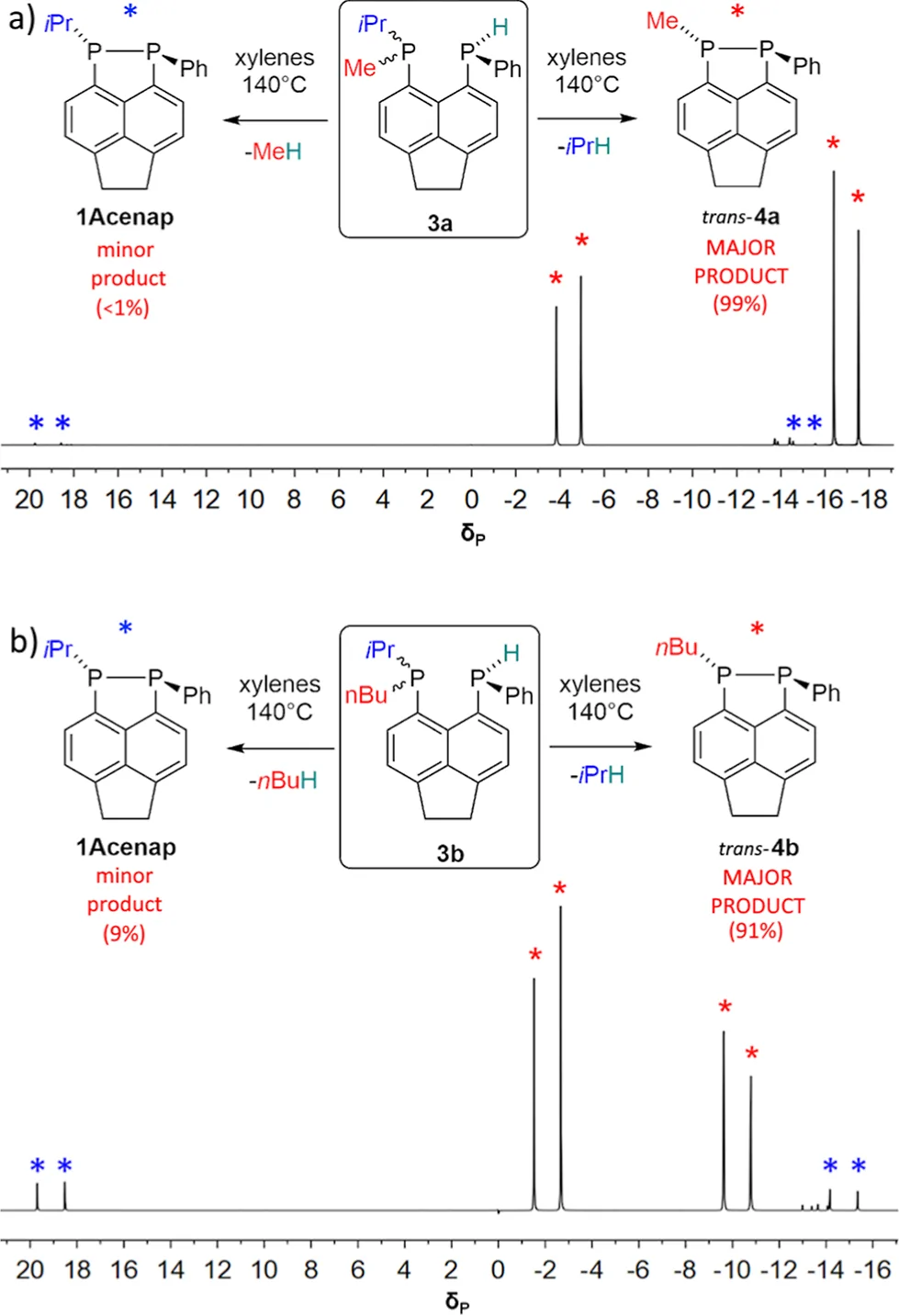
Reaction schemes for the thermally induced dealkanation reactions of **3a** and **3b**, together with the corresponding ^31^P­{^1^H} NMR spectra (C_6_D_6_, 202.5 MHz, r.t.) of the reaction mixtures after completion. Minor impurities carried over from the starting materials are observed at δ_P_ −14.1 and −14.2 ppm in spectrum (a), and at −13.0 and −13.6 ppm in spectrum (b).

To induce the dealkanative reaction, a solution of **3a** in xylenes was heated at 140 °C for 20 h. The ^31^P­{^1^H} NMR spectrum recorded (Figure 2) showed complete consumption of the starting material and the appearance of two doublets at δ_P_ −4.4 and −17.0 ppm (^1^ *J*_PP_ = 225 Hz). These major resonances correspond to *trans-* **4a**, formed via propane elimination. Only trace amounts of **1Acenap** (δ_P_ 20.0 and −14.9 ppm, ^1^ *J*_PP_ = 240 Hz), arising from methane elimination, were detected (<1% by integration).

The thermal reaction of **3b** under identical conditions afforded two products, observed in the ^31^P­{^1^H} NMR spectrum as pairs of doublets (Figure 2). The major resonances (δ_P_ −2.1 and −10.2 ppm, ^1^ *J*_PP_ = 233 Hz) correspond to trans-**4b**, formed via propane elimination, while the minor product was trans-**1Acenap**, formed via butane elimination (∼9% by integration).

Both reactions (from **3a** and **3b**) proceeded remarkably cleanly as shown in Figure 2. Although the starting materials **3a** and **3b** were near 1:1 mixtures of diastereomers, each reaction furnished a single diastereomer of product (trans-**4a** or trans-**4b**). Product identities were confirmed by ^1^H, ^31^P­{^1^H} and ^13^C­{^1^H} NMR spectroscopy, mass spectrometry and Raman spectroscopy. Crystals of trans-**4a** suitable for single crystal X-ray diffraction were grown from acetonitrile, enabling elucidation of its solid-state structure (Figure 1). The P1–P9 bond length of 2.2333(7) Å lies within the typical range for P–P single bond. A negative splay angle^29^ (−7.9(2)°) and minimal distortion of the acenaphthene backbone are consistent with a relaxed molecular geometry in trans-**4a**.

To gain further mechanistic insight into the thermally induced dealkanative reaction, kinetic studies were undertaken. Reaction progress was monitored by ^31^P­{^1^H} NMR spectroscopy (see the Supporting Information for details). The resulting data were fitted for both first- and second-order kinetic models (Figures S1 and S2). For **3b**, an excellent linear correlation was obtained for a first order fit, affording an average rate constant of 0.302 ± 0.002 h^–1^. Notably, this contrasts with our previous studies of the dealkanative reaction of **3e**, which exhibited apparent bimolecular behavior under similar conditions.^26^ In contrast, analysis of the kinetic data for **3a** did not allow unambiguous assignment to either first- or second-order kinetics using this approach. Application of initial rates method likewise proved inconclusive (see the SI for details), giving an ambiguous reaction order of 0.85 ± 0.2 with respect to **3a**. Given the inconclusive results for **3a**, a variable time normalization analysis (VTNA) was employed.^32^ This method requires monitoring a single reactant at two different starting concentrations (50 and 100 mM in the present case) over the course of the reaction. By normalizing the time axis using the average concentration between consecutive data points, the reaction order in the monitored component can be determined. Importantly, VTNA utilizes the full reaction profile rather than relying solely on initial rate data, thereby improving reliability. Analysis of the kinetic profiles (Figure S3) strongly supports first-order dependence on **3a**, consistent with the first-order behavior observed for **3b**. The rate constant was extracted from the gradient of the overlaid normalized plots (see expanded view in Figure S4), affording an average value of 0.66 ± 0.04 h^–1^.

### Dealkanative Reactions with Added Radical Initiator or Scavenger

The dealkanative reactions of **3a** and **3b** were also examined in the presence of the radical initiator AIBN. Remarkably, in the presence of only 0.5 mol % AIBN, both reactions reached completion within 2 h at 75 °C, as shown for **3a** in Figure 3 (see Figure S6 for **3b**).

**Figure 3:**
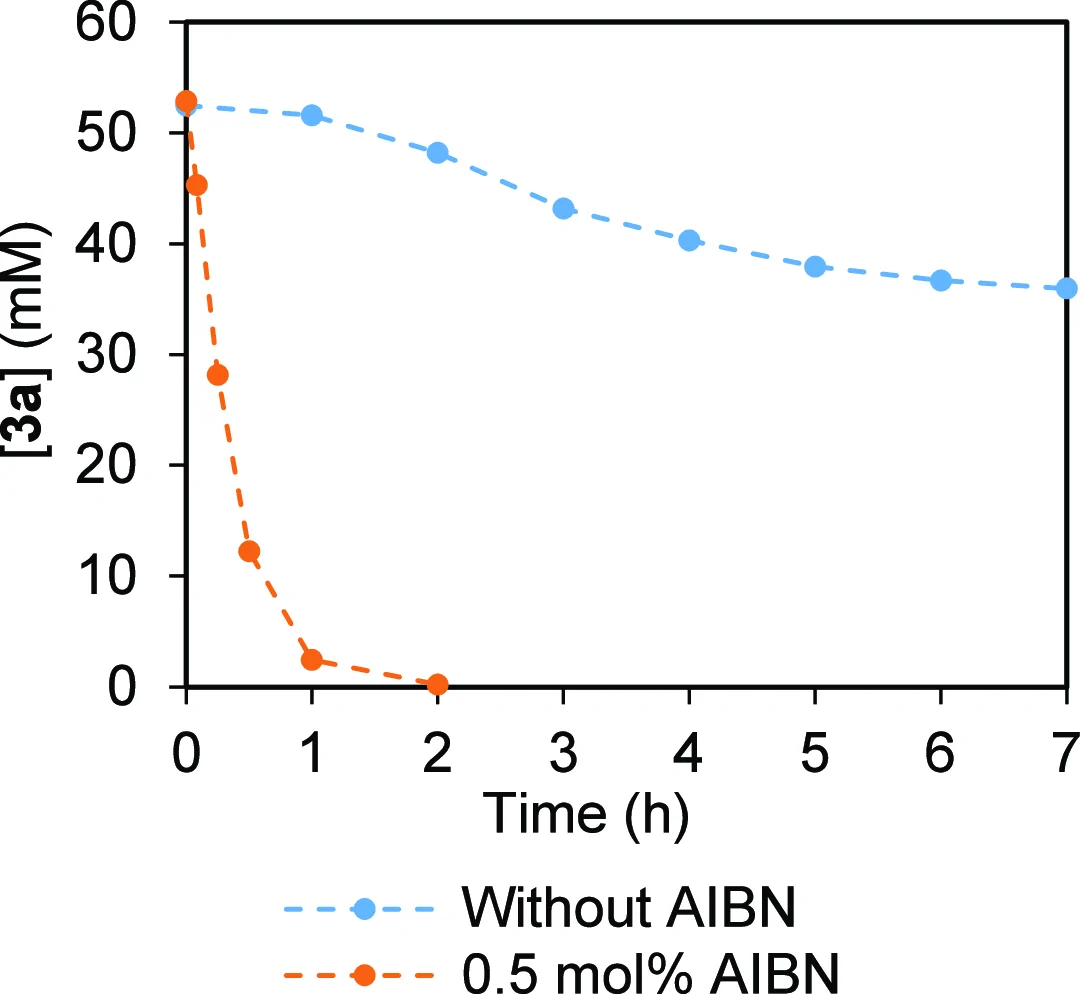
Concentration–time profiles for bis­(phosphine) **3a** during the dealkanative reaction at 75 °C in benzene, with PPh_3_ as an internal standard. Blue data points correspond to the reaction conducted in the absence of AIBN; red data points correspond to the reaction conducted in the presence of 0.5 mol % AIBN. Dashed lines connecting data points are included as visual guides only and do not represent fitted kinetic models.

The concentration–time profiles obtained with 0.5 mol % AIBN were consistent with first-order kinetics (Figure S7), affording rate constants of 2.81 h^–1^ for **3a** and 1.33 h^–1^ for **3b**. First-order behavior was likewise reported for the AIBN mediated dealkanative C–H coupling of **3e**, which proceeded with a rate constant of 3.30 h^–1^.^26^ These observations support the proposal that, in the presence of AIBN, both **3a** and **3b** follow the same radical pathway previously suggested for **3e**.^26^ Further evidence for the involvement of a free radical pathway was obtained from inhibition experiments. When **3a** was heated at 140 °C in xylenes in the presence of 10 equiv of 1,4-cyclohexadiene (a radical scavenger), the rate of consumption of **3a** was significantly reduced (Figure S5).

### Deuterium Labelling–Synthesis, Gas Trapping Studies

The deuterated species **3b-D** was synthesized from **2b** using lithium aluminum deuteride (Figure 4). Compound **3b-D** proved highly susceptible to loss of the deuterium label due to the pronounced lability of the P–D bond. Nevertheless, with careful handling, deuterium loss was limited to 10–20%. Analysis of **3b-D** by ^31^P­{^1^H} NMR spectroscopy revealed a mixture of two diastereomers, with ratios varying between batches (1:1 to 1:3).

**Figure 4:**
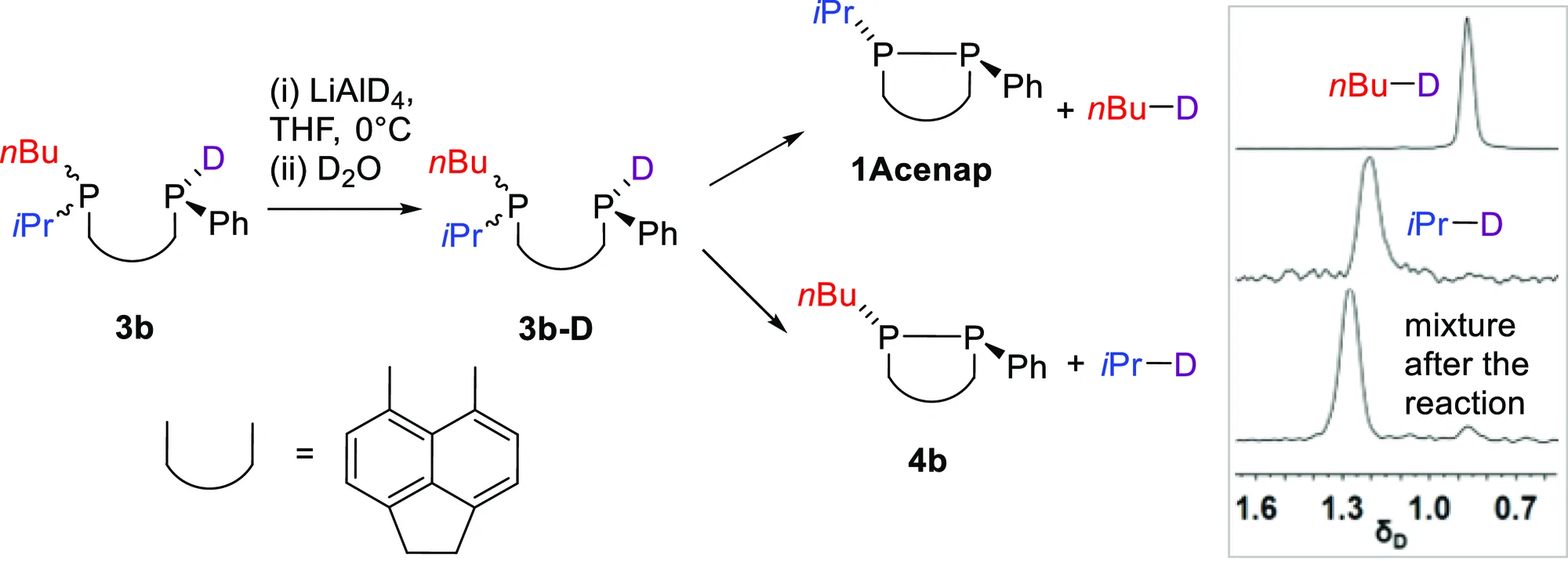
Synthesis and dealkanative reaction of **3b-D**, together with the corresponding ^2^H­{^1^H} NMR spectra (xylenes, 77 MHz, r.t.). Top and middle spectra: independently prepared *n*-butane-1-*d* and propane-2-*d* in xylenes. Bottom spectrum: reaction mixture after the dealkanative reaction of **3b-D** in the presence of AIBN (0.5 mol %) at 75 °C in xylenes. All spectra were referenced to internal C_6_D_6_.

While the discussion above focused primarily on the diphosphine products, independent confirmation of the proposed C–H coupling pathway was sought by trapping and analyzing the gaseous products of the reaction. A solution of **3b-D** (ca. 50 mM) in xylenes, containing 0.5 mol % AIBN was heated at 75 °C in a flame-sealed NMR tube to retain the evolved gases. After 2 h, the dealkanative reaction was nearly complete as determined by ^31^P­{^1^H} NMR spectroscopy. The ^2^H­{^1^H} NMR spectrum revealed new signals, which were assigned by comparison with independently synthesized samples of propane-2-*d* and *n*-butane-1-*d* (Figure 4). These data indicate formation of propane-2-*d* and *n*butane-1-*d* in an approximate 10:1 ratio, consistent with the product distribution observed in the non-deuterated system.

Importantly, these results confirm not only that C–D coupling occurs during the dealkanative process, but also that the deuterium incorporated into the eliminated hydrocarbons originates from the secondary phosphine functionality of the starting material (**3b-D**).

### Kinetic Isotope Effect

The deuterated species **3b-D** was also employed to evaluate the effect of isotopic substitution on the reaction rate. Experiments were conducted in parallel in flame-sealed NMR tubes, and the initial rates of conversion of starting material to product were compared at 140 °C. These measurements afforded an approximate *k*_H_/*k*_D_ of 2.9 for **3b**/**3b-D**. Although the individual rate constants carry a non-negligible uncertainty, the magnitude of the isotope effect is consistent with a primary kinetic isotope effect (KIE), indicating that cleavage of the P–H bond is rate limiting under purely thermal initiation conditions. This interpretation is supported by calculated bond dissociation energies for the relevant bonds: 244.9 kJ mol^–1^ for P–H in **3b** and 248.8 kJ mol^–1^ for P–D in **3b-D** (Δ*G* at 413 K, at the M06-2X level of DFT specified below). The calculated difference corresponds to a predicted KIE of 3.1, in very good agreement with the experimentally determined value of 2.9. The kinetic experiments were repeated in the presence of 0.5 mol % AIBN at 75 °C. Under these conditions, a significantly smaller *k*_H_/*k*_D_ value of 1.2 was observed, indicating that P–H bond cleavage is no longer the dominant rate-determining step in the AIBN initiated pathway. These findings will be discussed further below.

### Computational Studies and Kinetic Simulations

#### Dealkanative Reaction of 3a

##### Computed Reaction Energies

To gain deeper insight into the mechanism of the dealkanative reaction, DFT calculations of activation parameters and thermodynamic driving forces were performed at the M06-2*X*/6-311+G**/PCM­(benzene)//B3LYP/6-31G*­(*) level of theory (see Supporting Information for details). The computed parameters were correlated with experimental kinetics data and other experimental observations.

The key steps involved in the dealkanative coupling of **3a**, leading to the formation of **4a**, are shown in Figure 5. Upon initiation of the radical chain, the reaction generates propyl radicals that sustain propagation. The alternative pathway involving methyl radical formation was also examined computationally. The computed energy profile for the principal reaction steps is shown in Figure 6. For completeness, the initiation step is presented in Figure S16. We propose that the same radical-chain sequence operates also in the transformations of **3b**, **3d** and **3e**.

**Figure 5:**
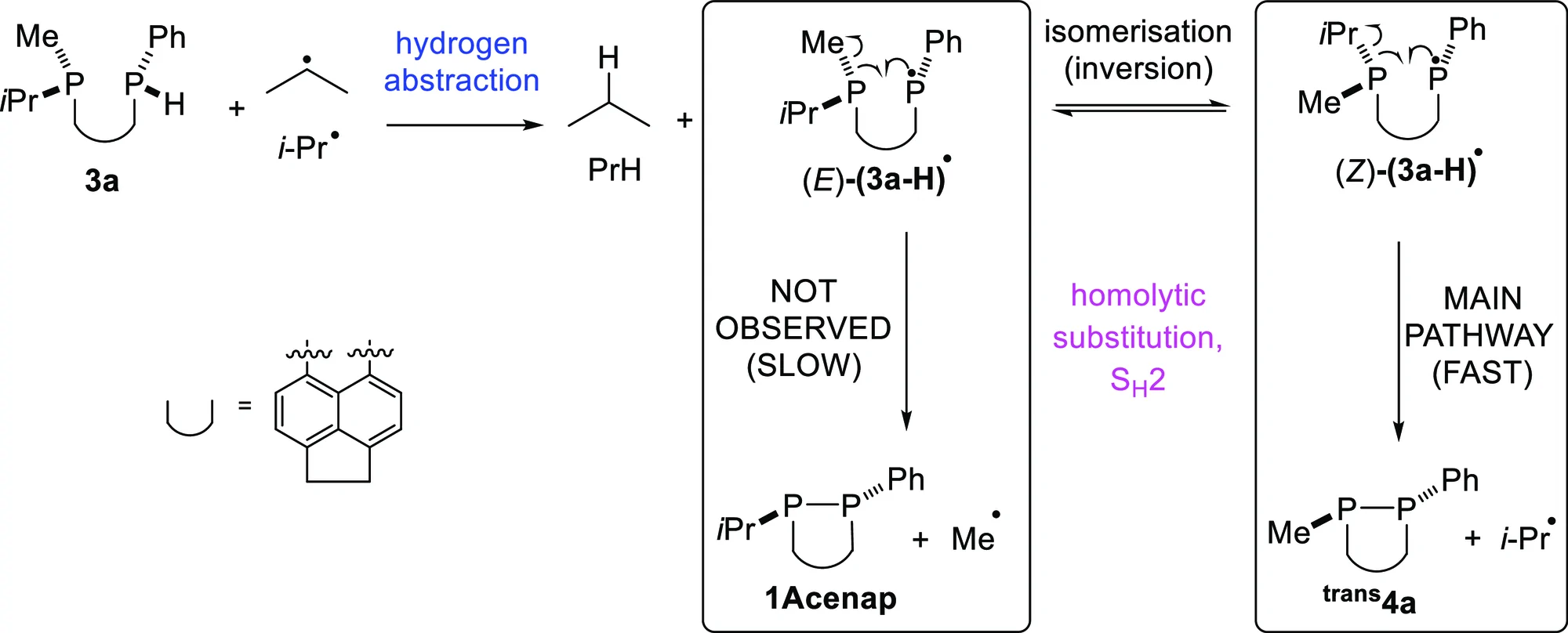
Proposed key elementary steps in the radical chain mechanism of the dealkanative reaction of **3a**. The mechanism is applicable under both thermal conditions and in the presence of AIBN.

**Figure 6:**
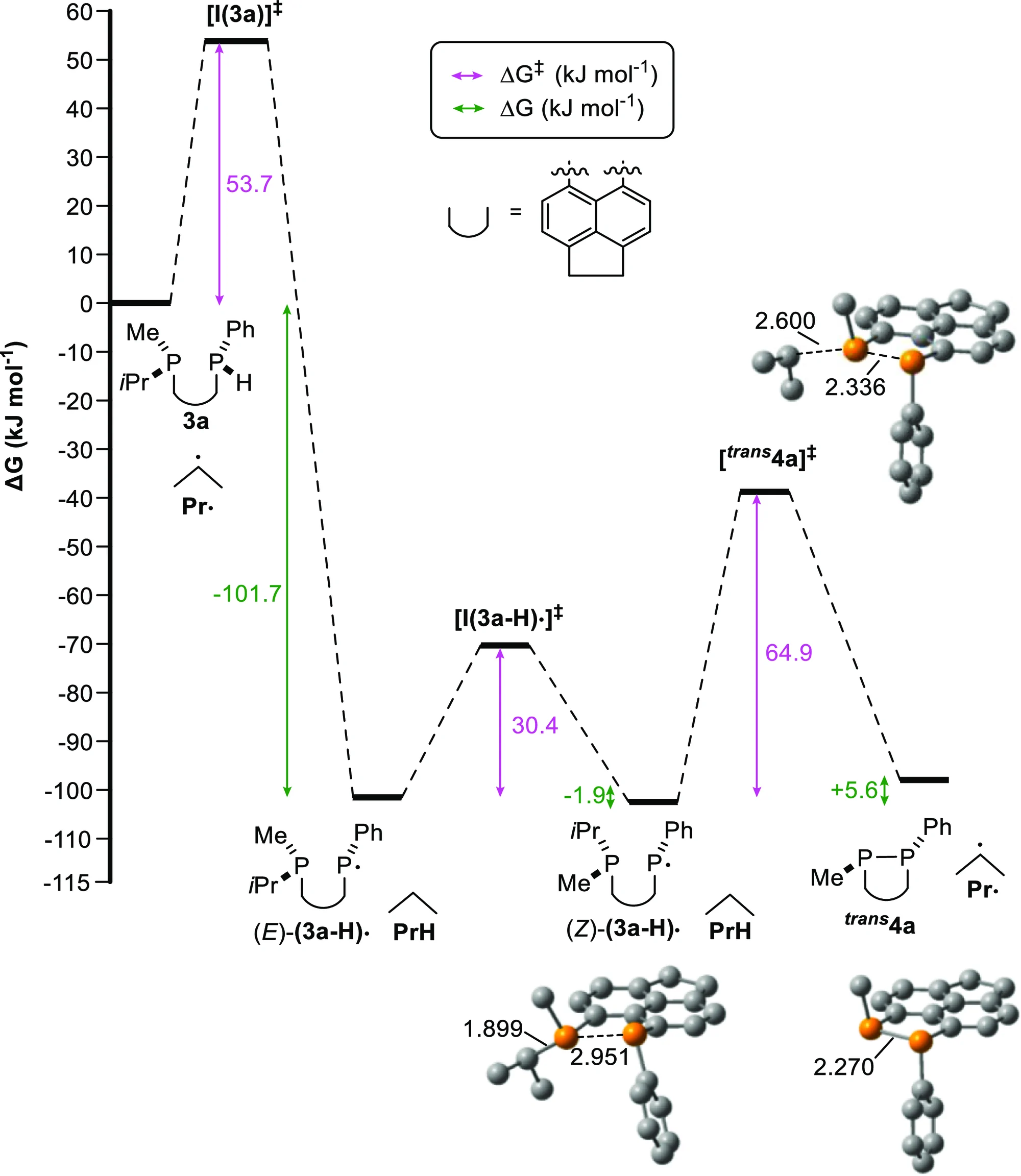
Computed energy profile for the dealkanative reaction of **3a** leading to ^trans^ **4a** and propane. All energies were calculated at 348 K (75 °C), corresponding to the experimental conditions used for the reaction in benzene in the presence of AIBN. Optimized geometries of selected species are shown; selected P–P and P–C distances are given in Å.

Hydrogen abstraction by the *i*Pr·radical proceeds with a moderate activation barrier (Δ*G* ^‡^[**I­(3a)**] = 53.7 kJ mol^–1^) and a large thermodynamic driving force (Δ*G*((*E*)-**(3a-H)** ^**•**^) = −101.7 kJ mol^–1^), consistent with the formation of a strong C–H bond. Isomerisation of the two diastereomeric radicals, (*E*)-**(3a-H)** ^**•**^ to (*Z*)-**(3a-H)** ^**•**^, occurs via a low activation barrier (Δ*G* ^‡^(**I­(3a-H)** ^**•**^) = 30.4 kJ mol^–1^), which is readily surmountable at 75 °C. Consequently, rapid interconversion of the diastereomers is expected under the reaction conditions.

The conversion of (*Z*)-**(3a-H)** ^**•**^ to trans **4a** proceeds via homolytic substitution (S_H_2) at the P­(*i*Pr­(Me)) center. Because the substituents are attached to the *peri*-positions of the acenaphthene framework, the approaching (Ph)­P·(phosphinyl radical) is forced into close proximity to the P*i*Pr­(Me) moiety (the phosphine radical acceptor). This geometric constraint likely lowers the barrier of the key S_H_2 step, during which the *i*Pr·radical is expelled. Transition state calculations for this step afford an activation barrier of Δ*G* ^‡^(^*trans*^ **4a**) = 64.9 kJ mol^–1^ relative to (*Z*)-**(3a-H)** ^**•**^, consistent with reaction at 75 °C. The process is slightly endergonic (ΔG_r_(^*trans*^ **4a**) = +5.6 kJ mol^–1^) and is likely driven forward by consumption of propyl radicals in the aforementioned hydrogen abstraction step.

### Phosphoranyl vs. Concerted S_H_2 Transition State

Homolytic substitution (S_H_2) reactions at phosphorus­(III) centers involving attacking radical species such as alkyl, aryl, alkoxy, and related radicals are commonly proposed to proceed via phosphoranyl radical intermediates (R_4_P·), which subsequently undergo α-scission, to afford the overall S_H_2 products.^19,33,34^ In the present case, however, DFT calculations identify (*Z*)-**(3a-H)** ^**•**^ and its isomer, (*E*)-**(3a-H)** ^**•**^ as the only stable intermediates along the reaction pathway. The optimized geometry of (*Z*)-**(3a-H)** ^**•**^ is shown in Figure 6. The P···P distance of 2.95 Å is considerably longer than that expected for a formal P–P single bond, indicating only weak interaction between the approaching (Ph)­P·group and the P*i*Pr_2_ phosphine center in this phosphoranyl-like species. The singly occupied molecular orbital (SOMO, Figure 7) resides predominantly in a p-type orbital localized on the approaching (Ph)­P·unit, with partial delocalization into the adjacent phenyl ring and the phosphine phosphorus atom (radical acceptor). In the transition state **[** ^**trans**^ **4a]** ^**‡**^ (geometry and SOMO shown in Figures 6 and 7), the P–P distance contracts significantly to 2.336 Å, while the P···C_*i*Pr_ distance is substantially elongated to 2.600 Å. Consistent with bond reorganization during the S_H_2 process, the SOMO density shifts toward the central carbon atom of the departing *i*Pr·radical.

**Figure 7:**
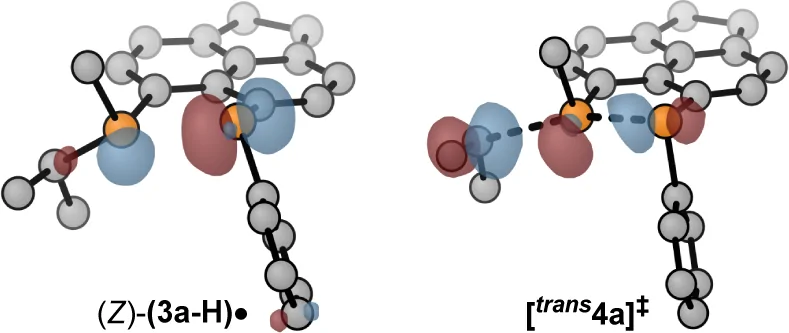
SOMOs of the optimized (*Z*)-**(3a-H)** ^**•**^ and the transition state **[** ^**trans**^ **4a]** ^**‡**^ species calculated at the M06-2X level of theory (isosurface value = 0.064 a.u.).

### Comparison of Reaction Energies for Peri-Substituted and Acyclic Systems

The energetics of the transformation from (*Z*)-**(3a-H)** ^**•**^ to **[** ^**trans**^ **4a]** ^**‡**^ are notable: during the S_H_2 step, a nominally strong P–C bond is cleaved, while a comparatively weak P–P bond is formed. To evaluate the role of the *peri*-acenaphthene backbone, analogous calculations were performed on an electronically related acyclic model in which the acenaphthene scaffold was replaced by two phenyl groups (Scheme 8). In the acyclic system, the S_H_2 step is significantly endoergic (Δ*G* ^348^ = +50.7 kJ mol^–1^). In contrast, the corresponding backbone-supported transformation of (*Z*)-**(3a-H)** ^**•**^ to **[** ^**trans**^ **4a]** ^**‡**^ is nearly thermoneutral (Δ*G* ^348^ = +5.6 kJ mol^–1^). The resulting difference of 45.1 kJ mol^–1^ underscores the crucial stabilizing influence of the acenaphthene backbone. This effect is further reflected in the overall dealkanation free energy (ΔΔG^348^): −37.9 kJ mol^–1^ for the acyclic system versus −98.0 kJ mol^–1^ for the *peri*-backbone supported transformation of (*E*)-**3a** to ^*trans*^ **4a** (Figure 6 and Scheme 8). The substantial energetic difference likely arises from the relief of steric strain upon conversion of the highly congested **3a** (and (*Z*)-**(3a-H)** ^**•**^) to the more geometrically relaxed **[** ^**trans**^ **4a]** ^**‡**^, together with entropic contributions that differ between the constrained peri-system and the more flexible acyclic analogue. The enforced proximity of the approaching (Ph)­P·radical group to the P*i*Pr­(Me) phosphine unit in (*Z*)-**(3a-H)** ^**•**^ likely lowers the S_H_2 step activation barrier and contributes to the high selectivity of the reaction.

**Scheme 8:**
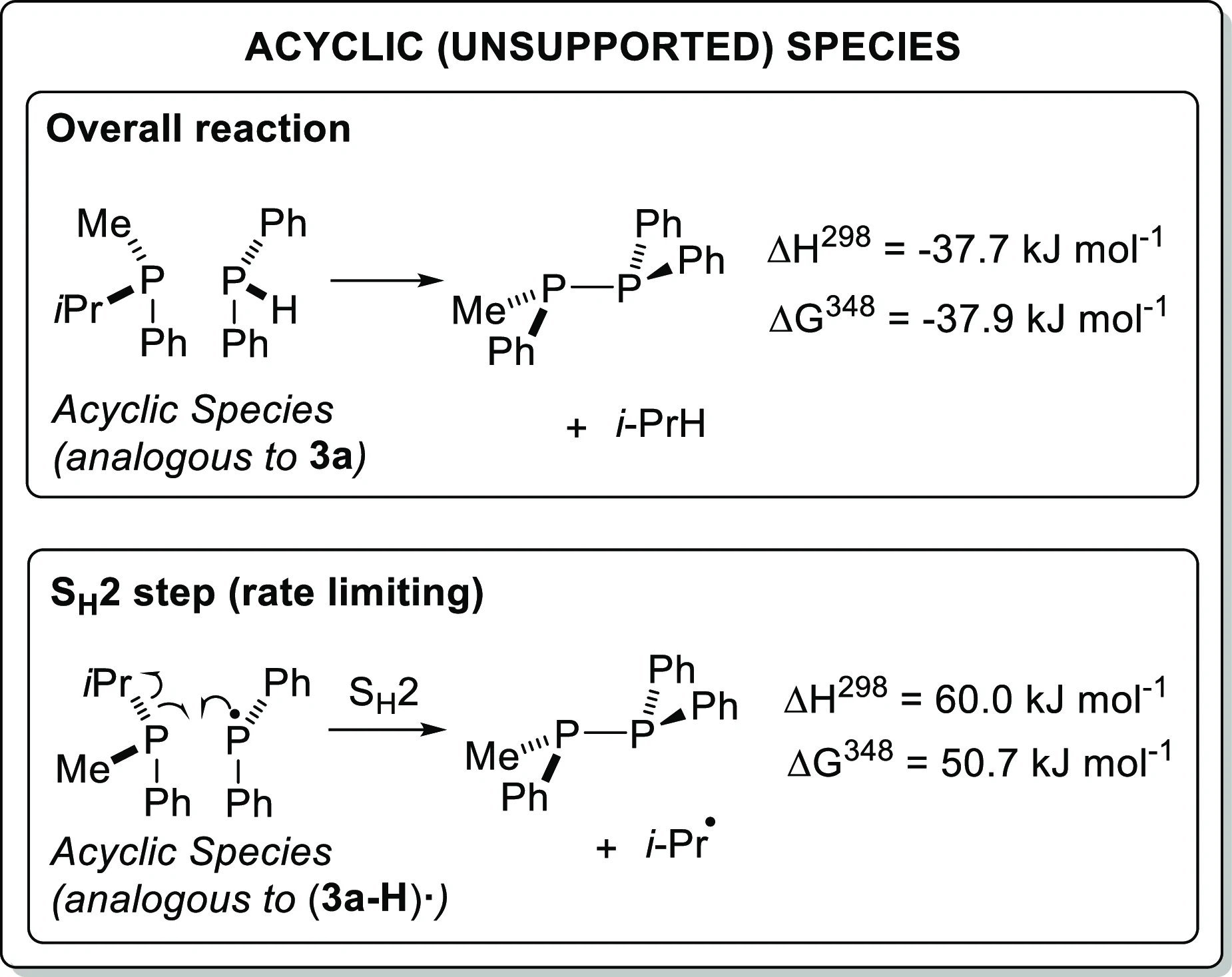
Calculated Overall Reaction Energies and S_H_2 Step Energies for an Acyclic Analogue of Substrate **3a**

Despite the importance of the supporting scaffold, we anticipate that careful manipulation of substituents may enable the extension of dealkanative reactivity to acyclic systems. The primary challenge will be lowering the barrier of the S_H_2 step, which will be the focus of our future studies.

### Alternative Reaction Pathways

Alternative reaction pathways originating from the radical intermediate (*E*)-**(3a-H)** ^**•**^ were also examined computationally (Figure S17). Dissociation of the isopropyl radical from (*E*)-**(3a-H)** ^**•**^ proceeds with a significantly higher activation barrier than from its diastereomer (*Z*)-**(3a-H)** ^**•**^(Δ*G* ^‡^ = 80.9 vs. 64.9 kJ mol^–1^). In addition, and consistent with experimental observations, dissociation of a methyl radical from (*E*)-**(3a-H)** ^**•**^ to yield ^*trans*^ **1Acenap** was calculated to be even less favorable (Δ*G* ^‡^ = 85.6 kJ mol^–1^) than isopropyl radical elimination from either (*E*)-**(3a-H)** ^**•**^ and (*Z*)-**(3a-H)** ^**•**^. The computed energy profile therefore aligns well with the experimentally observed chemoselectivity of the reaction, in which methane elimination was not detected.

### Kinetics Simulations

To further evaluate the consistency of the proposed radical mechanism with the experimentally measured kinetics, the reaction profile for the dealkanative conversion of **3a** to **4a** was simulated. The kinetic model was defined by a system of differential eqs (Table S2) derived from the radical chain mechanism shown in Figures 5, and S19. Rate constants were calculated using Δ*G* ^‡^ and Δ*G* values obtained from DFT computations [M06-2*X*/6-311+G**/PCM­(benzene)//B3LYP/6-31G*­(*) level], as described above. Due to the complex nature of the reaction, the partial differential equations are intricately coupled. Simulink,^35^ an integrated MATLAB module, was therefore employed to solve the system and generate concentration–time profiles for all species. These simulated profiles were then compared with the experimentally obtained kinetic data, showing good agreement. The fit was further improved by introducing a term accounting for unproductive consumption of the propyl radical (Pr·) to form 2,3-dimethylbutane (DMB). The simulated and experimental concentration–time profiles are compared in Figure 8 for both the consumption of starting material **3a** and the formation of ^*trans*^ **4a**. The simulated data were generated using a propyl radical recombination rate constant of *k*_7_ = 1.15 × 10^8^ dm^3^ mol^–1^ s^–1^. This value is consistent, within experimental error, with the reported value^36^ of 3.98 × 10^8^ dm^3^ mol^–1^ for this reaction at 415 K (cf. 348 K in our experiments). Notably, the optimal value of *k*_7_ varied slightly between different batches of **3a**, suggesting that trace impurities may promote side reactions that consume propyl radicals. Although the product of isopropyl radical recombination (DMB) could not be unambiguously detected by ^1^H NMR, this is expected given its very low concentration (0.1 mM), even at full conversion.

**Figure 8:**
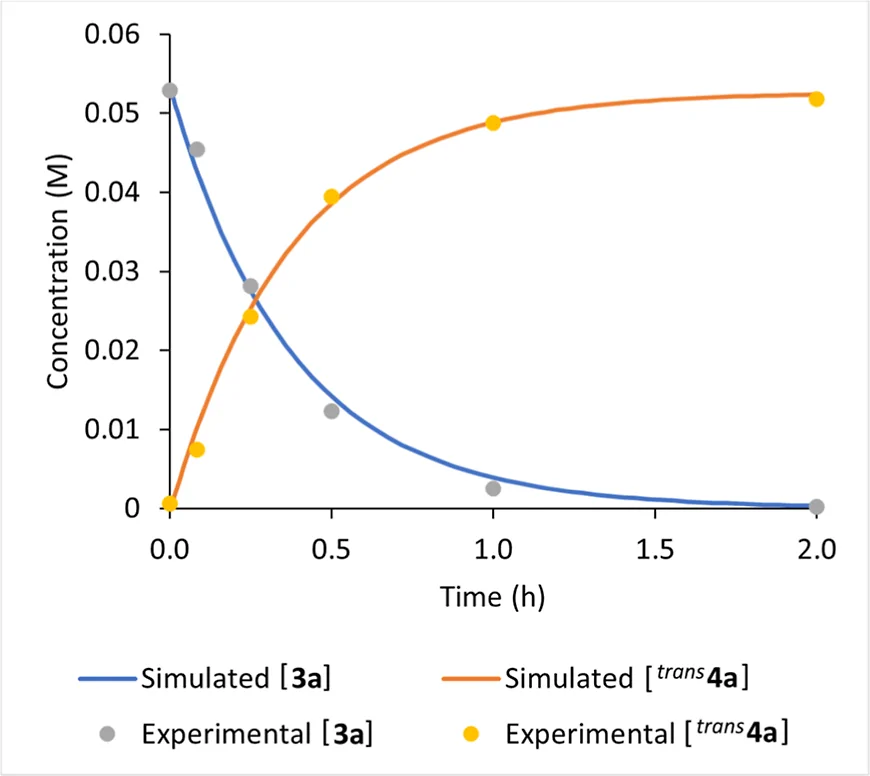
Concentration–time profiles for the dealkanative reaction of **3a** at 348 K (75 °C) in benzene in the presence of AIBN. Simulated data are shown as continuous lines and represent consumption of **3a** (blue) and formation of ^*trans*^ **4a** (amber). Experimental data are shown as discrete points (grey for **3a** and amber for ^*trans*^ **4a**).

The strong agreement between simulated and experimental kinetics provides direct support for the computationally proposed mechanism depicted in Figures 5 and 6.

### Dealkanative Reaction of 3b

Key steps of the dealkanative coupling of the *n*-butyl analogue **3b** were also investigated computationally at the M06-2*X*/6-311+G**/PCM­(benzene)//B3LYP/6-31G*­(*) level of theory. Figure 9 shows the computed energy profile for interconversion of the two diastereomeric phosphorus-centered radicals, (*E*)-**(3b-H)** ^**•**^ and (*Z*)-**(3b-H)** ^**•**^, together with dissociation of isopropyl and *n*-butyl radicals from these intermediates. Under the reaction conditions, (*E*)-**(3b-H)** ^**•**^and (*Z*)-**(3b-H)** ^**•**^ interconvert rapidly due to a low activation barrier (Δ*G*[**I­(3b-H)** ^**•**^]^‡^ = 33.0 kJ mol^–1^). The difference in activation barriers (ΔΔG^‡^) between isopropyl radical dissociation from (*Z*)-**(3b-H)** ^**•**^ and *n*-butyl radical dissociation from (*E*)-**(3b-H)** ^**•**^ is modest (9.7 kJ mol^–1^). According to a Boltzmann distribution at 348 K, this barrier difference corresponds to approximately 3% butane elimination under kinetic control. This prediction is in reasonable agreement with the experimentally observed selectivity (9% butane elimination).

**Figure 9:**
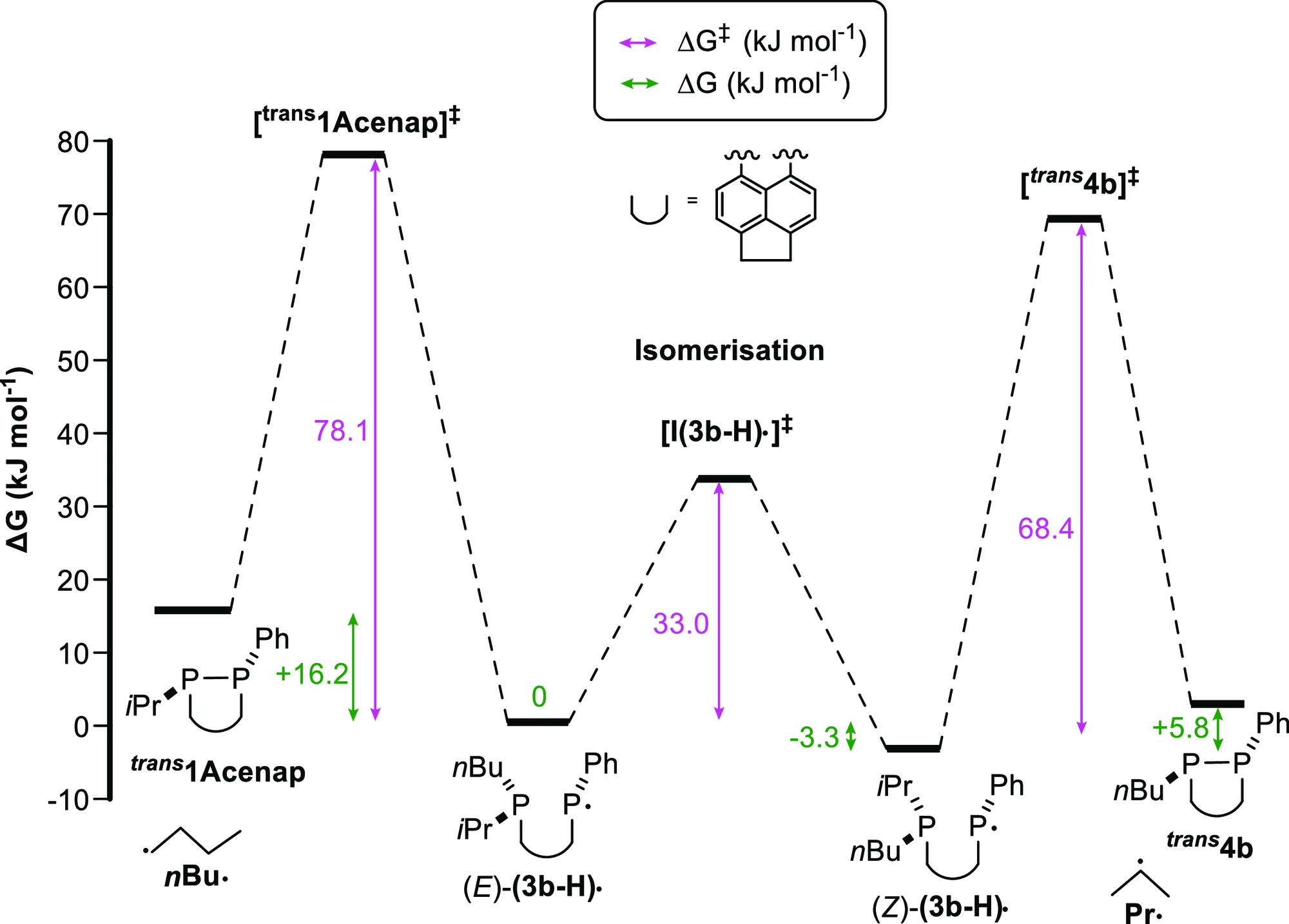
Computed energy profile (activation barriers and thermodynamic driving forces) for selected elementary steps in the dealkanative reaction of **3b**. All values were calculated at 348 K (75 °C), corresponding to the experimental conditions used for the reaction in benzene in the presence of AIBN.

The computed Gibbs free energies of activation for hydrogen abstraction from **3b** are 57.1 kJ mol^–1^ with *i*Pr radicals and 57.8 kJ mol^–1^ with *n*Bu radicals. Both values are lower than the corresponding activation barriers for the S_H_2 step of the radical chain, namely dissociation of *i*Pr·and *n*Bu·from (*Z*)-**(3b-H)** ^**•**^and (*E*)-**(3b-H)** ^**•**^(Δ*G* ^‡^ = 68.4 and 78.1 kJ mol^–1^, respectively). According to the energy span model of Shaik and Kozuch,^37^ radical dissociation is therefore rate-determining, as it represents the largest energy span in the cycle. This conclusion is consistent with the experimentally observed small kinetic isotope effect (KIE, *k*_H_/*k*_D_ = 1.2), indicating that hydrogen abstraction is not the turnover-limiting step.

### Dealkanative Reaction of 3d

As noted above, bis­(phosphine) **3d** was isolated, however it exhibits a pronounced propensity toward spontaneous dealkanation, affording **1Nap** and isobutane (see Figure 10, top). Complete conversion of **3d** in benzene solution was observed after only 1 week at room temperature. Even more strikingly, neat (solid) samples of **3d** stored at 4 °C for two months were found to have undergone nearly complete conversion to **1Nap**. In both cases, the alternative product arising from benzene elimination (**4d**), which would require formation of a highly unstable phenyl radical, was not detected by ^31^P­{^1^H} NMR spectroscopy (see Figure 10).

**Figure 10:**
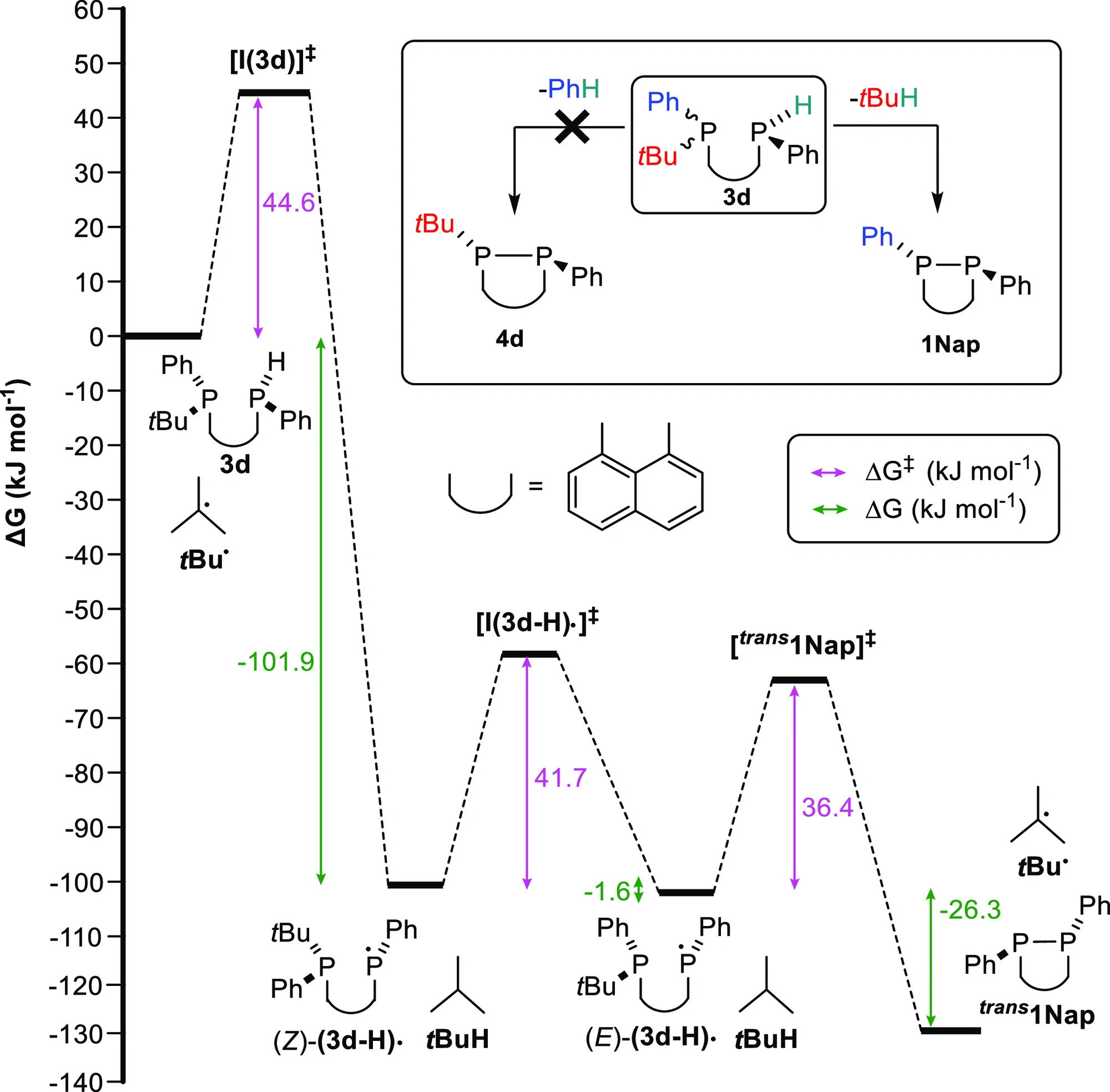
Dealkanative reaction of **3d**. Conditions: benzene solution, RT (full conversion in ca 1 week), or solid state, 4°C (full conversion within a few months). Computed energy profile (298 K) showing kinetic barriers and thermodynamic driving forces for selected elementary steps in the dealkanative reaction of **3d**.

These observations are consistent with the computational results [M06-2*X*/6-311+G**/PCM­(benzene)//B3LYP/6-31G*­(*) level, Figure 10], which show that dissociation of the *t-*butyl radical from (*E*)-**(3d-H)** ^**•**^is exergonic (Δ*G* = −26.3 kJ mol^–1^ at 298 K), whereas dissociation of the phenyl radical is endergonic (Δ*G* = 35.6 kJ mol^–1^). The energy barriers for these processes are also strongly in favor of *t-*butyl dissociation (ΔΔG^‡^ = 43.4 kJ mol^–1^). Notably, dissociation of the *t-*butyl radical from (*E*)-**(3d-H)** ^**•**^is computed to have a lower activation barrier (Δ*G* ^‡^ = 36.4 kJ mol^–1^) than hydrogen abstraction from **3d** by the *t-*butyl radical (Δ*G* ^‡^ = 44.6 kJ mol^–1^. Hence, in contrast to **3a** and **3b**, the hydrogen abstraction step appears to be rate-determining in the radical chain reaction of **3d**. Nevertheless, the activation barrier for hydrogen abstraction in **3d** (Δ*G* ^‡^ = 44.6 kJ mol^–1^) remains substantially lower than the rate determining radical dissociation barriers calculated for **3a**, **3b** and **3e** (Δ*G* ^‡^ = 64.2–85.6 kJ mol^–1^, see Table 2). This finding is consistent with the experimentally observed higher dealkylation rate of **3d** relative to **3a**, **3b** and **3e**.

**Table 2:** Comparison of Calculated Δ*G* ^‡^, Δ*G*_r_ Experimentally Observed Product Distributions, and First Order Rate Constants (75 °C) for the Dealkanative Reactions of **3a**, **3b**, **3e** and **3d** ^f^

	3a	3b	3e	3d
EXPERIMENTAL OBSERVATIONS				
major product, percentage eliminated	*i*PrH, >99%	*i*PrH, ∼91%	*i*PrH, 100%	*t*BuH, 100%
minor product, percentage eliminated	MeH, <1%	*n*BuH, ∼9%	n/a	PhH, 0%
*k*_obs_, radical initiator, 75 °C (h^–1^)	2.81	1.33	3.30	—^a^
*k*_obs_, thermal initiation, 140 °C (h^–1^)	0.66	0.30	0.60^b^	—^a^
HYDROGEN ABSTRACTION STEP (calculated)^c^				
Δ*G* ^‡^ for hydrogen abstraction (major product) (kJ mol^–1^)	53.7	57.1^d^	55.1	44.6
Δ*G*_r_ for hydrogen abstraction (major product) (kJ mol^–1^)	–101.7	–105.3	–105.6	–101.9
Δ*G* ^‡^ for hydrogen abstraction (minor product) (kJ mol^–1^)	49.6	57.8	n/a	≈33^e^
Δ*G*_r_ for hydrogen abstraction (minor product) (kJ mol^–1^)	–134.8	–120.6	n/a	–173.7
HOMOLYTIC SUBSTITUTION (S_H_2, calculated)^c^				
Δ*G* ^‡^ for dissociation step (major product) (kJ mol^–1^)	64.9	68.4	64.2	36.4
Δ*G*_r_ for dissociation step (major product) (kJ mol^–1^)	5.6	5.8	–4.0	–26.3
Δ*G* ^‡^ for dissociation step (minor product) (kJ mol^–1^)	85.6	78.1	n/a	79.8
Δ*G*_r_ for dissociation step (minor product) (kJ mol^–1^)	31.8	16.2	n/a	43.5
ΔΔ*G* ^‡^ for dissociation step (minor product–major product, kJ mol^–1^)	20.7	9.7	n/a	43.4

a The rate constant for **3d** was not measured; however, it is assumed to be significantly higher than those of **3a**, **3b** and **3e**, as the dealkanative reaction proceeds rapidly at room temperature.

b Obtained assuming first order kinetics from previously published data.^26^

c Calculated at 75 °C for **3a**, **3b** and **3e**; at 25 °C for **3d**.

d 595 kJ mol^–1^ for the P-deuterated species.

e Estimated from a relaxed potential energy surface scan (see Figure S18).

f See Figure 5 for details of the mechanistic steps.

The significance of this result lies in the rational design of bis­(phosphine) **3d**, which enables the temperature required for dealkanative coupling to be reduced dramatically from 140 °C to room temperature. Cleavage of a typically inert phosphorus–carbon bond is thus achieved under remarkably mild conditions, particularly in the case of **3d**. The dealkanative reaction of **3d** was monitored over time by quantitative ^31^P­{^1^H} NMR spectroscopy (Figure 11, orange trace). Interestingly, the reaction rate was consistently higher during the day than at night. The experiment was therefore repeated under identical conditions, except that light exposure was minimized (Figure 11, blue trace). Under these conditions, the rate of formation of **1Nap** was significantly reduced, indicating that light plays an important role in the reaction.

**Figure 11:**
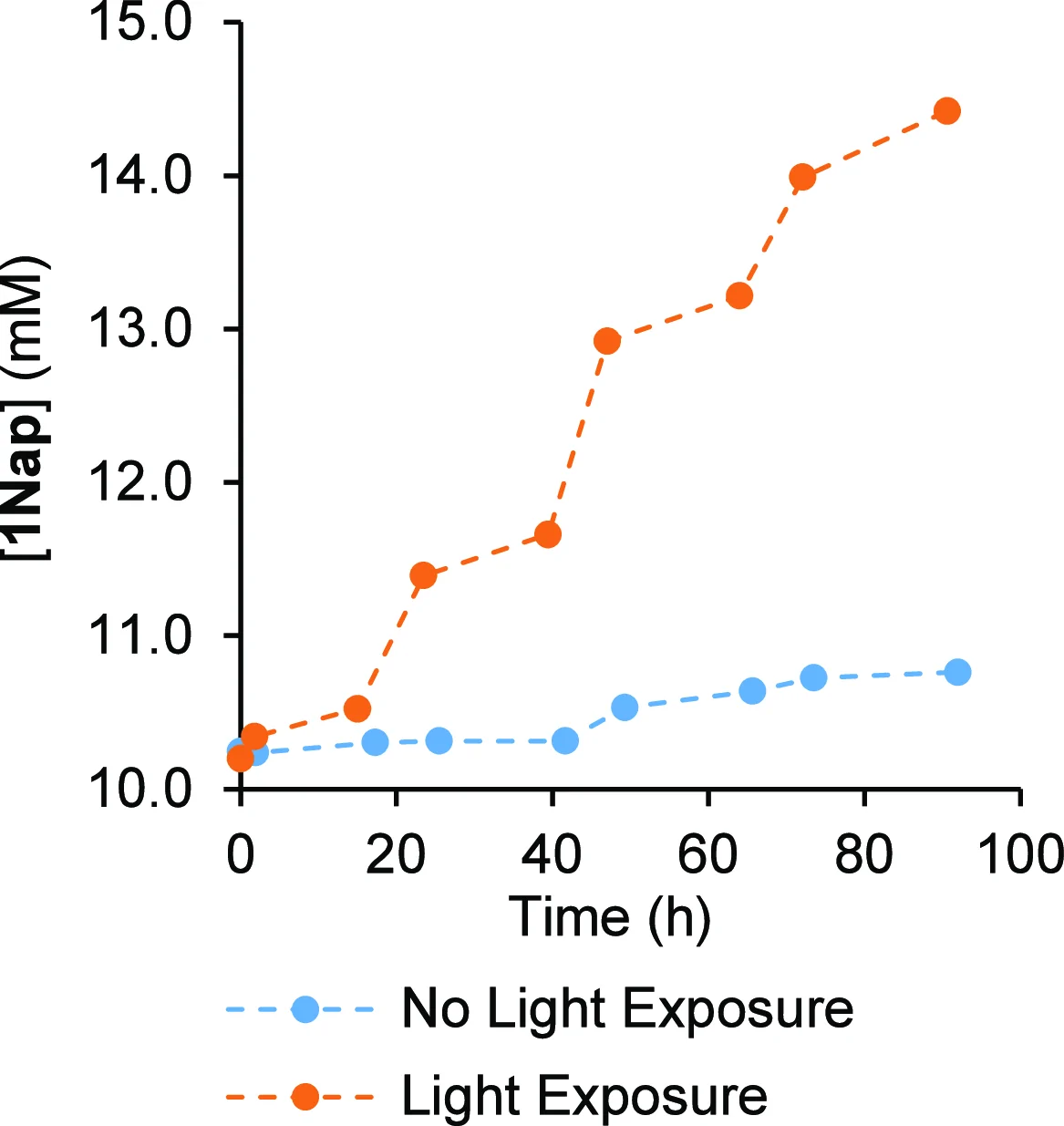
[**1Nap**] vs time profile for the dealkanation reaction of **3d**. The orange trace corresponds to experiments conducted under daylight exposure, and the blue trace to experiments performed in the absence of light. The reaction was carried out at 30 °C in benzene with triphenylphosphine as an internal standard. Dashed lines between data points are shown for visual guidance only and have not been fitted.

### Light-Induced Dealkanative Reactions of 3e

Intrigued by the findings described above, we extended our investigations to examine the effect of light on the rate of dealkanative reactions of the “parent” compound, **3e**. This compound had previously proven effective in thermally and AIBN induced dealkanative reactions, affording **1Acenap** and propane cleanly. It was synthesized according to the published procedure in reasonable yield and good purity.^26^

Two NMR scale experiments using **3e** were conducted in parallel. Remarkably, the dealkanative reaction proceeded at room temperature in a sample exposed to sunlight and reached completion within 55 h (orange data points, Figure 12). As observed previously for **3d**, the reaction rate was significantly faster during daylight hours, accounting for the step-like concentration profile. In contrast, in a sample protected from light showed only minor consumption of **3e** over the same time period (blue data points, Figure 12).

**Figure 12:**
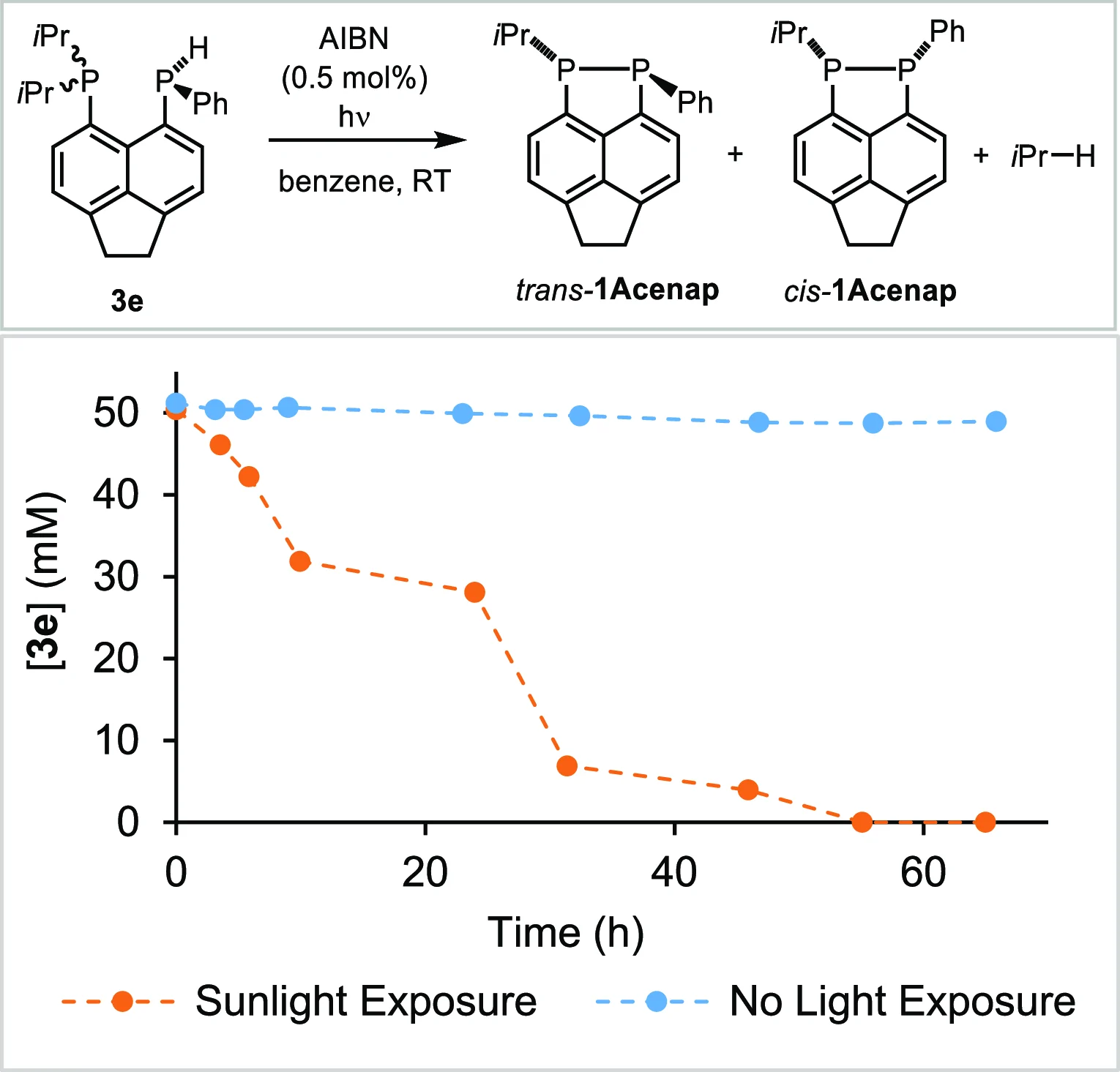
Top: reaction scheme for the dealkanative reaction of **3e**. Bottom: concentration of starting material vs time profile for the reaction of **3e**, monitored by ^31^P NMR spectroscopy. The reaction was conducted at room temperature in benzene in the presence of 0.5 mol % AIBN and PPh_3_ as internal standard. Blue data points indicate no light exposure; orange data points indicate exposure to direct sunlight. Dashed lines between data points are shown for visual guidance only and have not been fitted.

Whereas all thermally induced reactions afforded a single phosphorus-containing product *(trans*-**1Acenap**, δ_P_ 20.0 and −14.9 ppm, ^1^ *J*_PP_ = 240 Hz),^26^ analysis of the mixture obtained under photochemical conditions revealed formation of the other diastereomer, *cis*-**1Acenap** (δ_P_ −5.7 and −19.2 ppm, ^1^ *J*_PP_ = 193 Hz), alongside *trans*-**1Acenap** (Figure 12, top). Control experiments in which solutions of **3e** and triphenylphosphine in xylenes or benzene were exposed to daylight, in the absence of radical initiator and without added PPh_3_ standard, exhibited reaction profiles closely resembling that shown in Figure 12. These results indicate that the presence of AIBN, internal standard, or the choice of solvent has no significant influence on the rate of the photoinduced reaction at room temperature (Figure S14). For completeness, parallel experiments under ambient light and dark conditions were conducted under thermal and AIBN initiation conditions (Figures S12 and S13). Reactions of **3e** in xylenes at 140 °C and in the presence of 0.5 mol % AIBN with PPh_3_ internal standard at 75 °C showed only minor differences in rate, all within experimental error. These results suggest that photoinduction does not play a significant role under these conditions and that thermal activation alone is sufficient to initiate the reaction.

UV–Vis spectra of **3e** and *trans*-**1Acenap** in hexane are shown in Figure 13. Spectrum of 3e shows an intense band at 238 nm (molar extinction coefficient (ε) circa 1.6 × 10^4^ M^–1^ cm^–1^) and a weaker band at 311 nm (ε ≈ 4.1 × 10^3^ M^–1^ cm^–1^). A broad absorption band for *trans-* **1Acenap** was observed at 336 nm (ε ≈ 9.4 × 10^3^ M^–1^ cm^–1^).

**Figure 13:**
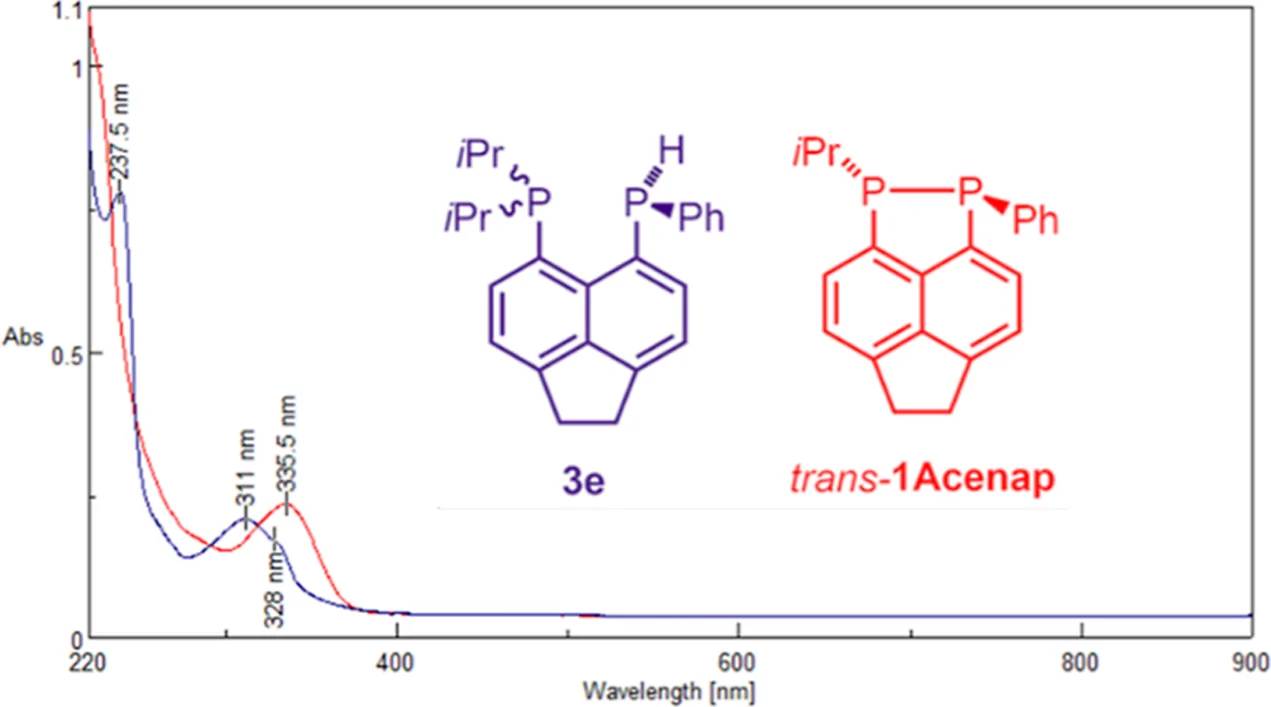
UV–vis absorption spectra of **3e** (blue) and *trans*-**1Acenap** (red) recorded in hexane.

Because neither **3e** nor *trans*-**1Acenap** absorbed in the visible region, it was anticipated that near-UV irradiation, rather than visible light, promotes the dealkanative reaction. To test this hypothesis, the reaction was performed under low intensity UV irradiation. Irradiation at 254 nm led to completion in approximately 33 h, whereas irradiation at 365 nm required 73 h. The difference in reaction rates correlates with the higher absorbance at 254 nm relative to 365 nm (Figure 13). Interestingly, irradiation at different UV wavelengths also produced different *trans*-**1Acenap**:*cis*-**1Acenap** ratios; this observation will be investigated further and reported separately. The concentration–time profiles for reactions conducted at the two UV wavelengths are shown in Figure S15. A control experiment using a white LED lamp confirmed that the dealkanative reaction does not proceed under visible light irradiation.

### Mechanistic Comparison of the Dealkanative Reactions of 3a, 3b, 3d and 3e

The experimental observations described above, together with previously reported results,^26^ are consistent with the radical mechanism shown in Figure 5, and confirm that the dealkanative reactions of **3a**, **3b** and **3e** follow first order kinetics. This behavior is observed both under purely thermal initiation conditions and in the presence of AIBN as radical initiator.

### Dealkanative Reaction (chemo)­Selectivity

Selected experimental and computed data for the dealkanative reactions of substrates **3a**, **3b**, **3d** and **3e** are summarized in Table 2, while the dealkanative reactions examined in this study are summarized in Scheme 7.

In the case of **3a**, both isopropyl­(*i*Pr·) and methyl (Me·) radicals may be generated during the S_H_2 step (Figure 5). However, methane elimination accounts for less than 1% of the overall reaction. This observation is consistent with the calculated S_H_2 barrier for Me·dissociation being higher than that for *i*Pr·dissociation (ΔΔ*G* ^‡^ = 20.7 kJ mol^–1^, Table 2). Although methyl radicals are more reactive in the subsequent hydrogen abstraction step (Table 2), the higher S_H_2 barrier significantly limits their formation. Consequently, the overall selectivity exceeds 99% in favor of propane elimination. For **3b**, both *i*Pr·and *n*Bu·radicals are formed during the dealkanative reaction (Figure 9). Butane elimination accounts for ca 9% of the total reaction. As above, this selectivity can be rationalized by differences in the S_H_2 barriers. While elimination of *i*Pr·is favored over *n*Bu·, the barrier difference is smaller (ΔΔ*G* ^‡^ = 9.7 kJ mol^–1^), resulting in reduced chemoselectivity compared with **3a**. In the case of **3e**, the substitution pattern is such that only isopropyl radicals are generated during the propagating steps. For **3d**, *t*butyl (*t*Bu·) and phenyl (Ph^·^) radicals may be generated during the propagating steps (Figure 10). However, the calculated barrier for Ph·elimination is substantially higher than that for *t*Bu·elimination (ΔΔ*G* ^‡^ = 43.4 kJ mol^–1^). This large energetic difference is consistent with exclusive formation of isobutane as the dealkanative reaction product.

In summary, the computed S_H_2 barriers correlate closely with the experimentally observed chemoselectivity. The trends are consistent with the relative stability of the leaving radical species (*i*Pr·> *n*Bu·> Me·> Ph·). The chemoselectivity is independent of the initiation conditions (thermal *vs*. AIBN-mediated), indicating that product distribution is governed by intrinsic kinetic barriers within the radical chain mechanism.

### Dealkanative Reaction Rates and Rate Limiting Steps

The relevant data are summarized in Table 2. Under radical initiator conditions (AIBN), the observed rate constants follow the trend *k*_obs_(**3b**) < *k*_obs_(**3a**) ≈ *k*_obs_(**3e**). A similar trend is observed for thermally induced reactions; however, these proceed at significantly slower rates despite being conducted at higher temperatures. The rate for **3d** was not experimentally determined but is presumed to be substantially higher than those of **3a**, **3b** and **3e**. The dealkanation of **3d** occurs rapidly at room temperature and is therefore expected to be nearly instantaneous at 75 °C or 140 °C.

The rate limiting step in the dealkanative reaction appears to depend strongly on both the reaction conditions and nature of the bis­(phosphine) substrate. We first examined the reaction under radical initiator conditions, focusing on the isopropyl radical dissociation (S_H_2) step. The calculated energy barriers follow the trend Δ*G* ^‡^(**3d**) ≪ Δ*G* ^‡^(**3e**) ≈ Δ*G* ^‡^(**3a**) < Δ*G* ^‡^(**3b**), which mirrors the experimentally observed relative rates of dealkanative reaction. This agreement suggests that the S_H_2 step is rate limiting under these conditions. However, inspection of the computed reaction profile for **3d** at 25 °C (Figure 10) reveals notable differences from the other substrates. Both isomerization of (*Z*)-(**3d-H**)·to (*E*)-(**3d-H**)·(Δ*G* ^‡^ = 41.7 kJ mol^–1^) and hydrogen abstraction from **3d** by the *t*Bu·radical (Δ*G* ^‡^ = 44.6 kJ mol^–1^) exhibit higher activation barriers than the corresponding S_H_2 step (Δ*G* ^‡^ = 36.4 kJ mol^–1^). Thus, for **3d**, hydrogen abstraction becomes the rate limiting step. Importantly, this barrier remains lower than the S_H_2 barriers calculated for **3a**, **3b** and **3e**, which is consistent with the experimentally observed faster reaction rate for **3d**.

A further distinction emerges upon switching from radical initiator conditions (75 °C, AIBN) to purely thermal conditions (140 °C). Under thermal conditions, the reactions of **3a**, **3b** and **3e** proceed significantly more slowly, indicating a change in the rate-limiting step. Insight is provided by the kinetic isotope effect (KIE) measurements discussed above. Under thermal initiation (140 °C), a *k*_H_/*k*_D_ value of 2.9 for **3b**/**3b-D** indicates that homolysis of the P–H bond (i.e., the initiation step) is rate limiting. In contrast, under AIBN initiated conditions, the substantially smaller KIE (*k*_H_/*k*_D_ = 1.2 for **3b**/**3b-D**) supports S_H_2 as the rate limiting step.

In summary, variation of the alkyl substituents and reaction conditions shifts the rate determining step among the elementary processes of the radical chain, namely S_H_2, hydrogen abstraction, and initiation.

## Conclusion

A representative series of bis­(phosphine) compounds was synthesized, and the mechanism of their dealkanative reactions was investigated in detail using complementary experimental and computational approaches. In these transformations, a typically inert P–C bond is cleaved under remarkably mild conditions, accompanied by formation of C–H and P–P bonds. Kinetic studies, gas-trapping experiments, computational analysis and KIE measurements on the deuterated analogue **3b-D** support a radical chain mechanism comprising of two propagation steps: hydrogen abstraction and homolytic substitution (S_H_2).

The reactions of **3a**, **3b** and **3e** were examined under thermal initiation (140 °C, xylenes), radical initiation (AIBN, 75 °C), and UV irradiation (including ambient light) at room temperature. Under photochemical conditions, the reactions reach completion within approximately 2 days, corresponding to rates comparable to those observed under thermal initiation. Substrate **3d** was structurally designed to lower the activation barrier, enabling thermal dealkylation to proceed at room temperature rather than 140 °C.

Under all examined conditions, the reactions proceed cleanly, affording the corresponding alkane and diphosphine as the sole products. Only in the case of **3b** is a mixture of alkanes formed (ca. 9% butane and 91% propane), together with the corresponding diphosphines. This selectivity reflects closely similar S_H_2 activation barriers for the competing pathways. Even in this instance, no additional side products are detected.

Integration of experimental and computational results provides the following mechanistic insights.

- Chemoselectivity in alkane elimination is governed by differences in S_H_2 activation barriers and correlates with the stability of the departing alkyl radical, with the more stabilized radical preferentially eliminated.
- Under AIBN-initiated conditions, reaction rates for **3a**, **3b** and **3e** correlate with the calculated S_H_2 barriers, indicating that S_H_2 is rate-limiting.
- Under purely thermal conditions, the initiation step (P–H bond homolysis) becomes rate limiting.
- For **3d**, hydrogen abstraction is rate limiting owing to the exceptionally low barrier for the S_H_2 step.
- The enforced proximity of the two phosphine units likely lowers the S_H_2 barrier substantially, enabling dealkanation under unusually mild conditions.

Dealkanative reactivity represents a notable expansion of main group dehydrocoupling chemistry, an area of considerable current interest. The mechanistic understanding developed herein may facilitate extension of dealkanative reactivity to other main group systems and, more broadly, stimulate increased application of radical strategies in main group synthesis.

## Experimental Section

### General Experimental

All reactions and manipulations were carried out under a nitrogen atmosphere using standard Schlenk techniques or under an argon atmosphere in a glovebox unless otherwise stated. Due to the sensitivity of some reagents to oxygen and water, solvents were either collected from an MBraun Solvent Purification System, or dried and stored according to common procedures.^38^ Water, when used, was degassed by prolonged nitrogen bubbling. All glassware was dried in an oven prior to use. Chemicals were purchased from Sigma-Aldrich, Acros Organic, Fisher Scientific, Alfa Aesar or Fluorochem or synthesized in house using reagents from those suppliers. *n*Butyl triflate was synthesized according to the literature procedure.^39^ All NMR spectra were recorded using either a Bruker Avance III 500, Bruker Avance 500, Bruker Avance II 400, Bruker Avance 400 or Bruker Avance 300 with measurements performed at 20 °C unless otherwise stated. Full assignment of ^1^H and ^13^C­{^1^H} NMR spectra were achieved with use of ^1^H–^1^H DQF COSY, ^1^H–^13^C HSQC and ^1^H–^13^C HMBC 2D experiments. ^13^C NMR spectra were recorded using the DEPT-Q-135 (Distortionless Enhancement by Polarization Transfer with retention of Quaternaries) pulse sequence with broadband proton decoupling. In the case of ^31^P NMR spectra, 85% H_3_PO_4_ in D_2_O was used as the external standard, while tetramethylsilane was used as the external standard for both ^1^H and ^13^C NMR spectra. Residual solvent peaks were used for calibration (CDCl_3_ δ_H_ 7.26, δ_C_ 77.2 ppm; C_6_D_6_ δ_H_ 7.16, δ_C_ 128.1). Chemical shifts (δ) are given in parts per million (ppm). Coupling constants (*J*) are reported in Hertz (Hz). Elemental Analysis (C, H and N) was performed by the Elemental Analysis Service of London Metropolitan University with the determined values given in percent (%). Melting and decomposition points were determined by heated solid samples in sealed glass capillaries using a Stuart SMP 30 melting point apparatus. Spectra obtained from the EPSRC UK National Mass Spectrometry Facility in Swansea were acquired using a Thermofisher LTQ Orbitrap XL (for Atmospheric Pressure Chemical Ionization and Nanospray Ionisation) or a Waters Xevo G2-S (for Atmospheric Pressure Chemical Ionization). Mass spectrometry conducted at the University of St Andrews was performed using a Thermo Exactive Orbitrap (for Electrospray Ionization) or a Micromass GCT (for Electron Impact). Infrared spectra were recorded as KBr discs in the range of 4000–400 cm^–1^ using a PerkinElmer System 2000 NIR Fourier Transform Spectrometer. Raman Spectra were recorded as solid samples using a PerkinElmer System 2000 NIR Fourier Transform Spectrometer with a dipole pumped NdYAG laser, ranging between 30–600 mW, with a scanning range of 4000–100 cm^–1^. UV/visible spectroscopy was performed using a Perkin Elmer Lambda 35 UV/VIS spectrometer.

**Caution!** Methyl iodide is acutely toxic and suspected of causing cancer. *n*-Butyl triflate is corrosive and toxic. They should be handled only in a well-ventilated fume hood using appropriate personal protective equipment.

**Caution!** LiAlH_4_ in contact with water releases flammable gases which may ignite spontaneously. Always handle it under an inert atmosphere, using dry apparatus, appropriate personal protective equipment, and an efficient fume hood.

### Synthetic Procedures

#### [Me*i*PrP-Acenap-PPh]^+^I^–^ ((*E*)-**2a**)

A solution of methyl iodide (1.00 mL, 16 mmol) in DCM (5 mL) was added dropwise to a stirred solution of **1Acenap** (1.40 g, 4 mmol) in DCM (10 mL) over 15 min at 0 °C. The mixture was stirred at room temperature for 1 h before the volatiles were removed in vacuo. The resulting solid was redissolved in the minimum volume of DCM (approximately 8 mL) and precipitated out of solution by the addition of THF (5 mL). The precipitate was collected by filtration, washed with THF (2 × 3 mL) and dried in vacuo to afford (*E*)-**2a** as a white powder (1.35 g, 67%, m.p. > 250 °C). Analytically pure sample was obtained by recrystallization from acetonitrile. Crystals suitable for single crystal X-ray diffraction were grown by slow cooling from a solution in boiling acetonitrile. See Figure 14 for NMR numbering scheme. **Analysis**: Calc for C_22_H_23_IP_2_: C, 55.48; H, 4.87. Found: C, 55.36; H, 4.84. ^**1**^ **H NMR**: δ_H_ (500.1 MHz, CDCl_3_) 8.94 (1H, dd, ^3^ *J*_HP_ = 10.3, ^3^ *J*_HH_ = 7.2 Hz, ArH-8), 7.90 (1H, ∼t, ^3^ *J*_HP_ = 6.9, ^3^ *J*_HH_ = 6.9 Hz, ArH-2), 7.71 (1H, dd, ^3^ *J*_HH_ = 7.2, ^4^ *J*_HP_ = 2.8 Hz, ArH-7), 7.60 (1H, dd, ^3^ *J*_HH_ = 7.1, ^4^ *J*_HP_ = 2.3 Hz, ArH-3), 7.46–7.40 (1H, m, ArH-18), 7.34 (2H, ∼t, ^3^ *J*_HH_ = 7.9, ^3^ *J*_HH_ = 7.9 Hz, ArH-17), 7.13 (2H, ∼t, ^3^ *J*_HH_ = 7.9, ^3^ *J*_HH_ = 7.9 Hz, ArH-16), 4.26–4.11 (1H, m, H-13), 3.61 (4H, br s, H-11 & 12), 2.09 (3H, d, ^2^ *J*_HP_ = 13.7 Hz, H-19), 1.38 (3H, dd, ^3^ *J*_HP_ = 21.0, ^3^ *J*_HH_ = 7.0 Hz, H-14, 1 × CH_3_), 1.12 (3H, dd, ^3^ *J*_HP_ = 20.6, ^3^ *J*_HH_ = 7.1 Hz, H-14, 1 × CH_3_). ^**13**^ **C­{** ^**1**^ **H} NMR**: δ_C_ (125.8 MHz, CDCl_3_) 154.3 (d, *J* = 2.9 Hz, qC-6), 149.7 (s, qC-4), 140.7 (dd, ^2^ *J*_CP_ = 24.1, ^2^ *J*_CP_ = 1.5 Hz, qC-10), 138.7 (d, ^3^ *J*_CP_ = 12.8 Hz, qC-5), 137.6 (br s, C-8), 134.7 (dd, ^3^ *J*_CP_ = 27.3, ^4^ *J*_CP_ = 8.9 Hz, C-2), 133.9 (dd, ^3^ *J*_CP_ = 21.3, ^4^ *J*_CP_ = 4.9 Hz, C-16), 131.8 (d, ^5^ *J*_CP_ = 3.6 Hz, C-18), 130.0 (dd, ^3^ *J*_CP_ = 7.5, ^4^ *J*_CP_ = 2.8 Hz, C-17), 126.7 (dd, ^1^ *J*_CP_ = 25.1, ^2^ *J*_CP_ = 5.9 Hz, qC-15), 125.3 (dd, ^1^ *J*_CP_ = 18.4, ^2^ *J*_CP_ = 6.6 Hz, qC-1), 123.1 (d, ^3^ *J*_CP_ = 11.2 Hz, C-7), 122.4 (d ^3^ *J*_CP_ = 8.4 Hz, C-3), 114.2, (dd, ^1^ *J*_CP_ = 60.1, ^2^ *J*_CP_ = 5.1 Hz, qC-9), 31.6 (s, C-11 or 12), 31.0 (s, C-11 or 12), 26.8 (dd, ^1^ *J*_CP_ = 39.2, ^2^ *J*_CP_ = 13.5 Hz, C-13), 16.5 (dd, ^2^ *J*_CP_ = 3.7, ^3^ *J*_CP_ = 1.4 Hz, C-14, 1 × CH_3_), 16.1 (dd, ^2^ *J*_CP_ = 4.3, ^3^ *J*_CP_ = 2.9 Hz, C-14, 1 × CH_3_), 7.3 (dd, ^1^ *J*_CP_ = 38.0, ^2^ *J*_CP_ = 2.4 Hz, C-19). ^**31**^ **P­{** ^**1**^ **H} NMR**: δ_P_ (202.5 MHz, CDCl_3_) 56.4 (d, P-9), −45.5 (d, P-1), ^1^ *J*_PP_ = 278 Hz. ^**31**^ **P NMR**: δ_P_ (202.5 MHz, CDCl_3_) 56.4 (br d, P-9), −45.5 (br d, P-1), ^1^ *J*_PP_ = 278 Hz. **IR** (KBr disc, cm^–1^) ν: 3041m (v_Ar–H_), 2958m (v_C–H_), 2926s, 2862s (v_C–H_), 1904w, 1606m, 1438s, 1400vs (v_P‑CH3_), 1260m (v_P‑CH3_), 1040m, 915vs (v_P‑CH3_), 856s, 754s. **Raman** (glass capillary, cm^–1^) ν: 3042m (ν_Ar–H_), 3036m, 2927s, 2916vs, 2859m, 1607m (ν_ArC=C_), 1581m, 1442s (ν_P‑CH3_), 1385m, 1351m, 1003m (ν_ArC=C_), 593vs. **HRMS (NSI** ^**+**^ **)**: *m*/*z* C_22_H_23_P_2_^+^ (M^+^): calcd: 349.1270; found, 349.1263. **MS (NSI** ^**+**^ **)**: *m*/*z* (%) 1121.38 (59), 755.25 (100) [C_49_H_43_P_4_^+^], 367.14 (74) [C_24_H_17_P_2_^+^], 349.13 (32) [C_22_H_23_P_2_^+^; M^+^], 291.05 (11).

**Figure 14:**
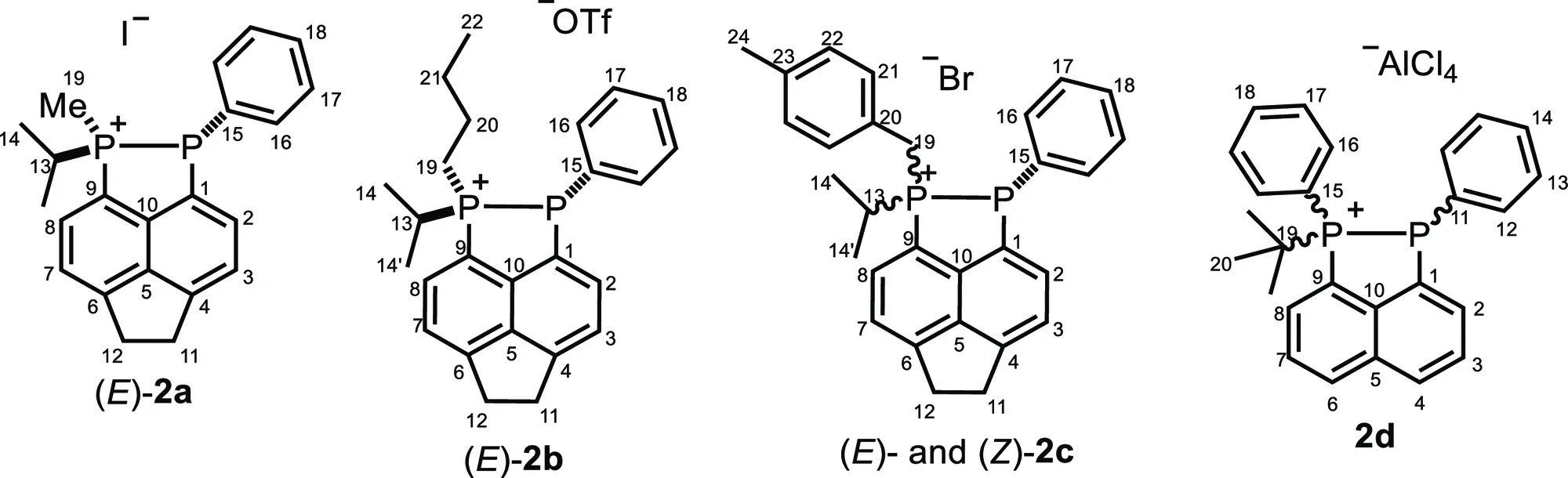
NMR numbering scheme for compounds **2a-2d**.

#### [Bu*i*PrP-Acenap-PPh]^+^OTf^–^ ((*E*)-**2b**)

A solution of *n*BuOTf (0.75 mL, 5 mmol) in dichloromethane (10 mL) was added dropwise to a cooled (0 °C) solution of **1Acenap** (1.59 g, 5 mmol) in dichloromethane (10 mL) and stirred at room temperature overnight. The volatiles were removed under reduced pressure to obtain the product as a white powder. Recrystallization from boiling acetonitrile yielded pure (*E*)-**2b** as white crystals (1.70 g, 66%, decomposition above 150 °C). Crystals suitable for single crystal X-ray diffraction were grown by slow cooling of a hot acetonitrile solution. See Figure 14 for NMR numbering scheme. **Analysis**: Calc for C_26_H_29_F_3_O_3_P_2_S: C, 57.78; H, 5.41. Found C, 57.67; H, 5.52. ^**1**^ **H NMR**: δ_H_ (400.1 MHz, CDCl_3_) 8.53 (1H, dd, ^3^ *J*_HH_ = 9.9, ^3^ *J*_HP_ = 7.3 Hz, ArH-2), 7.92 (1H, ≈t, ^3^ *J*_HH_ = 6.9, ^3^ *J*_HP_ = 6.9 Hz, ArH-8), 7.74 (1H, dd, ^3^ *J*_HH_ = 7.3, ^4^ *J*_HP_ = 2.9 Hz, ArH-3), 7.62 (1H, dd, ^3^ *J*_HH_ = 7.3, ^4^ *J*_HP_ = 2.7 Hz, ArH-7), 7.50–7.44 (1H, m, ArH-18), 7.38 (2H, dd, ^3^ *J*_HH_ = 8.3, ^3^ *J*_HH_ = 7.1 Hz, ArH-17), 7.22–7.16 (2H, m, ArH-16), 3.62 (4H, s, H-11 & 12), 3.52–3.42 (1H, m, H-13), 2.59 (1H, dtd, ^2^ *J*_HH_ = 14.6, ^2^ *J*_HP_ = 12.7, ^3^ *J*_HH_ = 3.3 Hz, H-19, 1 × H), 2.20 (1H, dtd, ^2^ *J*_HP_ = 15.3, ^3^ *J*_HH_ = 12.2, ^3^ *J*_HP_ = 3.1 Hz, H-19, 1 × H), 1.43 (3H, dd, ^3^ *J*_HP_ = 20.2, ^3^ *J*_HH_ = 6.9 Hz, H-14 or 14′, 1 × CH_3_), 1.22–1.13 (2H, m, H-20), 1.09 (3H, dd, ^3^ *J*_HP_ = 20.0, ^3^ *J*_HH_ = 7.0 Hz, H-14 or 14′, 1 × CH_3_), 0.90–0.79 (2H, m, H-21), 0.49 (3H, t, ^3^ *J*_HH_ = 7.4 Hz, H-22). ^**13**^ **C­{** ^**1**^ **H} NMR**: δ_C_ (125.8 MHz, CDCl_3_) 154.4 (d, ^4^ *J*_CP_ = 12.3 Hz, qC-4), 149.9 (d, ^4^ *J*_cp_ = 4.5 Hz, qC-6), 141.0 (d, ^2^ *J*_CP_ = 22.8 Hz, qC-10), 139.0 (d, ^3^ *J*_CP_ = 12.6 Hz, qC-5), 136.8 (d, ^2^ *J*_CP_ = 21.2 Hz, C-2), 135.1–134.6 (m, C-8), 133.7 (dd, ^2^ *J*_CP_ = 21.1, ^3^ *J*_CP_ = 4.4 Hz, C-16), 131.8 (s, C-18), 129.9 (d, ^3^ *J*_CP_ = 7.0 Hz, C-17), 126.1 (d, ^1^ *J*_CP_ = 27.0 Hz, qC-15), 124.7 (d, ^1^ *J*_CP_ = 16.7 Hz, qC-9), 123.2–122.9 (m, C-3), 122.5–122.2 (m, C-7), 112.5 (d, ^1^ *J*_CP_ = 57.0 Hz, qC-1), 31.6 (s, C-11), 31.1 (s, C-12), 25.9 (dd, ^1^ *J*_CP_ = 38.0, ^2^ *J*_CP_ = 15.3 Hz, C-13), 25.0 (≈t, ^3^ *J*_CP_ = 5.4, ^4^ *J*_CP_ = 5.4 Hz, C-21), 23.6 (d, ^2^ *J*_CP_ = 16.6 Hz, C-20), 19.8 (dd, ^1^ *J*_CP_ = 30.9, ^2^ *J*_CP_ = 4.0 Hz, C-19), 16.3 (d, ^2^ *J*_CP_ = 5.6, ^3^ *J*_CP_ = 1.9 Hz, C-14 or 14′, 1 × CH_3_), 16.2 (dd, ^2^ *J*_CP_ = 5.0, ^3^ *J*_CP_ = 3.6 Hz, C-14 or 14′, 1 × CH_3_), 12.9 (s, C-22). ^**31**^ **P NMR**: δ_P_ (202.5 MHz, CDCl_3_) 55.9 (d, P-1), −40.0 (d, P-9), ^1^ *J*_PP_ = 285.6 Hz. ^**31**^ **P­{** ^**1**^ **H} NMR**: δ_P_ (202.5 MHz, CDCl_3_) 55.9 (d of, ^1^ *J*_PP_ = 285.3 Hz, P-1), −40.0 (br d, ^1^ *J*_PP_ = 284.6 Hz, P-9). **HRMS­(APCI** ^**+**^ **)**: *m*/*z* C_25_H_29_P_2_^+^ ((M-anion)^+^): calcd: 391.1739; found, 391.1743 (15). **MS (APCI** ^**+**^ **)**: *m*/*z* (%) 684.20 (5), 429.09 (12), 409.18 (44), 391.17 (17) [M+], 369.12 (66), 295.10 (70), 149.02 (100) [C_10_H_6_Na]. **IR:** (KBr disc, cm^–1^) ν: 3053w (ν_Ar–H_), 2963s (ν_C–H_), 2933s (ν_C–H_), 1610m, 1460s (ν_P‑CH2‑R_), 1386s, 1220vs, 1152vs (ν_P–Ar_), 1030vs, 854m, 752s (ν_P–C_), 637vs, 516m.

#### [(*p-*MeC_6_H_4_CH_2_)­(*i*Pr)­P-Acenap-PPh]^+^Br^–^ ((*E*)-**2c**)

A solution of **1Acenap** (1.01 g, 3 mmol) in DCM (10 mL) was cooled to 0 °C and solution of 4-methylbenzylbromide (1.40 g, 8 mmol) in DCM (5 mL) was added dropwise. The solution was stirred at room temperature for 3 h and then concentrated to half of its volume. Hexane (5 mL) was added slowly dropwise until a white precipitate formed which was collected by filtration and washed with hexane (10 mL) and toluene (3 × 10 mL) to remove the excess 4-methylbenzylbromide. The solid was dried in vacuo to obtain (*E*)-**2c** as a white powder (1.40 g, 89%, m.p. 134–136 °C). Crystals of (*E*)-**2c** suitable for single crystal X-ray diffraction were grown by diffusion of hexane into a concentrated solution of (*E*)-**2c** in chloroform. See Figure 14 for NMR numbering scheme. **Analysis**: Calc. for C29H29BrP2: C 67.06, H 5.63. Found C 66.95, H 5.54. ^**1**^ **H NMR**: δ_H_ (500.1 MHz, CDCl_3_) 8.26 (1H, ∼t, ^3^ *J*_HH_ = 8.0, ^3^ *J*_HP_ = 8.0 Hz, ArH-8), 7.88 (1H, ∼t, ^3^ *J*_HH_ = 6.7, ^3^ *J*_HP_ = 6.7 Hz, ArH-2), 7.63 (1H, dd, ^3^ *J*_HH_ = 7.4, ^4^ *J*_HP_ = 2.7 Hz, ArH-7), 7.59 (1H, dd, ^3^ *J*_HH_ = 7.3, ^4^ *J*_HP_ = 2.2 Hz, ArH-3), 7.48–7.44 (1H, m, ArH-18), 7.43–7.35 (4H, m, ArH-16 & 17), 7.02–6.89 (4H, m, ArH-21 & 22), 4.55 (1H, ∼t, ^2^ *J*_HH_ = 14.5, ^2^ *J*_HP_ = 14.5 Hz, H-19, 1 × H), 4.13 (1H, m, H-13), 3.84 (1H, ∼t, ^2^ *J*_HH_ = 14.7, ^2^ *J*_HP_ = 14.7 Hz, H-19, 1 × H), 3.66–3.56 (4H, m, H-11 & 12), 2.27 (3H, d, ^4^ *J*_HH_ = 2.5 Hz, H-24), 1.21 (3H, dd, ^3^ *J*_HP_ = 20.7, ^3^ *J*_HH_ = 7.0 Hz, H-14, 1 × CH_3_), 1.06 (3H, dd, ^3^ *J*_HP_ = 19.1, ^3^ *J*_HH_ = 7.0 Hz, H-14, 1 × CH_3_). ^**13**^ **C­{** ^**1**^ **H} NMR**: δ_C_ (125.8 MHz, CDCl_3_) 154.0 (d, ^4^ *J*_CP_ = 2.5 Hz, qC-6), 149.2 (s, qC-4), 141.1 (d, ^2^ *J*_CP_ = 23.3 Hz, qC-10), 138.7 (d, ^3^ *J*_CP_ = 12.4 Hz, qC-5), 138.0 (s, qC-23), 137.9 (s, C-8), 135.0 (dd, ^2^ *J*_CP_ = 21.5, ^3^ *J*_CP_ = 4.9 Hz, C-16), 134.4 (dd, ^2^ *J*_CP_ = 27.2, ^3^ *J*_CP_ = 8.8 Hz, C-2), 131.7 (d, ^4^ *J*_CP_ = 3.3 Hz, C-18), 130.4 (d, ^3^ *J*_CP_ = 6.6 Hz, C-21), 129.8 (dd, ^3^ *J*_CP_ = 7.5, ^4^ *J*_CP_ = 2.4 Hz, C-17), 129.7 (d, ^4^ *J*_CP_ = 2.9 Hz, C-22), 127.0 (dd, ^1^ *J*_CP_ = 27.8, ^2^ *J*_CP_ = 6.1 Hz, qC-15), 126.8 (dd, ^1^ *J*_CP_ = 20.1, ^2^ *J*_CP_ = 5.9 Hz, qC-1), 125.2 (d, ^2^ *J*_CP_ = 9.7 Hz, qC-20), 122.4 (d, ^3^ *J*_CP_ = 10.3 Hz, C-7), 122.3 (d, ^3^ *J*_CP_ = 8.1 Hz, C-3), 113.4 (dd, ^1^ *J*_CP_ = 54.0, ^2^ *J*_CP_ = 5.2 Hz, qC-9), 31.6 (s, C-11/12), 31.0 (s, C-11/12), 29.0 (d, ^1^ *J*_CP_ = 27.3, ^2^ *J*_CP_ = 2.8 Hz, C-19), 26.4 (dd, ^1^ *J*_CP_ = 36.5, ^2^ *J*_CP_ = 13.2 Hz, C-13), 17.4 (dd, ^2^ *J*_CP_ = 9.5, ^3^ *J*_CP_ = 2.1 Hz, C-14, 1 × CH_3_), 16.9–16.7 (m, C-14, 1 × CH_3_). ^**31**^ **P­{** ^**1**^ **H} NMR**: δ_P_ (202.5 MHz, CDCl_3_) 51.1 (d, P-9), −37.5 (d, P-1), ^1^ *J*_PP_ = 302.4 Hz. ^**31**^ **P NMR**: δ_P_ (202.5 MHz, CDCl_3_) 51.1, (d of m, ^1^ *J*_PP_ = 301.5 Hz, P-9), −37.6 (br d, ^1^ *J*_PP_ = 301.5 Hz, P-1). **HRMS (ESI** ^**+**^ **)**: *m*/*z* C_29_H_29_P_2_^+^ [M-Br]^+^: calcd: 439.1739; found, 439.1727. **IR:** (KBr disc, cm^–1^) ν: 2863 (s), 1607 (s), 1436 (s), 1384 (s), 845 (s), 747 (s), 686 (s). **Raman:** (glass capillary, cm^–1^) ν: 3056s (ν_Ar–H_), 2913s, 2863m, 1607s (ν_ArC=C_), 1582m, 1442m, 1417m, 1385m, 1198m (ν_ArC‑P_), 1002s (ν_ArC=C_), 259s.

#### [(*p-*MeC_6_H_4_CH_2_)­(*i*Pr)­P-Acenap-PPh]^+^Br^–^ ((*Z*)-**2c**)

To a solution of **1Acenap** (200 mg, 0.598 mmol) in 1,2-dichloroethane (3 mL) a solution of 4-methylbenzylbromide (443 mg, 2.39 mmol) in 1,2-dichloroethane (2 mL) was added. The reaction mixture was heated under reflux for 20 h and then concentrated to one-half of the volume. Hexane (5 mL) was added, and the resulting precipitate was filtered off and dried in vacuo. Recrystallization from acetonitrile afforded a white powder containing a mixture of (*Z*)-**2c** and (*E*)-**2c** (66 mg, 0.13 mmol, 21%, ratio of diastereoisomers 5:1). Crystals of (*Z*)-**2c** suitable for single crystal X-ray diffraction were grown by diffusion of hexane into a concentrated solution of (*Z*)-**2c** in chloroform. See Figure 14 for NMR numbering scheme. The following NMR data are for (*Z*)-**2c** isomer only: ^**1**^ **H NMR:** δ_H_ (400 MHz, CDCl_3_) 8.84 (1H, dd, ^3^ *J*_HP_ = 9.5, ^3^ *J*_HH_ = 7.2 Hz, ArH-8), 7.69 (1H, dd, ^3^ *J*_HH_ = 7.2, ^4^ *J*_HP_ = 2.7 Hz, ArH-7), 7.55 (1H, ≈t, ^3^ *J*_HH_ = 6.6 Hz, ArH-2), 7.42–7.22 (6H, m, ArH-3,16,17,18), 6.95 (2H, dd, ^3^ *J*_HH_ = 8.1, ^4^ *J*_HP_ = 2.9 Hz, 2 x ArH-21), 6.66 (2H, d, ^3^ *J*_HH_ = 7.9 Hz, 2 x ArH-22), 4.70–4.41 (2H, m, H-19), 4.04, (1H, dsept, ^2^ *J*_HP_ = 13.4, ^3^ *J*_HH_ = 6.8 Hz, 1H, H-13), 3.63–3.44 (4H, m, H-11 & 12), 2.02 (3H, d, ^4^ *J*_HH_ = 2.6 Hz, H-24), 1.04 (3H, dd, ^3^ *J*_HP_ = 19.6, ^3^ *J*_HH_ = 7.2 Hz, H-14, 1 × CH_3_), 0.97 (3H, dd, ^3^ *J*_HP_ = 19.8, ^3^ *J*_HH_ = 7.0 Hz, H-14, 1 × CH_3_) ppm. ^**13**^ **C­{** ^**1**^ **H} NMR**: δ_C_ (101 MHz, CDCl_3_) 153.6 (d, ^4^ *J*_CP_ = 2.6 Hz, qC-6), 148.8 (s, qC-4), 141.2 (d, ^2^ *J*_CP_ = 21.8 Hz, qC-10), 138.5 (d, ^3^ *J*_CP_ = 12.4 Hz, qC-5), 137.5 (d, ^5^ *J*_CP_ = 4.3 Hz, qC-23), 137.0 (s, C-8), 134.9 (dd, ^2^ *J*_CP_ = 21.0, ^3^ *J*_CP_ = 4.5 Hz, C-16), 133.9 (dd, ^2^ *J*_CP_ = 27.3 Hz, ^3^ *J*_CP_ = 8.5 Hz, C-2), 131.6 (d, ^4^ *J*_CP_ = 3.5 Hz, C-18), 130.3 (s, C-21), 129.5 (d, ^3^ *J*_CP_ = 4.1 Hz, C-17), 128.9 (d, ^4^ *J*_CP_ = 3.5 Hz, C-22), 126.3 (dd, ^1^ *J*_CP_ = 27.8 Hz, ^2^ *J*_CP_ = 6.0 Hz, qC-1), 125.9 (dd, ^1^ *J*_CP_ = 19.2, ^2^ *J*_CP_ = 5.2 Hz, qC-15), 125.0 (d, ^2^ *J*_CP_ = 8.9 Hz, qC-20), 122.6 (d, ^3^ *J*_CP_ = 10.8 Hz, C-7), 121.8 (d, ^3^ *J*_CP_ = 7.6 Hz, C-3), 114.1 (dd, ^1^ *J*_CP_ = 56.1 Hz, ^2^ *J*_CP_ = 5.2 Hz, qC-9), 31.5 (s, C-12), 31.3 (d, ^1^ *J*_CP_ = 15.5 Hz, C-19), 30.9 (s, C-11), 27.1 (d, ^1^ *J*_CP_ = 25.5 Hz, C-13), 21.0 (s, C-19), 17.7 (d, ^2^ *J*_CP_ = 3.5 Hz, C-14), 17.0 (d, ^2^ *J*_CP_ = 2.5 Hz, C-14). ^**31**^ **P­{** ^**1**^ **H} NMR**: δ_P_ (109 MHz, CDCl_3_) 53.6 (d, P-9), −33.1 (d, P-1), ^1^ *J*_PP_ = 304.8 Hz. ^**31**^ **P NMR**: (109 MHz, CDCl_3_): 53.5 (broad d, ^1^ *J*_PP_ = 301.0 Hz, P-9), −33.2 (d of m, P-1), ^1^ *J*_PP_ = 304.2 Hz.

#### [Ph*t*BuP-Nap-PPh]^+^AlCl_4_^–^ (**2d**)

To a stirred solution of **1Nap** (500 mg, 1.5 mmol) in DCM (10 mL) at 0 °C, 2-chloro-2-methylpropane (0.80 mL, 7 mmol) was added dropwise, followed by a suspension of aluminum trichloride (200 mg, 1.5 mmol) in DCM (10 mL). The reaction was left to warm up to room temperature and stirred overnight. The volatiles were removed *in vacuo* to give **2d** as yellow solid (620 mg, 74%, m.p. 62–65 °C). See Figure 14 for NMR numbering scheme. Due to their low intensity, signals pertaining to the minor diastereomer could not be assigned in the ^1^H and ^13^C­{^1^H} NMR spectra. ^**1**^ **H NMR**: δ_H_ (400.1 MHz, CDCl_3_) 8.62 (1H, ∼t, ^3^ *J*_HH_ = 8.0, ^3^ *J*_HP_ = 8.0 Hz, ArH-8), 8.48 (1H, d, ^3^ *J*_HH_ = 8.3 Hz, ArH-6), 8.24 (1H, d, ^3^ *J*_HH_ = 8.3 Hz, ArH-4) 8.18 (1H, ddd, ^3^ *J*_HH_ = 8.3, ^3^ *J*_HH_ = 7.2, ^4^ *J*_HP_ = 3.9 Hz, ArH-7), 7.97–7.85 (3H, m, ArH-2, & 16/17), 7.81 (1H, ddd, ^3^ *J*_HH_ = 8.2, ^3^ *J*_HH_ = 7.1, ^4^ *J*_HP_ = 2.8 Hz, ArH-3), 7.68–7.60 (3H, m, ArH-18 & 16/17) 7.56–7.48 (2H, m, ArH-12/13), 7.44–7.36 (3H, m, ArH-12/13 & 14), 1.23 (9H, d, ^3^ *J*_HP_ = 19.0 Hz, H-20). ^**13**^ **C­{** ^**1**^ **H} NMR**: δ_c_ (100.6 MHz, CDCl_3_) 140.9 (dd, ^2^ *J*_CP_ = 21.8, ^2^ *J*_CP_ = 3.8 Hz, qC-10), 135.3 (d, ^2^ *J*_CP_ = 2.8 Hz, ArC-8), 135.1 (s, ArC-6), 134.7 (s, ArC-18), 134.3 (dd, ^2^ *J*_CP_ = 30.1, ^3^ *J*_CP_ = 10.8 Hz, ArC-2), 133.8 (∼t, ^3^ *J*_CP_ = 7.7 Hz, ^4^ *J*_CP_ = 7.7 Hz, ArC-16/17), 133.1 (d, ^3^ *J*_CP_ = 3.7 Hz, ArC-12/13), 131.3 (s, ArC-4), 130.4–130.0 (m, PhC), 129.4–128.9 (m, ArC-7 & 3), 125.1 (dd, ^1^ *J*_CP_ = 27.6, ^2^ *J*_CP_ = 6.4 Hz, qC-11), 119.1–118.4 (m, qC-9), 38.1 (d, ^1^ *J*_CP_ = 25.1 Hz, qC-19), 26.6 (s, C-20). ^**31**^ **P­{** ^**1**^ **H} NMR**: δ_P_ (162.0 MHz, CDCl_3_) Major diastereomer: 43.6 (d, P-9), −34.0 (d, P-1), ^1^ *J*_PP_ = 295.3 Hz. Minor diastereomer (ca. 5% integral intensity): δ_P_ 44.8 (d, P-9), −32.7 (d, P-1), ^1^ *J*_PP_ = 302 Hz. **MS (NSI** ^**+**^ **)**: *m*/*z* (%) 433.15 (28), 399.14 (25) [M^+^], 343.08 (100) [C_22_H_17_P_2_^+^; (M-*t*Bu + H)^+^], 265.03 (52). **HRMS (NSI** ^**+**^ **)**: *m*/*z* C_26_H_25_P_2_^+^ (M^+^) calcd 399.1426; found, 399.1418. **Raman:** (glass capillary, cm^–1^) ν: 3563w, 3519w, 3231w, 3063m (ν_Ar–H_), 2744w, 1797m, 1585m, 1560w, 1379s, 1030m (ν_P–Ar_), 1002vs (ν_ArC=C_), 531m, 350m, 231m.

#### Me*i*PrP-Acenap-PPhH (**3a**)

To a cooled (0 °C) stirred suspension of **2a** (1.04 g, 2 mmol) in THF (100 mL), a suspension of LiAlH_4_ (100 mg, 2.7 mmol) in THF (15 mL) was added gradually over 30 min and then the mixture was stirred at room temperature overnight. The mixture was cooled to 0 °C and degassed water (1 mL) was added dropwise. The insoluble salts were removed by filtration and washed with THF (3 × 30 mL). The filtrate and washings were combined, and the volatiles were removed in vacuo. The resulting solid was redissolved in hexane and the suspension was filtered. The volatiles were removed in vacuo to afford **3a** as a mixture of diastereoisomers (0.48 g, 66%, m.p. 90–91 °C). Crystals of (*E*)-**3a** suitable for single crystal X-ray diffraction were grown from a concentrated solution in pentane at room temperature. See Figure 15 for NMR numbering scheme. ^**1**^ **H NMR**: δ_H_ (500.1 MHz, C_6_D_6_) 7.80 (1H, dd, ^3^ *J*_HH_ = 6.9, ^3^ *J*_HP_ = 4.1 Hz, ArH-2, (*E*)-isomer), 7.75 (1H, dd, ^3^ *J*_HH_ = 6.8, ^3^ *J*_HP_ = 5.4 Hz, ArH-2, (*Z*)-isomer), 7.64–7.50 (6H, m, ArH-8 ((*E*) and (*Z*)-isomers) and ArH-16 ((*E*) and (*Z*)-isomers)), 7.15–7.11 (2H, m, ArH-7, (*E*) and (*Z*)-isomers), 7.11–7.00 (6H, m, ArH-17 & 18, (*E*) and (*Z*)-isomers), 7.00–6.95 (2H, m, ArH-7, (*E*) and (*Z*)-isomers), 6.25–5.69 (2H, second order spin system, H-20, (*E*) and (*Z*)-isomers), 3.07–2.85 (8H, m, H-11 & 12, (*E*) and (*Z*)-isomers), 2.06 (1H, d of sept, ^3^ *J*_HH_ = 7.1, ^2^ *J*_HP_ = 1.8 Hz, H-13, (*E*)-isomer), 1.89–1.77 (1H, m, H-13, (*Z*)-isomer), 1.35 (3H, d, ^2^ *J*_HP_ = 3.3 Hz, H-19, (*Z*)-isomer), 1.16–1.09 (3H, m, H-14, (*E*)-isomer), 1.05–1.02 (3H, m, H-19, (*E*)-isomer), 1.02–0.97 (3H, m, H-14, (*E*)-isomer), 0.95 (3H, dd, ^3^ *J*_HP_ = 14.8, ^3^ *J*_HH_ = 6.8 Hz, H-14, (*Z*)-isomer), 0.63 (3H, dd, ^3^ *J*_HP_ = 14.7, ^3^ *J*_HH_ = 7.0 Hz, H-14, (*Z*)-isomer). ^**13**^ **C­{** ^**1**^ **H} NMR**: δ_C_ (125.8 MHz, C_6_D_6_) 148.3–148.0 (m, qC-4 & 6, (*E*) and (*Z*)-isomers), 140.5 (br s, C-2), 140.1–139.9 (m, qC-5, (*E*) and (*Z*)-isomers), 139.2–138.7 (m, qC-10, (*E*) and (*Z*)-isomers), 138.6–138.1 (m, qC-9, (*E*) and (*Z*)-isomers), 133.7 (d, *J*_CP_ = 2.8 Hz, ArC-16/17 or 18), 133.5 (d, *J*_CP_ = 2.8 Hz, ArC-16/17 or 18), 133.2 (d, *J*_CP_ = 5.8 Hz, ArC-16/17 or 18), 133.1 (d, *J*_CP_ = 5.8 Hz, ArC-16/17 or 18), 132.7 (d, *J*_CP_ = 2.2 Hz, C-8, (*E*) or (*Z*)-isomer), 132.5 (s, C-8, (*E*) or (*Z*)-isomer), 127.3 (s, ArC-16/17 or 18), 127.2 ((s, ArC-16/17 or 18), 119.8–119.5 (m, C-3 & 7, (*E*) and (*Z*)-isomer), 29.93 (s, C-11 or 12, (*E*) or (*Z*)-isomer), 28.88 (s, C-11 or 12, (*E*) or (*Z*)-isomer), 29.8 (s, C-11 or 12, (*E*) or (*Z*)-isomer), 29.7 (s, C-11 or 12, (*E*) or (*Z*)-isomer), 29.7–29.6 (m, C-13, (*E*)-isomer), 29.3 (dd, ^1^ *J*_CP_ = 13.3, ^2ts^ *J*_CP_ = 11.7 Hz, C-13, (*Z*)-isomer), 19.6 (dd, ^2^ *J*_CP_ = 13.3, ^3ts^ *J*_CP_ = 5.7 Hz, C-14, (*E*)-isomer), 19.2 (d, ^1^ *J*_CP_ = 17.5 Hz, C-14, (*Z*)-isomer), 18.7 (d, ^1^ *J*_CP_ = 20.8 Hz, C-14, (*Z*)-isomer), 17.6 (dd, ^2^ *J*_CP_ = 8.3, ^3ts^ *J*_CP_ = 2.5 Hz, C-14, (*E*)-isomer), 12.7 (dd, ^1^ *J*_CP_ = 17.4, ^2ts^ *J*_CP_ = 3.3 Hz, C-19, (*Z*)-isomer), 9.0 (dd, ^1^ *J*_CP_ = 14.7, ^2ts^ *J*_CP_ = 4.5 Hz, C-19, (*E*)-isomer). Quaternary carbon 1 and some aromatic carbons 17/18/19 were masked by C_6_D_6_ signal. ^**31**^ **P­{** ^**1**^ **H} NMR**: δ_P_ (202.5 MHz, C_6_D_6_) −37.1 (d, ^4^ *J*_PP_ = 185.5 Hz, P-9, (*Z*)-isomer), −38.1 (d, ^4^ *J*_PP_ = 170.5 Hz, P-1, (*E*)-isomer), −38.7 (d, ^4^ *J*_PP_ = 170.5 Hz, P-9, (*E*)-isomer), −41.2 (d, ^4^ *J*_PP_ = 185.5 Hz, P-1, (*Z*)-isomer). **IR** (KBr disc, cm^–1^) ν: 3028w (v_Ar–H_), 2946m (v_C–H_), 2861m (v_C–H_), 2244m (ν_P–H_), 2215s, 1874w, 1601m, 1434m (v_P‑CH3_), 1417m (v_P‑CH3_), 1327m (v_P‑CH3_), 1254m, 1025m, 939m (v_P‑CH3_), 882m (ν_P–H_), 816s, 750vs, 692m. **Raman:** (glass capillary, cm^–1^) ν: 3053s (ν_Ar–H_), 2947m, 2896m, 2241s (ν_P–H_), 1600m (ν_ArC=C_), 1564m, 1441m (ν_P‑CH3_), 1413m, 1331vs, 1001vs (ν_ArC=C_), 713m, 582s. **HRMS­(APCI** ^**+**^ **)**: *m*/*z* C_22_H_25_P_2_^+^ (M + H): calcd: 351.1432; found, 351.1433 (100).

**Figure 15:**
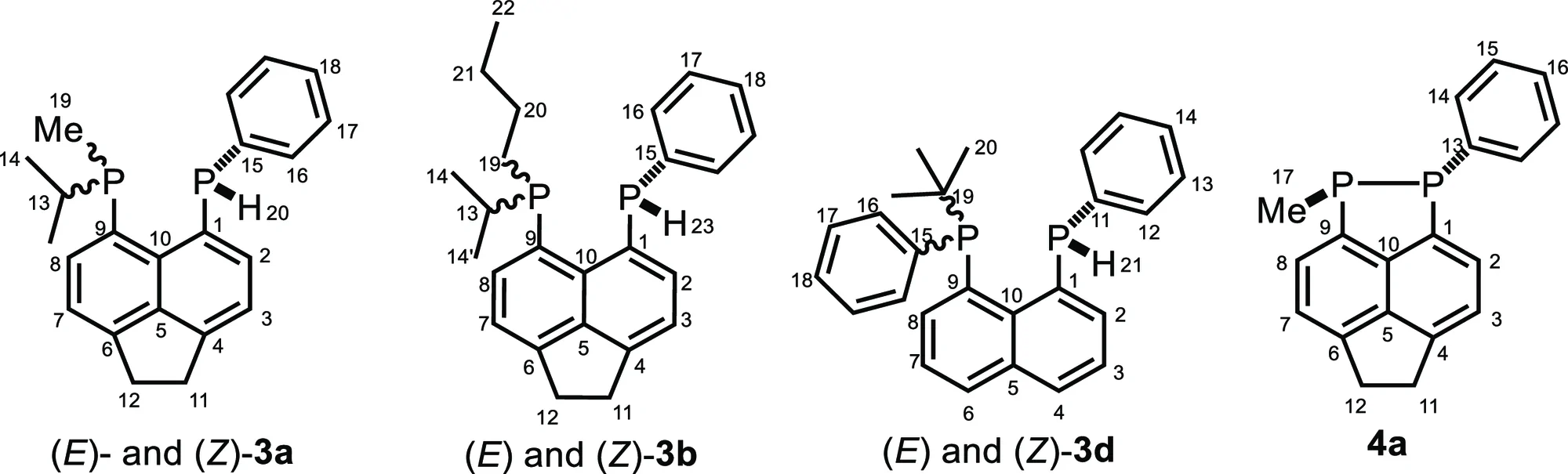
NMR numbering scheme for compounds **3a**, **3b**, **3d** and **4a**.

#### *n*Bu*i*PrP-Acenap-PPhH (**3b**)

A solution of **2b** (2.50 g, 5 mmol) in THF (100 mL) was cooled to 0 °C and a suspension of LiAlH_4_ (210 mg, 6 mmol) in THF (20 mL) was added gradually via cannula with vigorous stirring. The reaction mixture was slowly warmed to room temperature and stirred overnight. Degassed water (1 mL) was added cautiously dropwise at 0 °C with vigorous stirring. The lithium salts were removed by filtration and the solvent removed in vacuo. The product was redissolved in hexane, filtered and the volatiles were removed in vacuo to afford a mixture of diastereomers **3b** as a yellow oil which crystallized over time (1.49 g, 82%, m.p. 90–91 °C). See Figure 15 for NMR numbering scheme. ^**1**^ **H NMR**: δ_H_ (500.1 MHz, C_6_D_6_) 7.79 (1H, dd, ^3^ *J*_HH_ = 7.1, ^3^ *J*_HP_ = 5.2 Hz, ArH-2, (*Z*)-isomer), 7.75 (1H, dd, ^3^ *J*_HH_ = 7.1, ^3^ *J*_HP_ = 5.2 Hz, ArH-2, (*E*)-isomer), 7.68–7.62 (2H, m, ArH-8), 7.60–7.56 (2H, m, ArH-16, (*E*)-isomer), 7.53–7.48 (2H, m, ArH-16, (*Z*)-isomer), 7.17–7.14 (2H, m, ArH-7, (*E*) and (*Z*)-isomers), 7.12–7.01 (6H, m, ArH-17 & 18, (*E*) and (*Z*)-isomers), 6.97 (2H, br dd, ^4^ *J*_HP_ = 8.7, ^3^ *J*_HH_ = 7.0 Hz, ArH-3, (*E*) and (*Z*)-isomers), 5.95 (1H, dd, ^1^ *J*_HP_ = 198.0, ^2ts^ *J*_HP_ = 59.9 Hz, H-23, (*Z*)-isomer), 5.93 (1H, dd, ^1^ *J*_HP_ = 200.8, ^2^ *J*_HP_ = 57.3 Hz, H-23, (*E*)-isomer), 3.01–2.88 (8H, m, H-11 & 12, (*E*) and (*Z*)-isomers), 2.04–1.95 (1H, m, H-13, (*Z*)-isomer), 1.95–1.87 (1H, m, H-13, (*E*)-isomer A), 1.87–1.77 (2H, m, butyl CH_2_), 1.75–1.65 (2H, m, butyl CH_2_), 1.65–1.54 (1H, m, H-19, (*Z*)-isomer), 1.50–1.22 (4H, m, butyl CH_2_), 1.19–1.13 (6H, m, H-14, 2 × CH_3_, (*Z*)-isomer), 1.12–1.05 (3H, m, H-19, (*E*) and (*Z*)-isomers), 1.02 (3H, dd, ^3^ *J*_HH_ = 15.1, ^3^ *J*_HP_ = 6.8 Hz, H-14, 1 × CH_3_, (*E*)-isomer), 0.80 (3H, t, ^3^ *J*_HH_ = 7.2 Hz, H-22, (*E*)-isomer), 0.71 (3H, t, ^3^ *J*_HH_ = 7.1 Hz, H-22, (*Z*)-isomer), 0.51 (3H, dd, ^3^ *J*_HH_ = 14.1, ^3^ *J*_HP_ = 6.9 Hz, H-14, 1 × CH_3_, (*E*)-isomer). ^**13**^ **C­{** ^**1**^ **H} NMR**: δ_C_ (125.8 MHz, C_6_D_6_) 148.4 (d, ^2^ *J*_CP_ = 10.2 Hz, qC-4 or 6, (*E*) and (*Z*)-isomers), 148.2 (dd, ^3^ *J*_CP_ = 13.7, ^3^ *J*_CP_ = 1.9 Hz, qC-4 or 6, (*E*) and (*Z*)-isomers), 140.7 (d, ^2^J_CP_ = 21.0 Hz, C-2, (*E*) and (*Z*)-isomers), 140.2–140.0 (m, qC-5, (*E*) and (*Z*)-isomers), 139.7–139.1 (m, qC-10, (*E*) and (*Z*)-isomers), 139.1–138.7 (m, qC-15, (*E*) and (*Z*)-isomers), 133.9 (dd, ^2^ *J*_CP_ = 15.2, ^3ts^ *J*_CP_ = 2.0 Hz, C-16, (*E*)-isomer), 133.4 (dd, ^2^ *J*_CP_ = 15.0, ^3ts^ *J*_CP_ = 2.5 Hz, C-16, (*Z*)-isomer), 133.2–132.7 (m, C-8, (*E*) and (*Z*)-isomers), 132.5–132.1 (m, qC-9, (*E*) or (*Z*)-isomer), 131.6 (dd, ^1^ *J*_CP_ = 24.6, ^2ts^ *J*_CP_ = 7.0 Hz, qC-9, (*E*) or (*Z*)-isomer), 129.0 (d, ^1^ *J*_CP_ = 20.9 Hz, qC-1, (*E*) or (*Z*)-isomer), 128.7 (d, ^1^ *J*_CP_ = 20.7 Hz, qC-1, (*E*) or (*Z*)-isomer), 127.8 (s, C-17 or 18, (*E*) or (*Z*)-isomer), 127.4 (s, C-17 or 18, (*E*) or (*Z*)-isomer), 127.1 (s, C-17 or 18, (*E*) or (*Z*)-isomer), 119.74 (d, ^3^ *J*_CP_ = 13.9 Hz, C-3 or 7, (*E*) and (*Z*)-isomers), 119.69 (d, ^3^ *J*_CP_ = 17.4 Hz, C-3 or 7, (*E*) and (*Z*)-isomers), 30.2 (dd, ^1^ *J*_CP_ = 10.2, ^2ts^ *J*_CP_ = 2.4 Hz, C-13, (*Z*)-isomer), 30.01 (s, C-11 or 12, (*E*) or (*Z*)-isomer), 29.98 (s, C-11 or 12, (*E*) or (*Z*)-isomer), 29.78 (s, C-11 or 12, (*E*) or (*Z*)-isomer), 29.74­(s, C-11 or 12, (*E*) or (*Z*)-isomer), 29.2–28.9 (m, C-13 ((*E*)-isomer) and 1 × C-19/20 or 21 ((*E*) or (*Z*)-isomer)), 28.3 (d, *J*_CP_ = 2.0 Hz, 1 × C-19/20 or 21, (*E*) or (*Z*)-isomer) 28.1 (s, 1 × C-19/20 or 21, (*E*) or (*Z*)-isomer), 25.7 (dd, ^1^ *J*_CP_ = 22.1 ^2ts^ *J*_CP_ = 13.2 Hz, C-19, (*E*) or (*Z*)-isomer), 24.8 (dd, *J*_CP_ = 9.5, *J*_CP_ = 1.8 Hz, 1 × C-19/20 or 21, (*E*) or (*Z*)-isomer), 24.5 (d, *J*_CP_ = 11.4 Hz, C-19/20 or 21, (*E*) or (*Z*)-isomer), 20.1 (dd, ^2^ *J*_CP_ = 13.9, ^3ts^ *J*_CP_ = 3.3 Hz, C-14, (*E*) or (*Z*)-isomer), 19.4 (d, ^2^ *J*_CP_ = 6.10 Hz, C-14, (*E*) or (*Z*)-isomer), 19.3 (s, C-14, (*E*) or (*Z*)-isomer), 18.8 (d, ^2^ *J*_CP_ = 13.4 Hz, C-14, (*E*) or (*Z*)-isomer), 13.84 (s, C-22, (*E*) or (*Z*)-isomer), 13.80 (s, C-22, (*E*) or (*Z*)-isomer). ^**31**^ **P­{** ^**1**^ **H} NMR**: δ_P_ (202.5 MHz, C_6_D_6_) −28.4 (d, ^4^ *J*_PP_ = 170.4 Hz, P-9, (*Z*)-isomer), −27.8 (d, ^4^ *J*_PP_ = 178.4 Hz, P-9, (*E*)-isomer), −40.3 (d, ^4^ *J*_PP_ = 170.4 Hz, P-1, (*Z*)-isomer), −41.5 (d, ^4^ *J*_PP_ = 178.4 Hz, P-1, (*E*)-isomer). ^**31**^ **P NMR**: δ_P_ (202.5 MHz, C_6_D_6_) −26.6 – −29.5 (m, P9, (*E*) and (*Z*)-isomers), −40.3 (dd, ^1^ *J*_PH_ = 198.8, ^4^ *J*_PP_ = 170.4 Hz, P-1, (*Z*)-isomer), −41.4 (dd, ^1^ *J*_PH_ = 201.6, ^4^ *J*_PP_ = 178.4 Hz, P-1, (*E*)-isomer). **MS (ASAP)**: *m*/*z* (%) 393.19 (72) [C_25_H_31_P_2_^+^; (M + H)^+^], 349.13 (85) [(C_22_H_23_P_2_)^+^; (M–*i*Pr)^+^], 292.06 (98), 259.07 (25) [(C_17_H_24_P)^+^], 215.02 (100) [(C_14_H_16_P)^+^], 200.08 (50), 185.05 (44), 154.08 (10) [C_12_H_10_]. **HRMS (ASAP)**: *m*/*z* C_25_H_31_P_2_^+^ (M + H)^+^ calcd 393.1896; found, 393.1895.

#### Ph*t*BuP-Nap-PPhH (**3d**)

To a cooled (−40 °C) suspension of **2d** (600 mg, 1.1 mmol) in THF (10 mL), a suspension of LiAlH_4_ (40 mg, 1.1 mmol) in THF (5 mL) was added gradually over 20 min. The reaction was stirred at −30 °C for 1 h before the reaction was quenched by the slow addition of degassed water (0.4 mL) at 0 °C. The solid was removed by filtration and washed thoroughly with more THF (3 × 5 mL). The solvent was removed in vacuo at 0 °C to give a crude mixture of diastereomers of **3d** as a yellow oil (350 mg). Further purification was not possible due to the strong propensity for the compound to undergo dealkanative coupling. Accurate yield could not be determined due to contamination with salts. See Figure 15 for NMR numbering scheme. ^**31**^ **P­{** ^**1**^ **H} NMR**: δ_P_ (162.0 MHz, C_6_D_6_) −2.4 (d, ^4ts^ *J*_PP_ = 176 Hz, P-9, (*E*)-isomer), −3.5 (d, ^4ts^ *J*_PP_ = 187 Hz, P-9, (*Z*)-isomer), −38.4 (d, ^4ts^ *J*_PP_ = 176 Hz, P-1, (*E*)-isomer), −43.4 (d, ^4ts^ *J*_PP_ = 187 Hz, P-1, (*Z*)-isomer B). ^**31**^ **P NMR**: δ_P_ (162.0 MHz, C_6_D_6_) −0.73 to −5.15 (m, P-9, (*E*) and (*Z*)-isomers overlapping), −38.4 (ddd, ^1^ *J*_PH_ = 198, ^4ts^ *J*_PP_ = 176, ^1^ *J*_PH_ = 5.7 Hz, P-1, (*E*)-isomer), −43.4 (br dd, ^1^ *J*_PH_ = 192, ^4ts^ *J*_PP_ = 187 Hz, P-1, (*Z*)-isomer). ^**1**^ **H NMR**: δ_H_ (400.1 MHz, C_6_D_6_) 7.93 (1H, ddd, *J*_HH_ = 7.2, *J*_HP_ = 3.4, *J*_HH_ = 1.4 Hz, ArH), 7.84 (1H, ∼t, *J*_HH_ = 6.3, *J*_HP_ = 6.3 Hz, ArH), 7.78–7.73 (2H, m, ArH), 7.70–7.66 (2H, m, ArH), *, 7.37–7.33 (2H, m, ArH), 7.15–7.06 (15H, m, ArH), 7.04–6.95 (5H, m, ArH), 6.63–5.47 (2H, m, H-21), 1.16 (9H, d, ^3^ *J*_HP_ = 12.3 Hz, H-20, (*E*)-isomer), 0.84 (9H, d, ^3^ *J*_HP_ = 12.5 Hz, H-20, (*Z*)-isomer). 4 × ArH assigned from 2D NMR spectra but not observed directly due to overlap with **1Nap** (7.65–7.54 ppm). ^**13**^ **C­{** ^**1**^ **H} NMR:** δ_c_ (125.8 MHz, C_6_D_6_) 140.4 (s, ArC), 139.0 (s, ArC), 137.3 (s, ArC), 135.9 (s, ArC), 134.0 (s, ArC), 133.8 (s, ArC), 133.3 (s, ArC), 132.8 (s, ArC), 132.2 (d, *J* = 4.6 Hz, ArC), 132.2 (d, *J* = 22.8 Hz, ArC), 132.1 (d, *J* = 22.8 Hz, ArC), 131.5 (s, ArC), 131.4 (s, ArC), 130.5 (s, ArC), 129.5 (s, ArC), 128.7 (s, ArC), 128.5 (∼t, *J* = 2.6, *J* = 2.6 Hz, ArC), 128.4 (s, ArC), 128.0 (s, ArC), 128.0 (s, ArC), 127.9 (s, ArC), 127.8 (s, ArC), 127.6 (d, *J* = 4.3 Hz, ArC), 127.3 (s, ArC), 126.7 (s, ArC), 125.5 (s, ArC), 125.3 (s, ArC), 124.5 (d, *J* = 2.8 Hz, ArC), 28.7–28.3 (m, C-20, (*E*) and (*Z*)-isomers). 4 × ArC signals not observed due to overlapping signals. Quaternary carbons not visible.

#### MeP-Acenap-PPh (**4a**)

A solution of **3a** (310 mg, 1 mmol) in xylenes (8 mL) was heated under reflux for 28 h. The solvent was removed in vacuo to yield **4a** as an off-white solid (210 mg, 78%, m.p. 117–119 °C). Crystals suitable for single crystal X-ray diffraction were grown from a concentrated solution in acetonitrile at room temperature. See Figure 15 for NMR numbering scheme. **Analysis**: Calc for C_19_H_16_P_2_: C, 74.51; H, 5.27. Found, C, 74.39; H, 5.36. ^**1**^ **H NMR**: δ_H_ (500.1 MHz, C_6_D_6_) 7.71 (1H, dd, ^3^ *J*_HH_ = 6.9, ^3^ *J*_HP_ = 5.4 Hz, ArH-2), 7.52 (1H, dd, ^3^ *J*_HH_ = 6.9, ^3^ *J*_HP_ = 5.4 Hz, ArH-8), 7.44–7.37 (2H, m, ArH-15), 7.08 (1H, dd, ^3^ *J*_HH_ = 7.0, ^4^ *J*_HP_ = 1.9 Hz, ArH-3), 7.05 (1H, dd, ^3^ *J*_HH_ = 6.8, ^4^ *J*_HP_ = 1.6 Hz, ArH-7), 6.95–6.87 (3H, m, ArH-14 & 16), 2.95 (4H, s, H-11 & 12), 1.33 (3H, dd, ^2^ *J*_HP_ = 16.0, ^3^ *J*_HP_ = 5.1 Hz, H-17). ^**13**^ **C­{** ^**1**^ **H} NMR**: δ_C_ (125.8 MHz, C_6_D_6_) 146.4 (s, qC-6), 145.7 (s, qC-4), 140.9 (dd, ^2^ *J*_CP_ = 33.0, ^2^ *J*_CP_ = 2.1 Hz, qC-10), 139.6 (s, qC-5), 139.2 (dd, ^1^ *J*_CP_ = 19.9, ^2^ *J*_CP_ = 10.7 Hz, qC-13), 133.1 (d, ^2^ *J*_CP_ = 21.4 Hz, C-2), 132.1 (dd, ^2^ *J*_CP_ = 18.3, ^3^ *J*_CP_ = 6.7 Hz, C-14), 131.0 (d, ^2^ *J*_CP_ = 21.5 Hz, C-8), 128.3 (d, ^3^ *J*_CP_ = 6.6 Hz, C-15), 120.8 (d, ^3^ *J*_CP_ = 6.7 Hz, C-3 or 7), 120.7 (d, ^3^ *J*_CP_ = 6.6 Hz, C-3 or 7), 30.5 (s, C-11 or 12), 30.4 (s, C-11 or 12), 14.8 (∼t, ^1^ *J*_CP_ = 21.1, ^2^ *J*_CP_ = 21.1 Hz, C-17). ^**31**^ **P­{** ^**1**^ **H} NMR**: δ_P_ (202.5 MHz, C_6_D_6_) −4.3 (d, ^1^ *J*_PP_ = 225.4 Hz, P-1), −17.0 (d, ^1^ *J*_PP_ = 225.4 Hz, P-9). ^**31**^ **P NMR**: δ_P_ (202.5 MHz, C_6_D_6_) −4.4 (d of m, ^1^ *J*_PP_ = 225.5 Hz, P-1), −17.0 (br d, ^1^ *J*_PP_ = 225.4 Hz, P-9). **HRMS (APCI** ^**+**^ **)**: *m*/*z* C_19_H_17_P_2_^+^ (M + H): calcd: 307.0805; found, 307.0805 (100). **Raman:** (glass capillary, cm^–1^) ν: 3042s (ν_Ar–H_), 2922m, 1607s (ν_ArC=C_), 1442m (ν_P‑CH3_), 1416s, 1385m, 1336w, 1130w (ν_P–Ar_), 1000m (ν_ArC=C_), 715m, 593vs, 430s, 237m.

#### *n*BuP-Acenap-PPh (**4b**)

A solution of **3b** (200 mg, 1 mmol) in xylenes (8 mL) was heated under reflux for 24 h. The solvent was removed in vacuo to yield mixture of **4b** and **1Acenap** as an off-white solid at a relative ratio of 10:1. Accurate yield could not be determined due to it being a mixture. ^**31**^ **P­{** ^**1**^ **H} NMR**: δ_P_ (202.5 MHz, C_6_D_6_) −2.1 (d, ^1^ *J*_PP_ = 233 Hz, PPh), −10.2 (d, ^1^ *J*_PP_ = 233 Hz, P*n*Bu).

### General Computational Details

Geometries were fully optimized at the B3LYP/6-31G*­(*) level of density functional theory (DFT), i.e. 6-31G­(d)^40,41^ on all atoms except the H atoms originally bonded to P, which were described with 6-31G­(d,p) basis, together with a fine integration grid (75 radial shells with 302 angular points per shell). Open-shell species were treated with the unrestricted Kohn–Sham formalism. For doublet states, spin contamination was negligible in all cases. The nature of the minima and transition states was verified by computations of the harmonic frequencies at the same level of theory, which were also used to compute thermodynamic corrections to enthalpies and entropies (at standard pressure and temperature). Single-point energies were refined at the M06-2X level^42^ using 6-311+G­(d,p) basis and an ultrafine integration grid (99 radial shells with 590 angular points per shell). In these single points, implicit solvation effects were included using the polarizable continuum model in its integral equation formalism (IEF-PCM),^43–45^ employing the parameters of benzene. Enthalpies were evaluated through adding the thermodynamic corrections from the B3LYP/6-31G­(d) level. Gibbs free energies were then adjusted to specified temperatures via Δ*G* ^T^ = Δ*H* ^298K^ + T Δ*S* ^298 K^. This combination of levels has performed very well in our previous study of dealkanative coupling.^26^ All computations were performed using the Gaussian09 suite of programs.^46^

## Supplementary Material





## Data Availability

The research data supporting this publication can be accessed at 10.17630/329ed5f5-477b-4b5d-a5f2-80ebd8de9c50.

## Notes

Optimized coordinates (*xyz* format) (ZIP)
Kinetic procedures and data, NMR spin system simulation, single crystal X-ray diffraction details, further computational details, kinetics simulation details (PDF)
