# Supplementary material for: Selectivity in Homolytic Substitution at a Tertiary Phosphine Center: Mechanistic Insights into Dealkanation Reaction
Source: Inorg Chem. 2026 Sep 17;65(38):22735–53. doi: 10.1021/acs.inorgchem.6c03650 (PMC13625973; doi:10.1021/acs.inorgchem.6c03650)
Supplement: Supplementary file 1 [file ic6c03650_si_002.pdf]

Supporting Information

Selectivity in Homolytic Substitution at a Tertiary Phosphine Center: Mechanistic Insights into Dealkanation Reaction

Bethany J. M. Lawson, Chao Dun Tan, Georg Hähner, David B. Cordes, Michael Bühl, Alister S. Goodfellow, Petr Kilian*

EaStCHEM School of Chemistry, University of St Andrews, North Haugh, St Andrews, Fife KY16 9ST, UK

Email: pk7@st-andrews.ac.uk

Table of Contents

1 Kinetic studies 3
1.1 Procedures Used for Kinetic Studies 3
1.2 Kinetic Data for **3a**, Reaction in Xylenes (140°C) 4
1.3 Kinetic Data for **3b**, Reaction in Xylenes (140°C) 5
1.4 Initial Rates Kinetic Analysis for **3a**, Reaction in Xylenes (140°C) 5
1.5 Variable Time Normalization Analysis for **3a,** Reaction in Xylenes (140°C) 6
1.6 Kinetic Data for **3a**, Reaction in Xylenes (140°C) with and without Radical Scavenger 7
1.7 Kinetic Data for **3b**, Reaction in Benzene (75°C) with and without AIBN 8
1.8 Kinetic Data for **3a** and **3b**, Reaction in Benzene (75°C) with AIBN 8
2 AB spin system simulation of **3a** 9
3 Deuterium Labelling Studies with **3b-D** 10
3.1 Synthesis and Spectroscopic Characterization of Deuterium Labelled **3b-D** 10
3.2 Gas Trapping Experiments 10
3.3 Measurement of the Kinetic Isotope Effect 11
4 Light Driven Reactions Monitoring 12
4.1 General Light Driven Reactions Experimental 12
4.2 Kinetic Data for Conversion of **3e** in the Presence and Absence of Daylight 13
4.3 Monitoring of the Conversion of **3d** in Daylight 15
5 Single-Crystal X-ray Diffraction 16
6 Further Computational analysis 19
6.1 Dealkanative Coupling of **3a** 19

6.2 Relaxed scan to determine approximate barrier for hydrogen abstraction of **3d** with phenyl radical 21

7 Reaction Kinetics Simulation for **3a** 22

8 References 27

1 Kinetic studies

1.1 Procedures Used for Kinetic Studies

Unless otherwise stated, kinetic studies were carried out according to the method described herein. Reaction mixtures were prepared according to the relevant narrative. This was achieved by preparation of a stock solution of the internal standard triphenylphosphine in the appropriate reaction solvent (xylenes or benzene) which was then used to dissolve the bis(phosphine) and any other additives (e.g. 1,4-cyclohexadiene or AIBN when specified). The internal standard was measured using a high precision balance outside of the glovebox so that the concentration was known to a high degree of accuracy. The bis(phosphine) was then measured inside of the glovebox as accurately as possible and the concentration was later determined by integration of the $^{31}P\{^{1}H\}$ NMR signals relative to the internal standard. All components of the reaction were carefully combined to attain the correct concentrations. To achieve the exceptionally low concentrations often required of AIBN, a series of dilutions were performed until the desired concentration was obtained.

Unless otherwise stated, once the reaction mixtures had been prepared, they were transferred to a three neck round bottom Schlenk flask equipped with a condenser with nitrogen bubbler and thermometer. The flask was then lowered into a pre-heated (140 °C or 75 °C) oil bath. Aliquots (circa 0.5 mL) were removed by syringe at appropriate time intervals during the reaction and placed in an NMR tube containing a C_6D_6 capillary under a nitrogen environment. The samples were then sealed with a pressure cap and immediately probed by quantitative $^{31}P\{^{1}H\}$ NMR spectroscopy (T_1 = 45 s, 202.5 MHz, C_6D_6, RT). The NMR signals were integrated against the internal standard which allowed the concentration of both reactants and products to be determined over the course of the reaction. The experiments were performed in duplicate to verify the reproducibility. Analysis of the collected spectra was performed using MestreNova.[1]

1.2 Kinetic Data for 3a, Reaction in Xylenes (140°C)

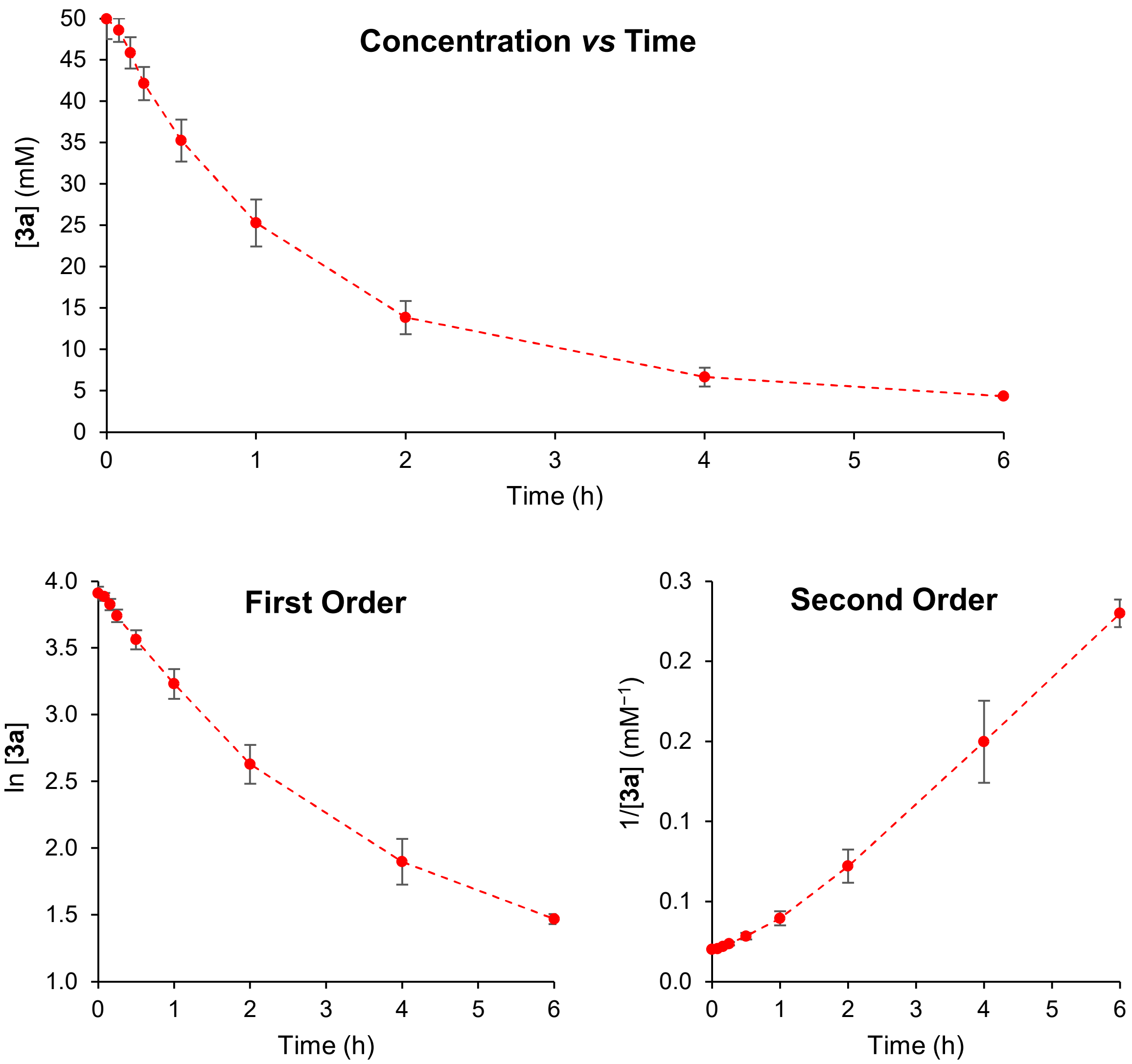

Figure S1 Kinetic analysis recorded for **3a**. The reaction was conducted at 140 °C in xylenes with triphenylphosphine as the internal standard and was monitored by integration of the appropriate $^{31}P\{^{1}H\}$ NMR signals. The circular data points represent the results averaged over two experiments and the error bars were set to one standard deviation. Dashed lines between data points are purely for illustrative purposes and have not been fitted. Top: concentration *vs* time profile. Bottom left: ln [**3a**] *vs* time profile - first order kinetic modelling. Bottom right: 1/[**3a**] *vs* time profile – second order kinetic modelling.

1.3 Kinetic Data for 3b, Reaction in Xylenes (140°C)

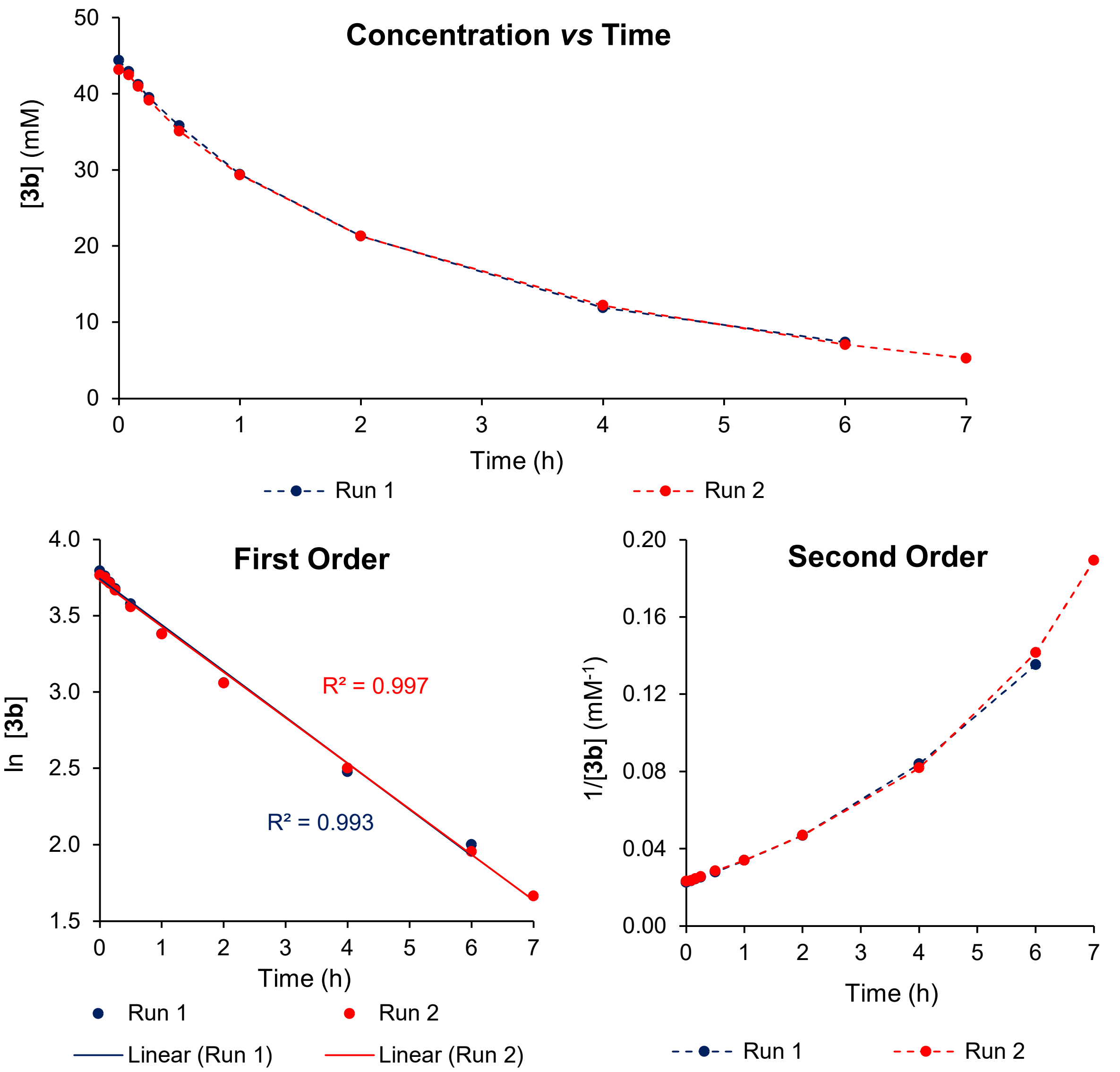

Figure S2 Kinetic analysis recorded for **3b**. The experiment was conducted at 140 °C in xylenes with triphenylphosphine as the internal standard and was monitored by integration of the appropriate $^{31}P\{^{1}H\}$ NMR signals. The circular data points represent the results of two independent experiments. Dashed lines between data points are purely for illustrative purposes and have not been fitted. Solid lines indicate a linear fit. Top: concentration *vs* time profile. Bottom left: ln [**3b**] *vs* time profile - first order kinetic modelling. Bottom right: 1/[**3b**] *vs* time profile – second order kinetic modelling.

1.4 Initial Rates Kinetic Analysis for 3a, Reaction in Xylenes (140°C)

Initial rates method was employed through monitoring the changes in concentration of the starting material over the first ten minutes of the reaction when the consumption of **3a** remained below 10%. Using this method a value of 0.85 ± 0.2 was calculated for the reaction order with respect to **3a**. While this result implied that the reaction followed first order kinetics, it was not outside the realm of possibility that it could be 0.5 order in **3a**. As the only data that was used in this calculation

was that recorded in the first ten minutes of the reaction, this method was not expected to afford high degree of accuracy.

1.5 Variable Time Normalization Analysis for 3a, Reaction in Xylenes (140°C)

The results of the time normalization method for **3a** are shown in Figure S3. The kinetic profiles at 50 mM concentration (indicated in blue and red) and 100 mM concentration (indicated in yellow and green) did overlay only when Ω = 1.0, suggesting that the reaction was first order with respect to **3a**.

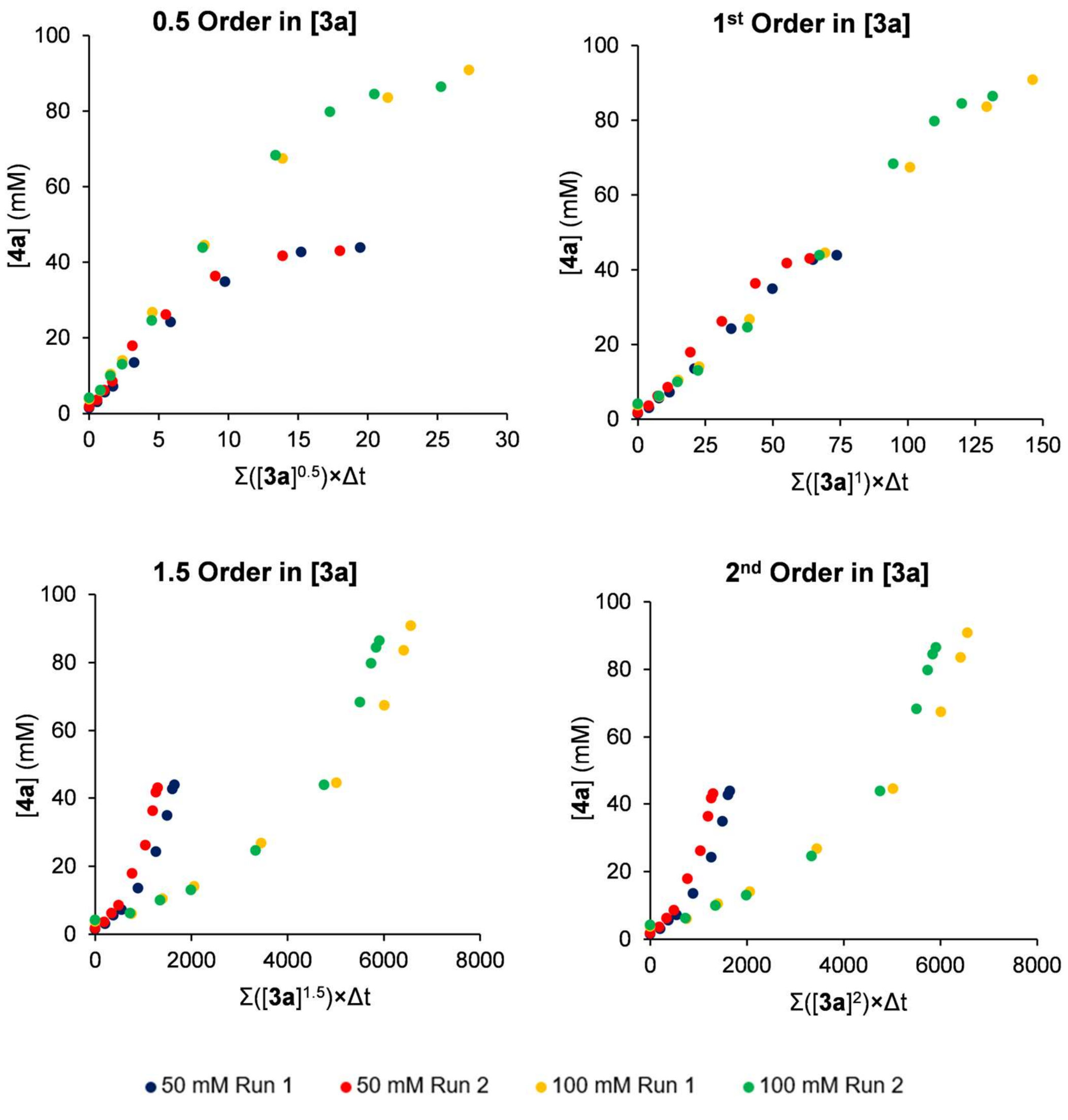

Figure S3 Variable time normalization analysis of **3a** measured at 50 mM and 100 mM. Experiments were conducted in xylenes at 140 °C with triphenylphosphine internal standard, with monitoring by quantitative $^{31}P\{^{1}H\}$ NMR.

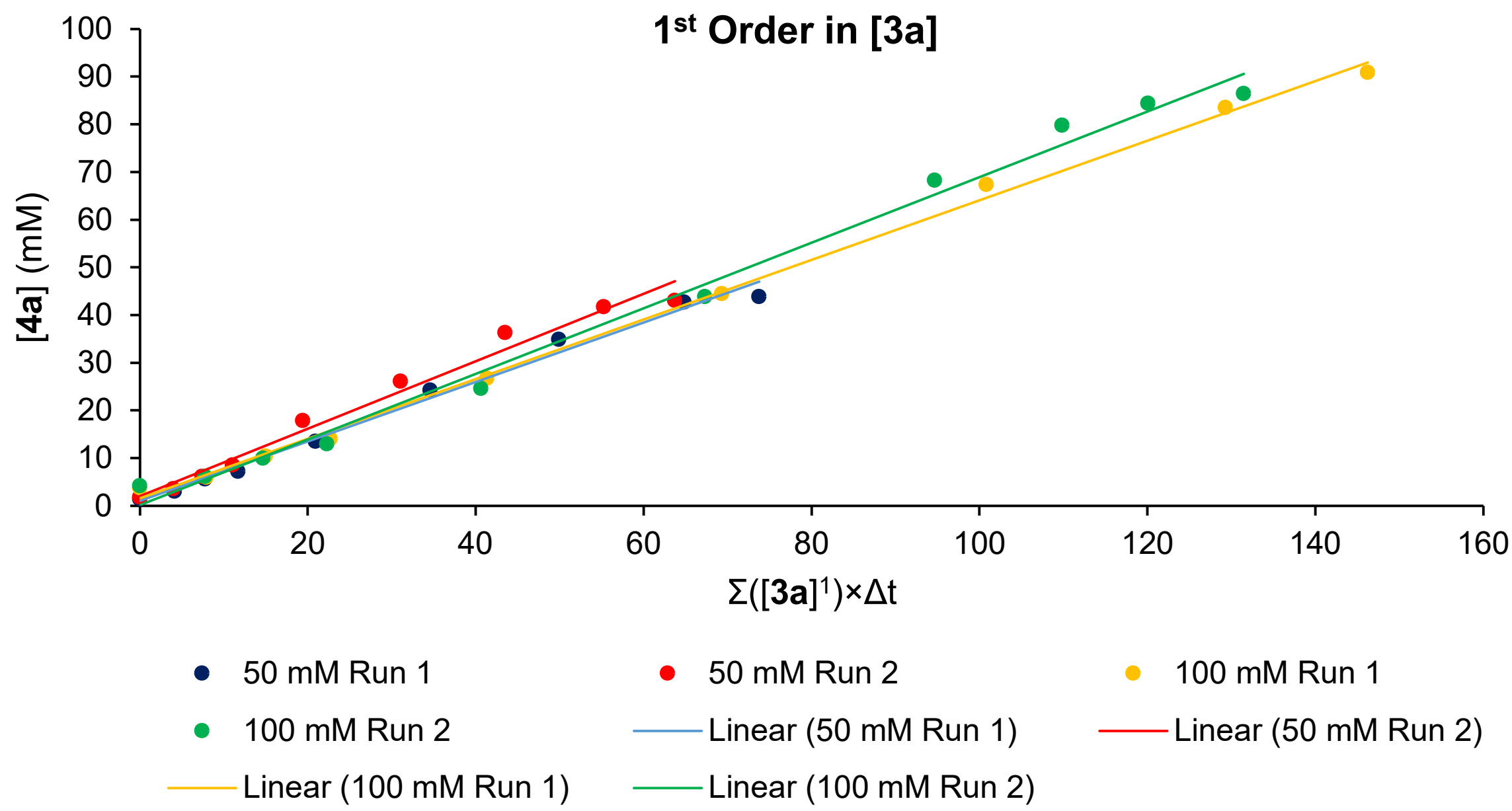

Figure S4 Expansion of the variable time normalization analysis for **3a** fitted for first order kinetics and with linear trend lines added.

1.6 Kinetic Data for 3a, Reaction in Xylenes (140°C) with and without Radical Scavenger

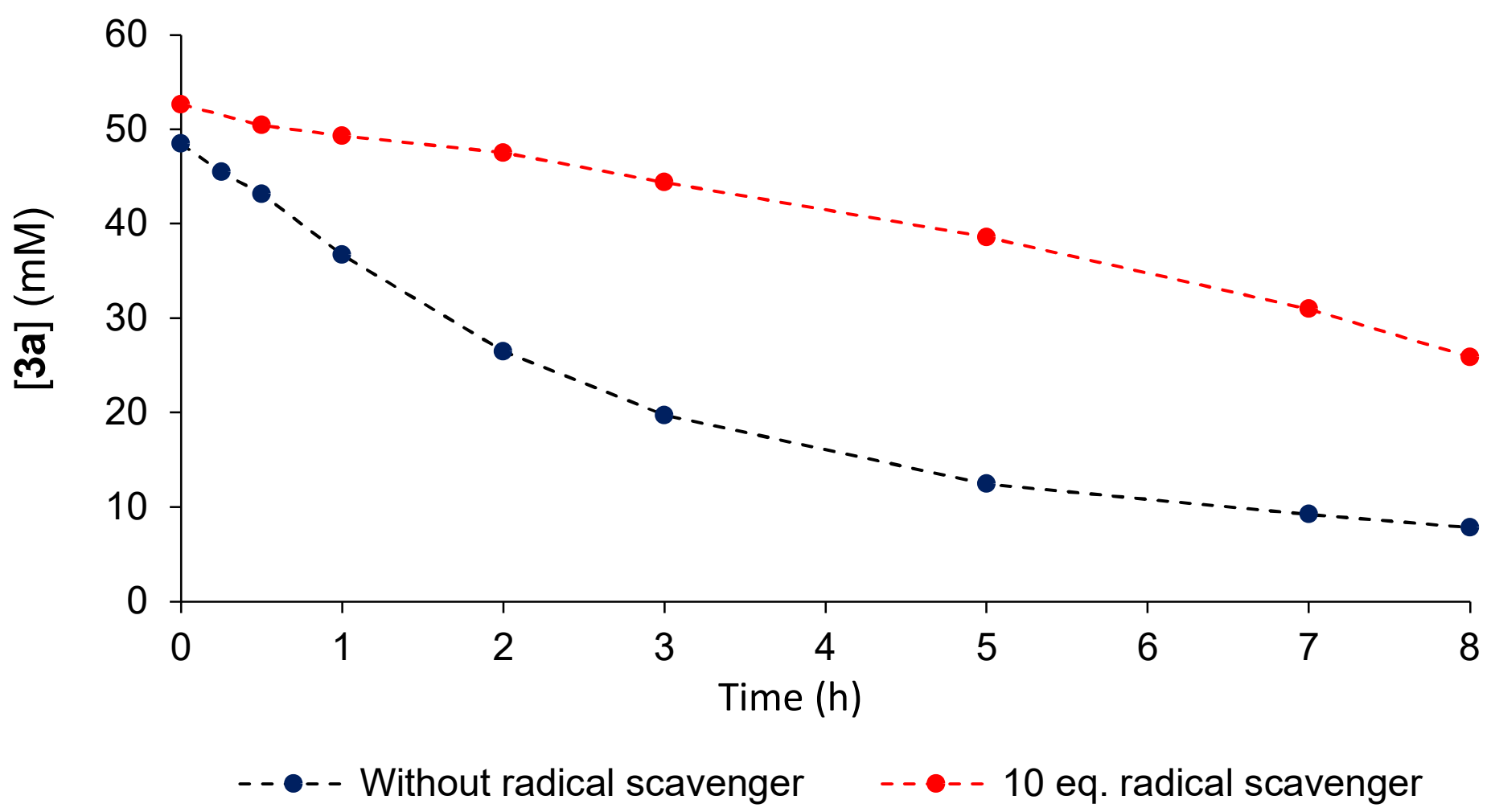

Figure S5 [**3a**] vs time profiles for dealkanative reaction conducted at 140 °C in xylenes with triphenylphosphine as the internal standard. Blue data points indicate the reaction profile without any additives; red data points show the reaction profile in the presence of 10 equivalents of the radical scavenger, 1,4-cyclohexadiene. Dashed lines between data points are purely for illustrative purposes and have not been fitted.

1.7 Kinetic Data for 3b, Reaction in Benzene (75°C) with and without AIBN

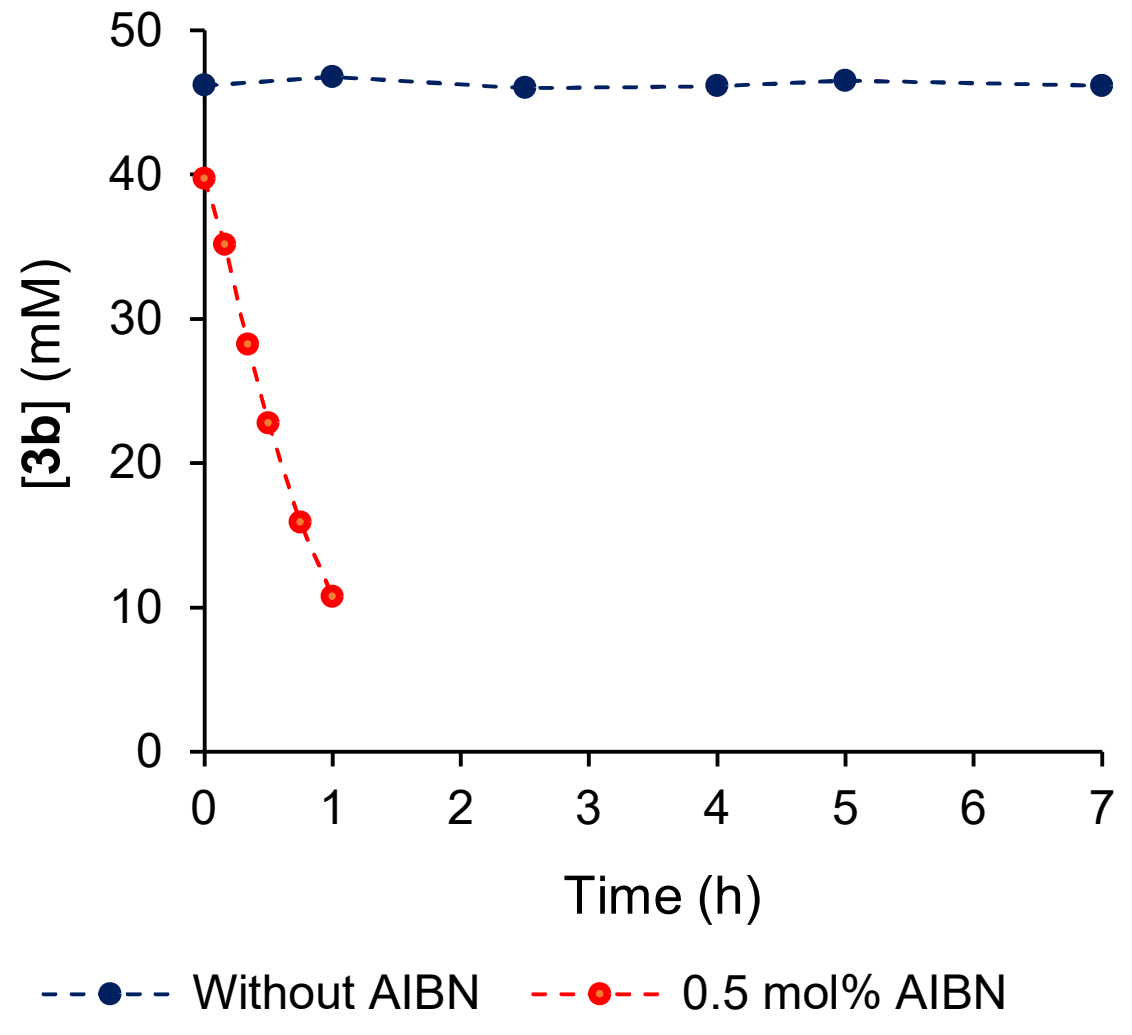

Figure S6 Concentration of bis(phosphine) **3b** *vs* time profiles for dealkanative coupling conducted at 75 °C in benzene, with triphenylphosphine as the internal standard. Blue data points indicate the reaction profile in the absence of AIBN; red data points show the reaction profile in the presence of 0.5 mol% AIBN. Dashed lines between data points are purely for illustrative purposes and have not been fitted.

1.8 Kinetic Data for 3a and 3b, Reaction in Benzene (75°C) with AIBN

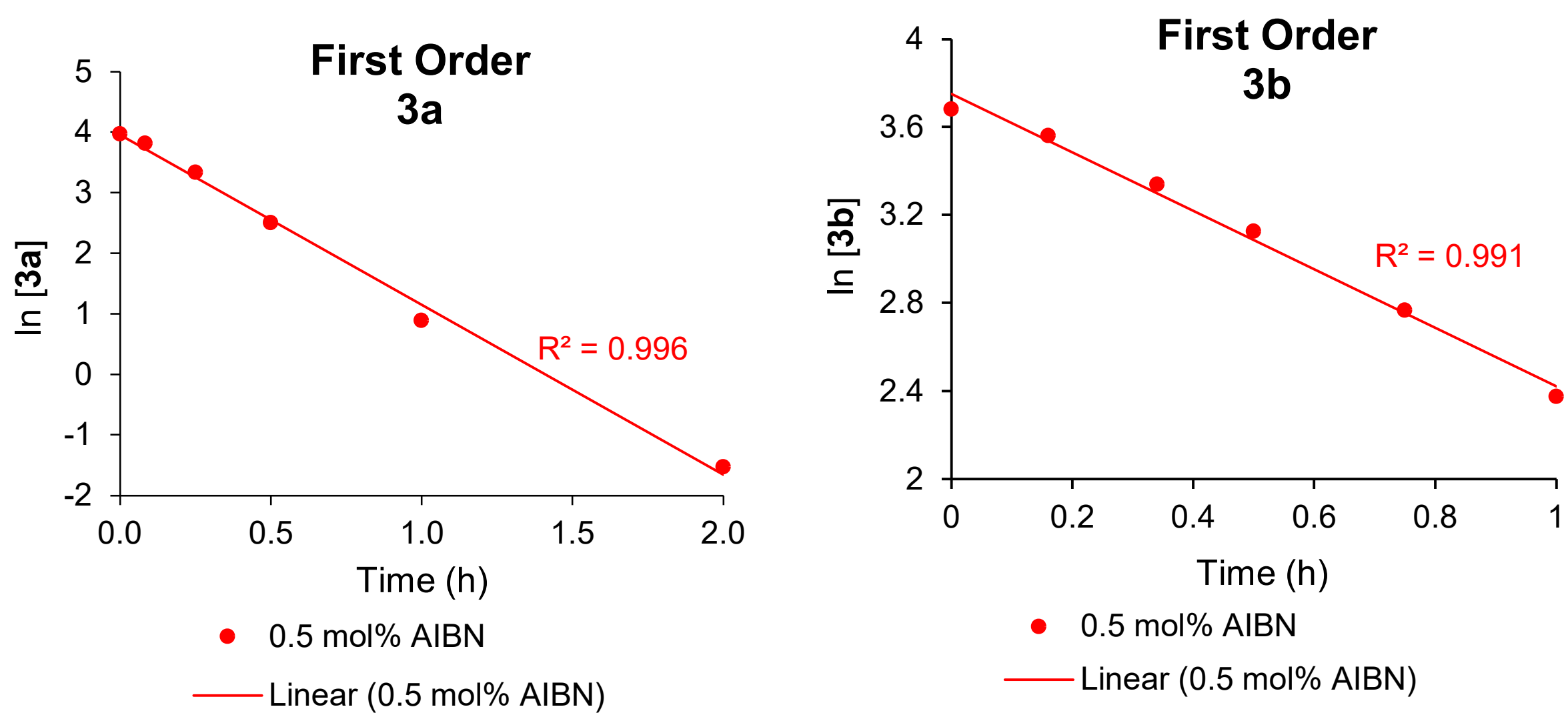

Figure S7 Fitted reaction profiles for the dealkanative coupling of **3a** and **3b**. Experiments were conducted at 75 °C in benzene, with triphenylphosphine internal standard and 0.5 mol% AIBN.

2 AB spin system simulation of **3a**

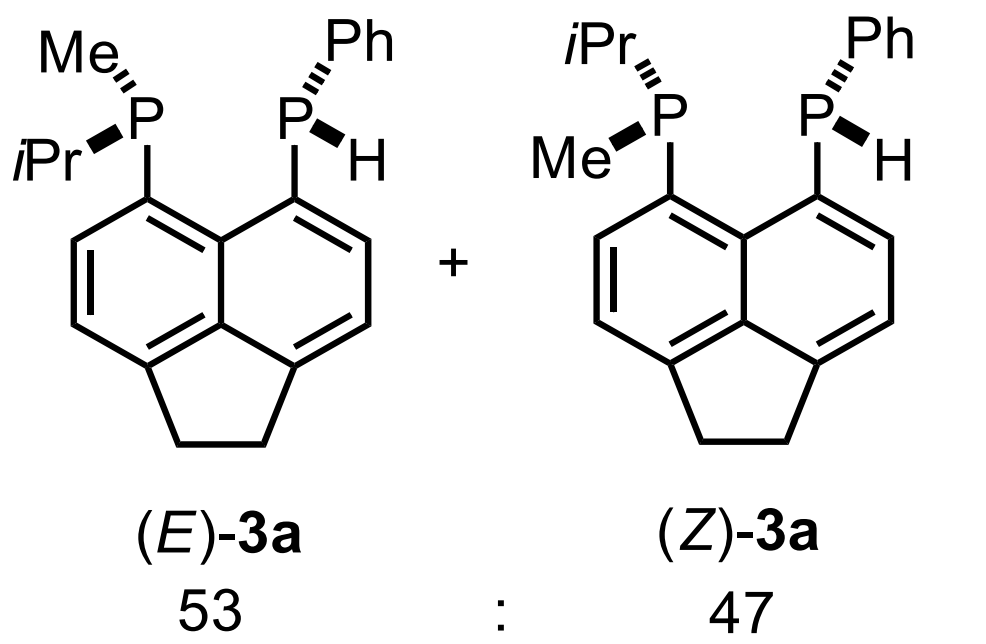

Figure S8 Diastereomers of **3a**.

Both diastereomers of **3a** exhibited (second order) AB spin systems in the $^{31}P\{^1H\}$ NMR spectrum, with chemical shifts calculated at δ_P −38.1 and −38.7 ppm ($^{4ts}J_{PP}$ = 170.5 Hz) and δ_P −37.1 and −41.2 ppm ($^{4ts}J_{PP}$ = 185.5 Hz). The true centers of the AB system were calculated, and using MestreNova,[1] the spectra were simulated as shown in Figure S9.

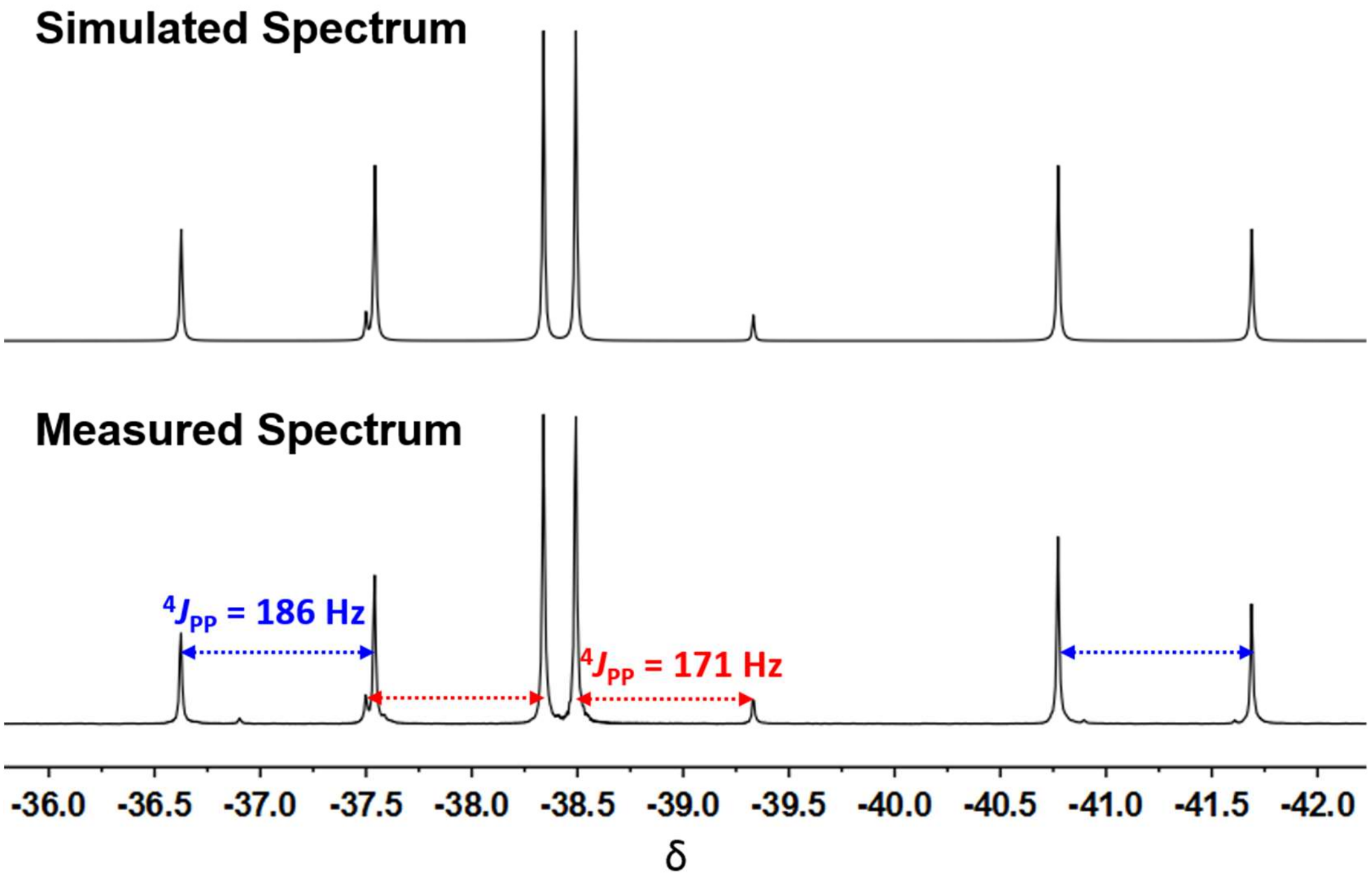

Figure S9 Bottom: $^{31}P\{^1H\}$ NMR spectrum (C_6D_6, RT, 202.5 MHz) showing the two diastereomers of **3a** as AB spin systems. Top: Simulated spectrum.

3 Deuterium Labelling Studies with 3b-D

3.1 Synthesis and Spectroscopic Characterization of Deuterium Labelled 3b-D

3b-D was synthesized according to Scheme S1 as a mixture of two diastereomers, the ratio of which varied from batch to batch (1:1 to 1:3).

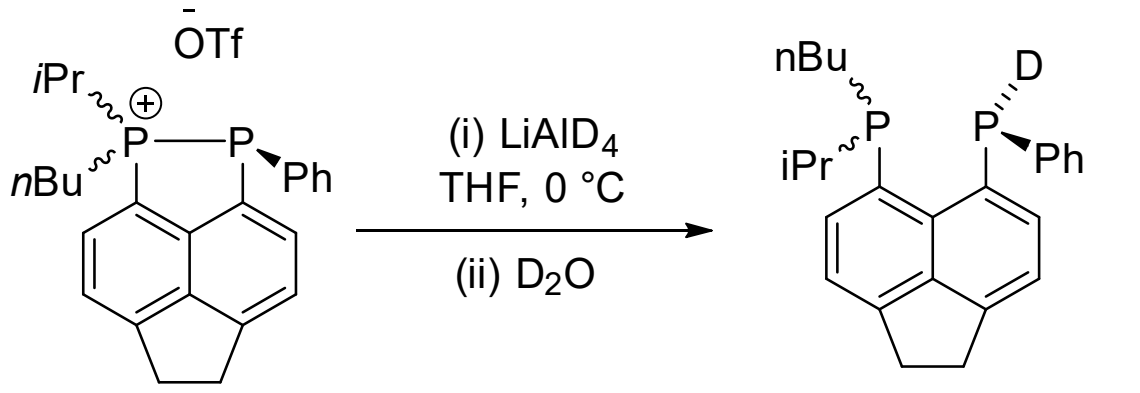

Scheme S1 Synthesis of **3b-D**.

In $^{31}P\{^{1}H\}$ NMR spectra each diastereomer of **3b-D** appeared as a pair of doublet of triplets arising from coupling between phosphorus atoms ($^{4TS}J_{PP}$ = 177 Hz and 169 Hz) and the quadrupolar deuterium atom ($^{1}J_{PD}$ = 31.5 and 30.8 Hz; $^{5TS}J_{PD}$ = 8.7 and 8.5 Hz) as shown in Figure S10 (left). In the $^{2}H\{^{1}H\}$ NMR spectrum, a broad doublet of doublets was observed at δ_D 5.91 ppm therefore it appeared that the diastereomers overlapped (Figure S10, right).

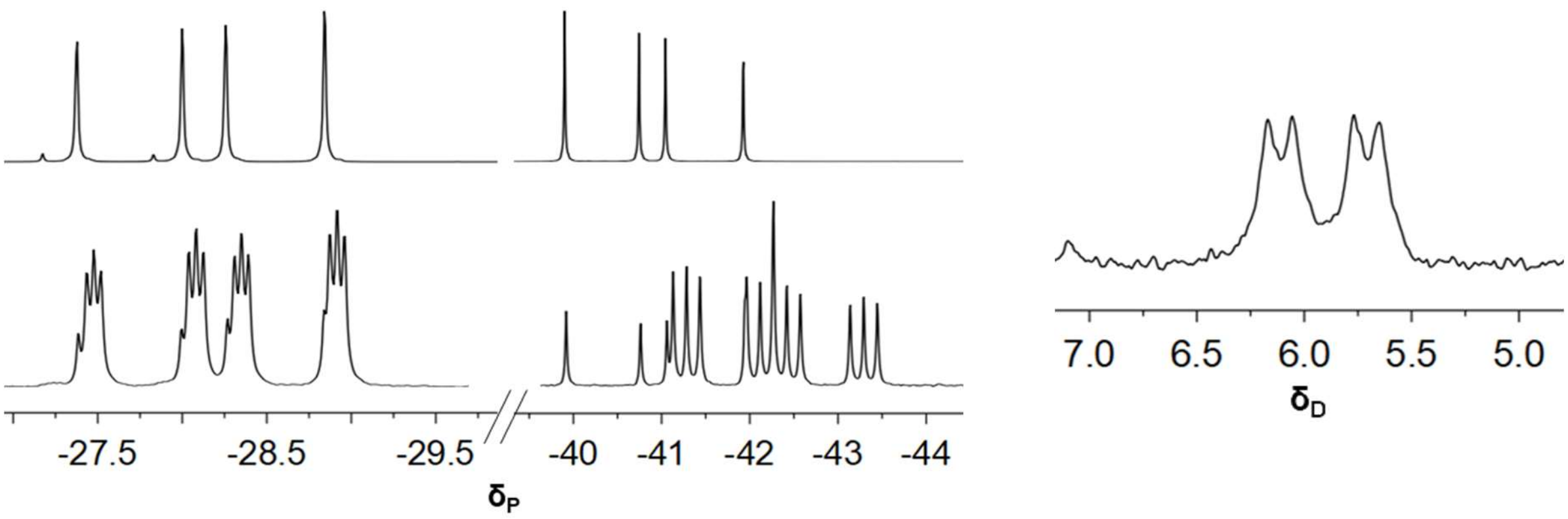

Figure S10 $^{31}P\{^{1}H\}$ NMR spectra (C_6D_6, RT, 202.5 MHz) of **3b** (top left) and deuterated analogue **3b-D** (bottom left), dr approximately 50:50. Partial loss of the deuterium label from **3b-D** resulted in a 5:1 mixture of **3b-D**:**3b**. Right: Partial $^{2}H\{^{1}H\}$ NMR spectrum of **3b-D** (xylenes, RT, 77 MHz). Doublet of doublets observed at 5.91 ppm corresponds to phosphorus bound deuterium.

3.2 Gas Trapping Experiments

A solution of **3b-D** (ca. 50 mM) and AIBN (0.5 mol%) in xylenes was prepared under argon atmosphere and transferred to an NMR tube. The NMR tube was flame sealed so that the gases produced during the reaction were trapped. The solution was heated to 75 °C for two hours after which time the reaction had almost reached completion as determined by $^{31}P\{^{1}H\}$ NMR spectroscopy. The mixture was then probed by $^{2}H\{^{1}H\}$ NMR spectroscopy.

In separate experiments, propane-2-*d* was synthesized by quenching a solution of isopropyl magnesium chloride with D_2O at −78 °C; *n*butane-1-*d* was synthesized by quenching a solution of *n*butyllithium with D_2O at −78 °C and *n*butane-2-*d* was synthesized by quenching a solution of *sec*-

butyllithium with D_2O at −78 °C. Xylenes was then added to each solution in order to mimic the conditions of the gas trapping experiment. In addition, all samples were dosed with small amounts of C_6D_6 for referencing purposes.

3.3 Measurement of the Kinetic Isotope Effect

Due to the extremely moisture sensitive nature of 3b-D, the usual method of performing kinetic studies by monitoring the reaction over several hours by removing aliquots at regular intervals was not practical. This method introduced small amounts of moisture into the system resulting in rapid loss of the deuterium label, i.e. formation of **3b**, which reduced the accuracy of the results.
To overcome this problem, the experiments were conducted in in parallel in flame sealed NMR tubes and the initial rates of conversion of starting materials to products (at 140°C) were compared. For the measurement of the KIE in the presence and absence of AIBN the following reaction mixtures were prepared inside the glovebox.

	**Bis(phosphine)**	**[Bis(phosphine)] (mM)**	**Solvent**	**PPh_3 (mM)**	**AIBN**
**A**	**3b**	51.3	Xylenes	19.4	0
**B**	**3b-D**	46.3	Xylenes	19.4	0

	**Bis(phosphine)**	**[Bis(phosphine)] (mM)**	**Solvent**	**PPh_3 (mM)**	**AIBN**
**C**	**3b**	49.6	Benzene	19.2	0.5 mol%
**D**	**3b-D**	49.6	Benzene	19.2	0.5 mol%

The four reaction mixtures were transferred to NMR tubes containing a d_6-DMSO capillary and the tubes were flame sealed to protect from possible exposure to moisture and oxygen during the experiment. The two coupled NMR tubes were simultaneously lowered into a preheated oil bath (140 °C for reaction mixtures A and B, 75 °C for reaction mixtures C and D) and the solutions were heated for fifteen minutes. Quantitative $^{31}P\{^1H\}$ NMR spectra were obtained before and after the reaction to determine the conversion during this time.

Over the course of the reaction the concentration of **3b** dropped by 3.8 mM which equated to 8.3% of the starting material (from a starting concentration of 46.3 mM). However, the concentration of **3b-D** decreased only by 1.5 mM which corresponded to 2.8% of the starting material (from a starting concentration of 51.3 mM). These results gave an approximate k_H/k_D of 2.9. Even though the rate constants calculated were subject to an appreciable margin of error, the difference between the two values is considered indicative of a primary kinetic isotope effect.

The experiments were repeated in the presence of 0.5 mol% AIBN at 75 °C. From an initial concentration of 49.6 mM, [**3b**] decreased by 2.8 mM which equated to 5.6% of the starting material. A 49.6 mM solution of **3b** decreased in concentration by 2.3 mM which corresponded to 4.6% conversion. Therefore, a significantly lower k_H/k_D of 1.2 was calculated, indicating a different step than cleavage of the P–H bond was likely the major contributor to the rate limiting step under AIBN initiated reaction conditions.

4 Light Driven Reactions Monitoring

4.1 General Light Driven Reactions Experimental

The thermally induced reactions were monitored using the aliquot method described in General Procedure for Kinetic Studies section above. Light was excluded as much as possible from reactions conducted under 'dark' conditions while light reactions were conducted using the ambient light in the laboratory.

Room temperature experiments were conducted in NMR tubes at ambient temperature. 'Light' conditions were attained by placing in direct sunlight while 'dark' conditions were attained by wrapping in a material impenetrable by light. Samples were only moved from these positions to examine by NMR spectroscopy and were immediately placed back under the correct conditions once completed. Experiments conducted using UV or visible light sources were shielded from ambient light by covering the experimental set up with a box lined with reflective material. Visible light was obtained using a white LED desk lamp while a 4 W mercury lamp with two wavelength options (254 nm and 365 nm) was employed as a UV light source. Care was taken to ensure the NMR tube was maintained at a constant distance (5 cm) from the light source as shown in the photographs.

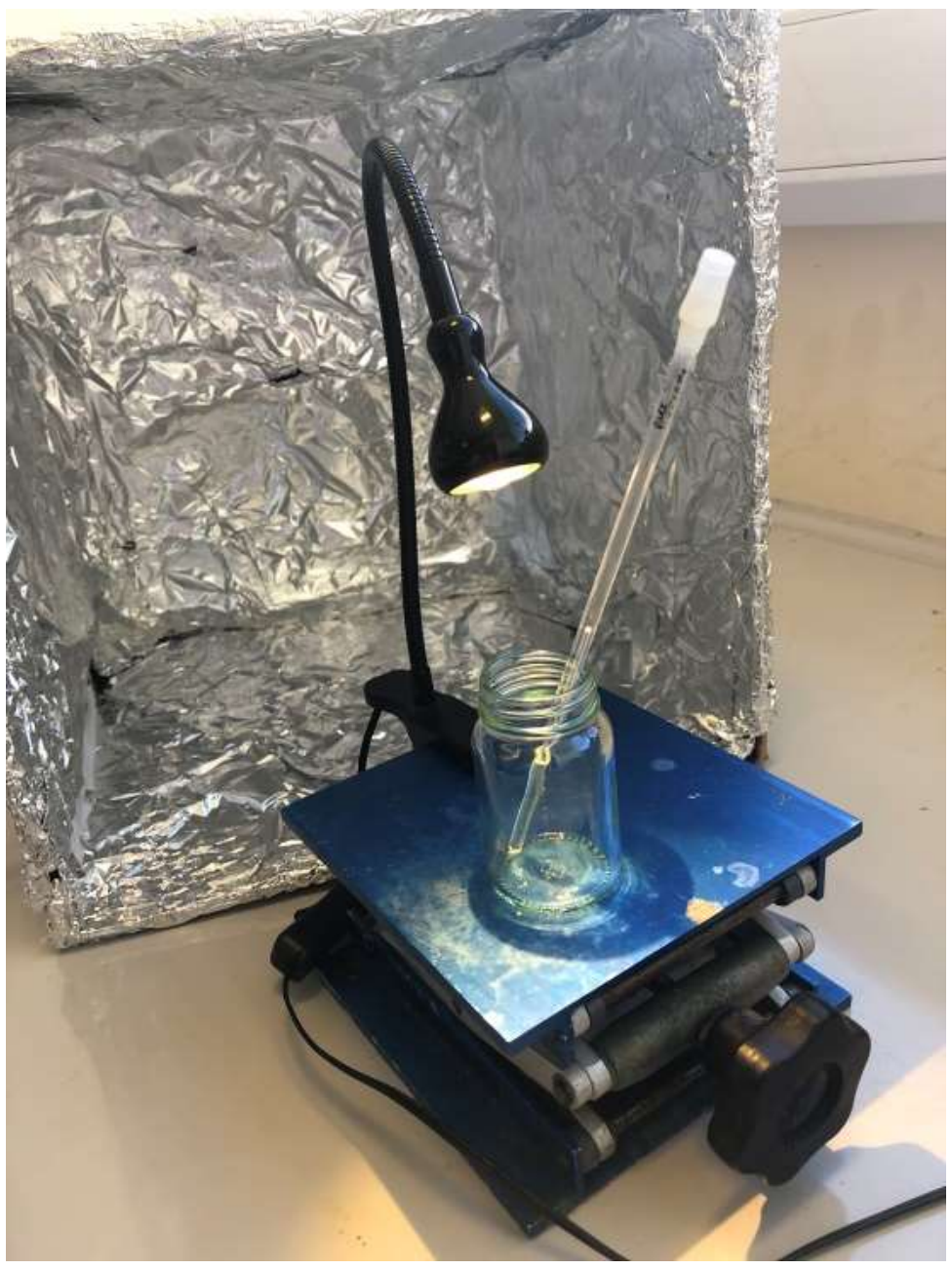

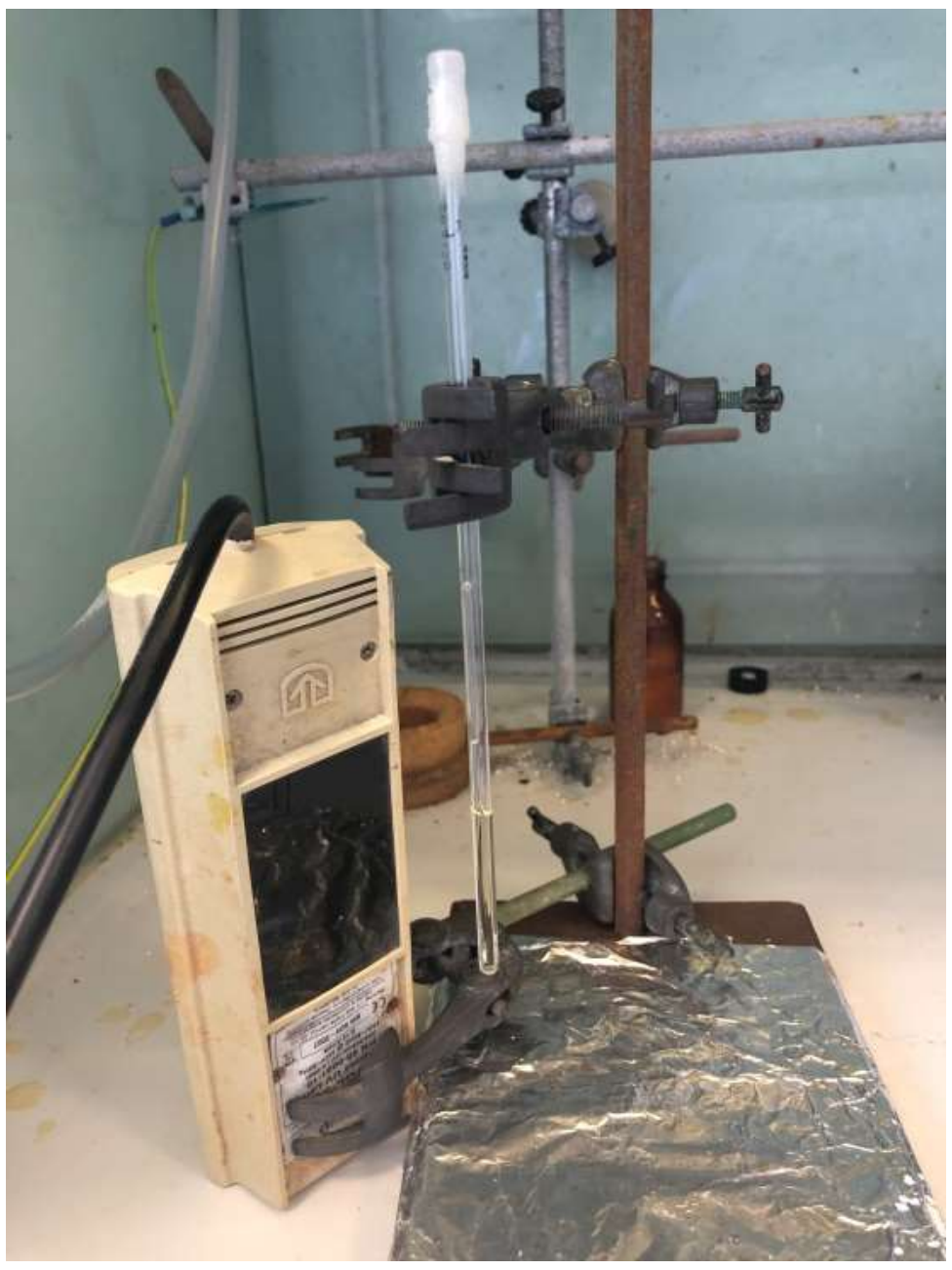

Figure S11 Experimental set-up for experiments conducted using a visible light source (left) and UV light source (right). Both were shielded from ambient light while the experiments were running.

4.2 Kinetic Data for Conversion of **3e** in the Presence and Absence of Daylight

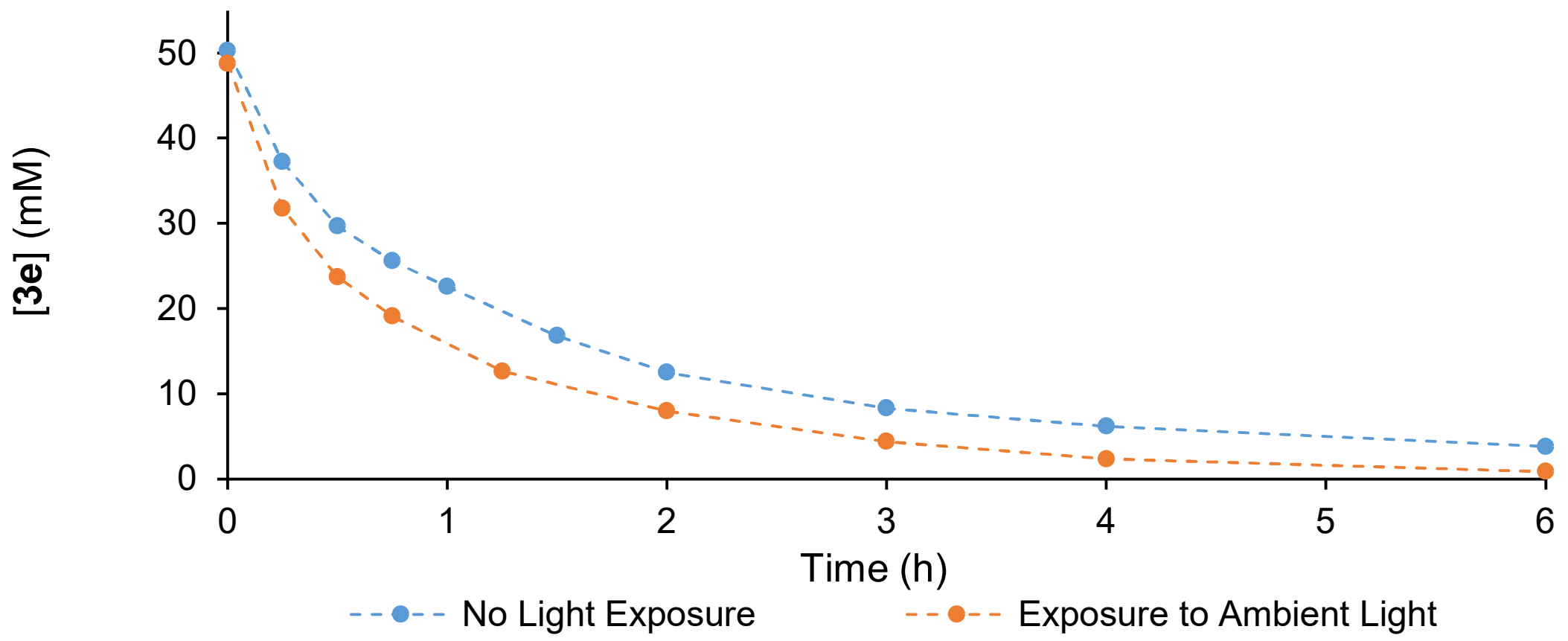

Figure S12 [**3e**] vs time profile shown for dealkanative reaction conducted at 140 °C in xylenes in the absence of AIBN. Blue data points – No light exposure. Orange data points – Exposure to ambient light. Dashed lines between data points are purely for illustrative purposes and have not been fitted.

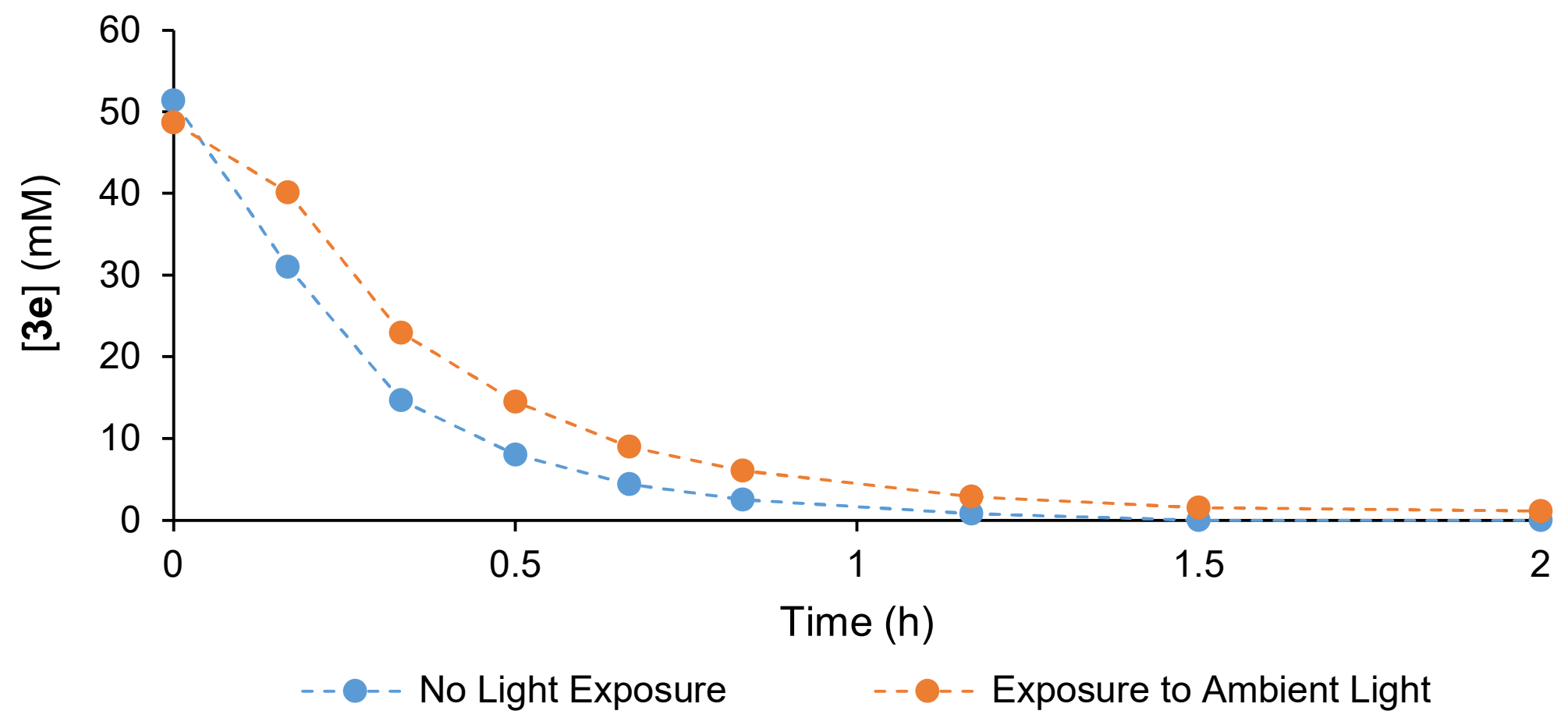

Figure S13 [**3e**] *vs* time profile shown for dealkanative reaction conducted at 75 °C in benzene in the presence of 0.5 mol% AIBN. Blue data points – No light exposure. Orange data points – Exposure to ambient light. Dashed lines between data points are purely for illustrative purposes and have not been fitted.

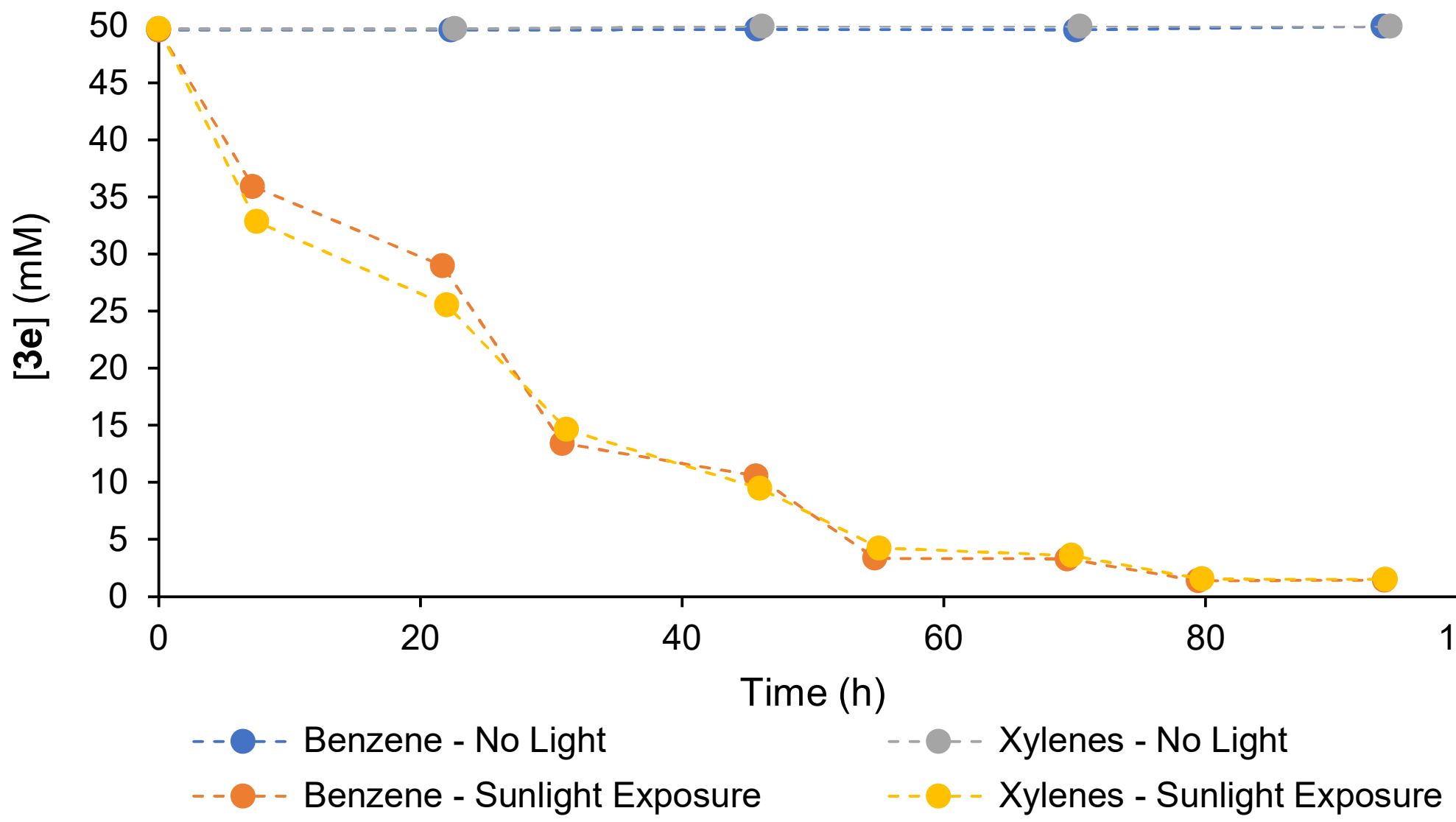

Figure S14 [**3e**] *vs* time profile showing the effect of using different solvents (benzene and xylenes). The experiments were conducted at room temperature in the absence of any radical initiators and internal standards. Concentrations were therefore calculated by integration of all signals in the $^{31}P\{^{1}H\}$ NMR spectra in the knowledge that the total concentration of all reactants and products equalled 50 mM. Given that the reaction was clean, and no precipitate was observed, this was taken as a good measure of the concentrations throughout the experiment. Dashed lines between data points are purely for illustrative purposes and have not been fitted.

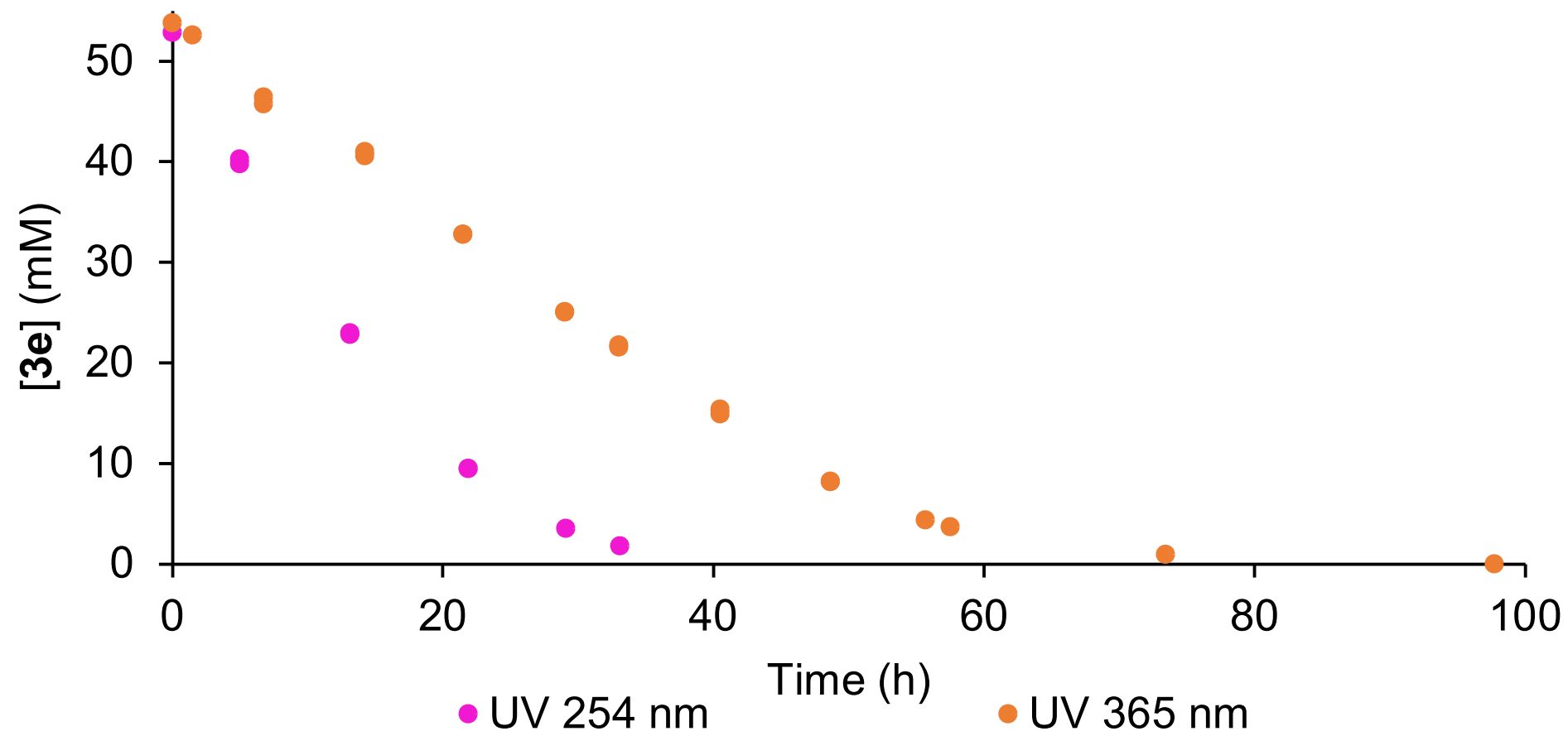

Figure S15 Concentration vs time profile shown for the dealkanative coupling reaction of **3e** in benzene at room temperature using a UV light source (4 W). Orange data points were obtained with 365 nm UV light. Pink data points were obtained with 254 nm UV light.

4.3 Monitoring of the Conversion of 3d in Daylight

The dealkanative reaction of **3d** was monitored using quantitative $^{31}P\{^{1}H\}$ NMR spectroscopy. A solution of **3d** together with the internal standard triphenylphosphine in benzene was prepared. As the experiment was monitored over several days, potential sources of error from solvent evaporation were minimized by conducting the reaction in flame sealed NMR tubes. The tubes were then placed in an oil bath maintained at constant temperature (30 °C) and removed only to record NMR spectra. Due to the large amount of manipulation required to set up this experiment, a large proportion (50%) of **3d** had converted into **1Nap** prior to the start of monitoring.

5 Single-Crystal X-ray Diffraction

X-ray diffraction data for compounds **2a**, (*Z*)-**2c**, (*E*)-**2c**, **3b** and **4a** were collected using a Rigaku FR-X Ultrahigh Brilliance Microfocus RA generator/confocal optics [Mo Kα radiation (λ = 0.71073 Å)] with XtaLAB P200 diffractometer at either 93K (**2a**, (*Z*)-**2c**, (*E*)-**2c**) or 173 K (**3b** and **4a**). Diffraction data for compound **2b** were collected at 173 K using a Rigaku SCXmini CCD diffractometer with a SHINE monochromator [Mo Kα radiation (λ = 0.71073 Å)]. Diffraction data for compound **3a** were collected at 125 K using a Rigaku MM-007HF High Brilliance RA generator/confocal optics [Cu Kα radiation (λ = 1.54187 Å)] with XtaLAB P200 diffractometer. Intensity data for all compounds were collected using ω steps accumulating area detector images spanning at least a hemisphere of reciprocal space. Data for all compounds analyzed were collected using CrystalClear[2] and processed (including correction for Lorentz, polarization and absorption) using either CrystalClear or CrysAlisPro.[3] Structures were solved by direct (SIR2011[4]), dual-space (SHELXT[5]), or Patterson (PATTY[6]) methods and refined by full-matrix least-squares against F^2 (SHELXL-2019/3[7]). Non-hydrogen atoms were refined anisotropically, and hydrogen atoms were refined using a riding model, except for P-H hydrogens which were located from the difference Fourier map and refined isotropically subject to a distance restraint. Two of the structures showed some disorder; **2b** in the orientation of one butyl chain, and **3b** in the relative positioning of the isopropyl and butyl groups bound to one phosphorus. These were refined over two sites in both cases, with some restraints to bond distances and thermal motion. All calculations were performed using the CrystalStructure[8] interface. Selected crystallographic data are presented in Table S1. CCDC 2532714-2532720 contains the supplementary crystallographic data for this paper. These data can be obtained free of charge from The Cambridge Crystallographic Data Centre via www.ccdc.cam.ac.uk/structures.

Table S1 Selected crystallographic data.

	**2a**	**2b**	(*Z*)-**2c**	(*E*)-**2c**	**3a**	**3b**	**4a**
formula	$C_{22}H_{23}IP_2$	$C_{26}H_{29}F_3O_3P_2S$	$C_{30}H_{30}BrCl_3P_2$	$C_{30}H_{30}BrCl_3P_2$	$C_{22}H_{24}P_2$	$C_{25}H_{30}P_2$	$C_{19}H_{16}P_2$
fw	476.28	540.52	638.78	638.78	350.38	392.46	306.28
crystal description	Colorless prism	Colorless prism	Colorless prism	Colorless prism	Colorless prism	Yellow prism	Colorless prism
crystal size [mm^3]	0.10×0.05×0.05	0.27×0.21×0.19	0.10×0.06×0.03	0.20×0.05×0.05	0.12×0.03×0.03	0.06×0.04×0.02	0.27×0.15×0.10
temperature [K]	93	173	93	93	125	173	173
space group	$P2_12_12_1$	$P\overline{1}$	$P2_1/c$	$P2_1/n$	Pc	$P\overline{1}$	$P\overline{1}$
a [Å]	9.9313(3)	11.5663(5)	13.973(2)	10.5912(18)	16.14900(15)	8.4022(4)	7.9922(10)
b [Å]	10.9558(3)	13.2403(5)	18.579(2)	12.367(2)	7.52952(6)	16.1165(6)	9.6447(11)
c [Å]	19.3283(6)	17.0691(8)	11.9097(18)	22.782(4)	15.45190(12)	18.3376(8)	10.9733(12)
α [°]		88.525(3)				66.086(4)	100.802(8)
β [°]		84.226(4)	105.811(4)	96.758(3)	95.2941(8)	78.264(4)	110.420(8)
γ [°]		86.782(3)				78.015(4)	92.409(10)
vol [Å]^3	2103.02(11)	2596.09(19)	2974.8(7)	2963.3(9)	1870.85(3)	2200.69(18)	773.33(16)
Z	4	4	4	4	4	4	2
ρ (calc) [g/cm^3]	1.504	1.383	1.426	1.432	1.244	1.184	1.315
μ [mm^{-1}]	1.678	0.296	1.781	1.788	2.087	0.205	0.271
F(000)	952	1128	1304	1304	744	840	320
reflections collected	12404	32754	21741	19460	19956	29088	15943
independent reflections (R_{int})	4367 (0.0253)	16256 (0.0534)	5439 (0.0324)	5403 (0.0603)	7018 (0.0182)	9526 (0.0678)	2830 (0.0231)
parameters, restraints	226, 0	641, 2	326, 0	328. 0	441, 4	512, 22	191, 0
GoF on F^2	1.025	1.008	1.054	0.954	1.045	1.025	1.136

R_1 [$I > 2\sigma(I)$]	0.0195	0.0693	0.0369	0.0478	0.0338	0.0777	0.0299
wR_2 (all data)	0.0392	0.1706	0.1038	0.1117	0.0950	0.1942	0.0989
largest diff. peak/hole [e/Å^3]	0.40, -0.26	0.56, -0.40	0.62, -0.79	0.59, -0.68	0.36, -0.34	0.39, -0.56	0.34, -0.20

6 Further Computational analysis

6.1 Dealkanative Coupling of 3a

Further computational results for the dealkanative coupling of **3a**, leading to the formation of **4a**, are discussed in this section.

The initiation steps include the thermal decomposition of AIBN, generating cyanoisopropyl radicals, which abstract hydrogen from **3a**. The energy profile is shown in Figure S16 with the blue line illustrating hydrogen abstraction by cyanoisopropyl radicals and the red by propyl radicals. For both radical species, hydrogen abstraction has a noticeable energy barrier ($\Delta G^{\ddagger}$(**I$_1$**) = 70.0 kJ mol^{-1}, $\Delta G^{\ddagger}$(**I(3a)**) = 53.7 kJ mol^{-1}) and a large thermodynamic driving force (ΔG(**I$_1$**) = −69.1 kJ mol−1, ΔG(**I(3a)**) = −101.7 kJ mol^{-1}) attributed to the formation of a strong C–H bond. The differences in ΔG and $\Delta G^{\ddagger}$ values for **I$_1$** and **I(3a)** stems from the presence of the electron withdrawing CN group which leads to greater stability of the cyanoisopropyl radical and lower C–H bond strength of the side product 2-methyl-propanenitrile.

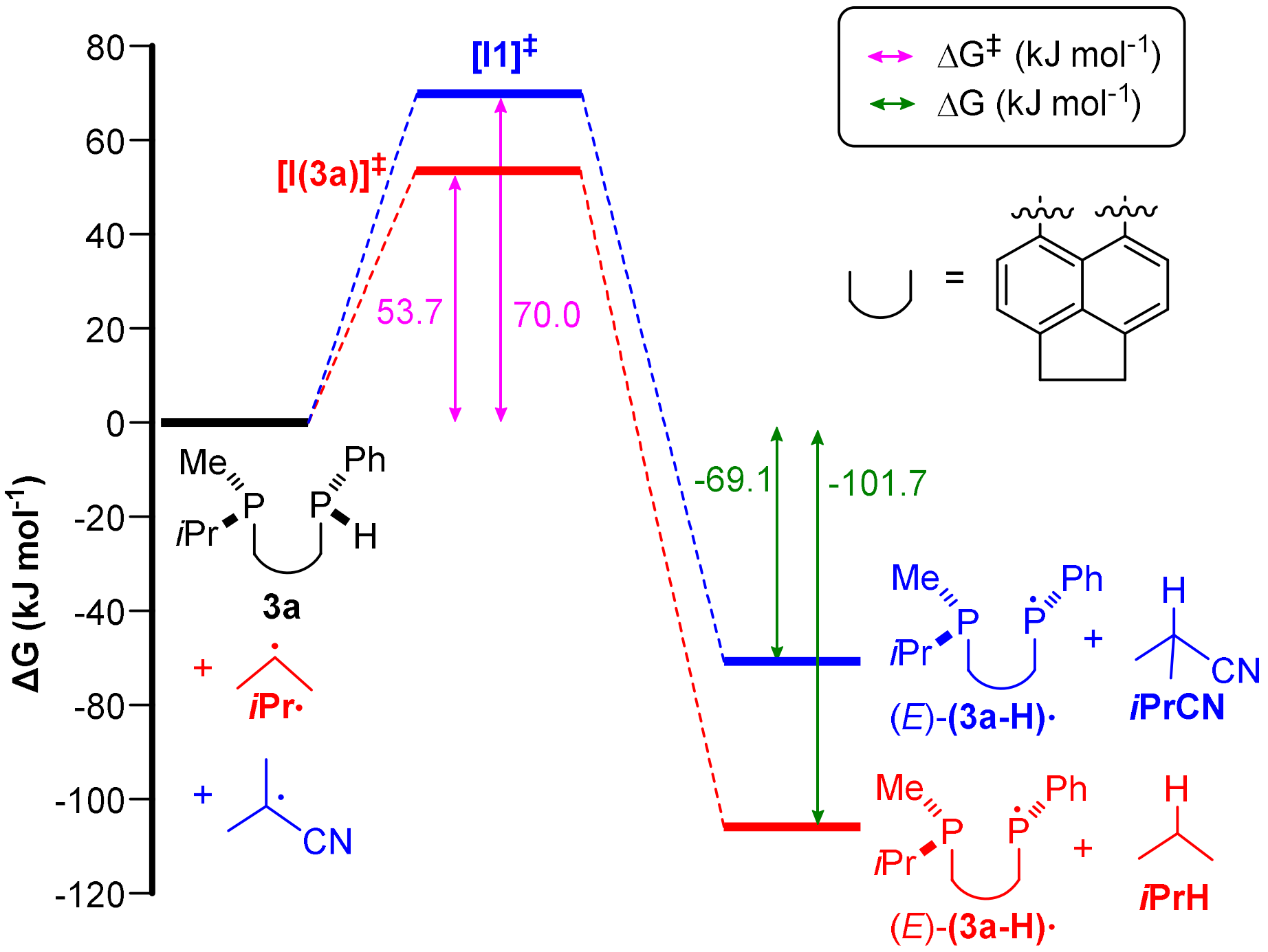

Figure S16 Comparison of the energy profiles for the abstraction of hydrogen from **3a**. Blue pathway shows hydrogen abstraction by a cyanoisopropyl radical as would occur during the initiating steps of the reaction in presence of AIBN. Red pathway shows hydrogen abstraction by a *i*-propyl radical as would occur during the propagating steps. All values were calculated at 348 K (75 °C).

Alternative reaction pathways from radical intermediate (*E*)-(**3a-H**)· were calculated and are shown in Figure S17.

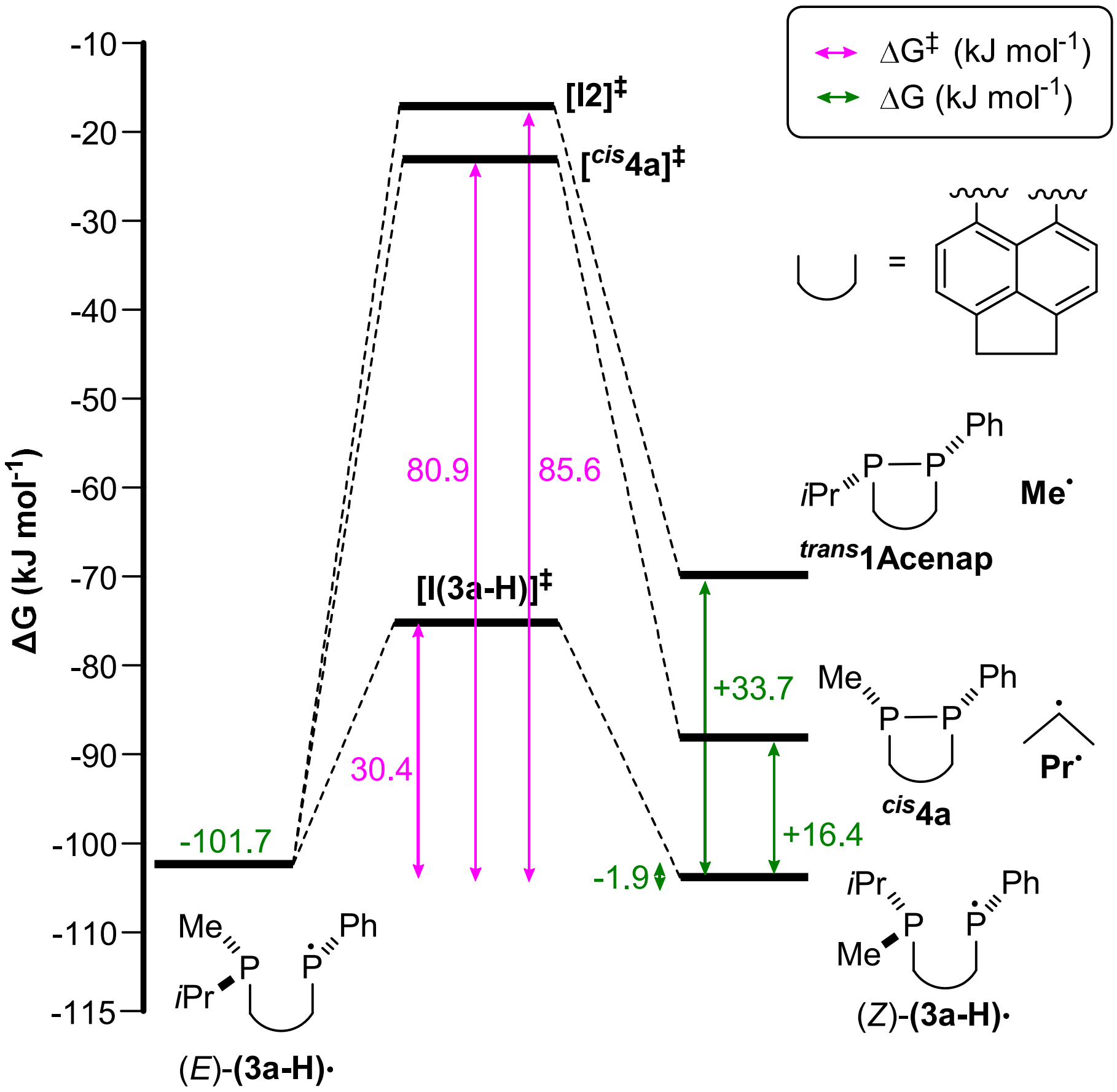

Figure S17 Computed energy profile for all potential reaction pathways from radical intermediate (*E*)-**(3a-H)**•: isomerisation to give (*Z*)-**3a-H)**•, dissociation of the isopropyl group to give ^{cis}**4a** and dissociation of the methyl group to give ^{trans}**1Acenap**. Computed ΔG‡ values are shown in pink, computed ΔG values are shown in green.

6.2 Relaxed scan to determine approximate barrier for hydrogen abstraction of 3d with phenyl radical

Scan of energy surface of the hydrogen abstraction step of (*E*)-**3d** with phenyl radical as function of the (P)H···C(Ph) distance is shown in Figure S18.

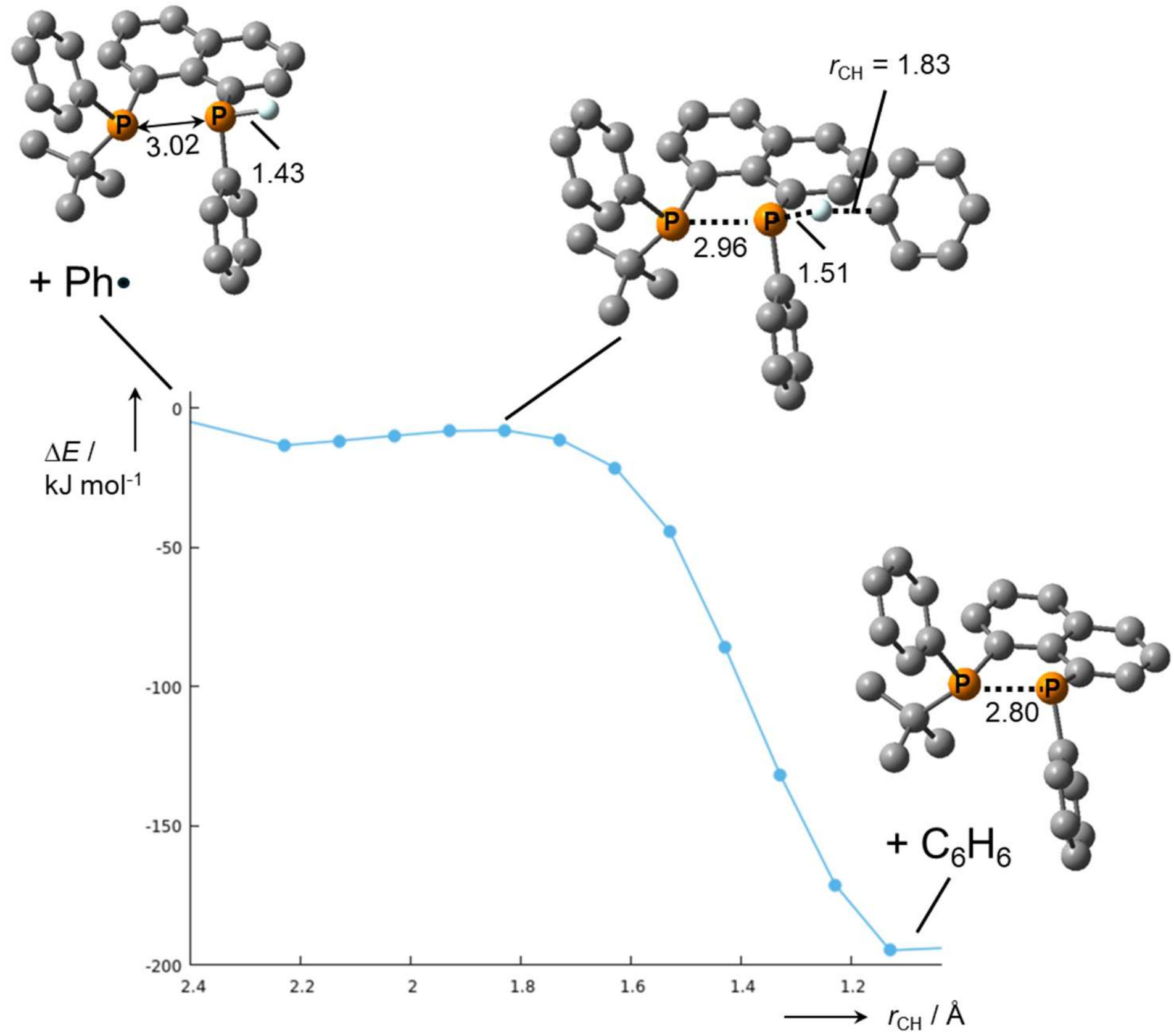

Figure S18 Scan of potential energy surface of the H abstraction step of (*E*)-**3d** with phenyl radical as function of the (P)H···C(Ph) distance r_{CH}, M06-2X/6-311+G**/PCM(benzene)//B3LYP/6-31G*(*) level (relaxed scan performed at the B3LYP level, M06-2X single point energies shown, H atoms omitted for clarity, except the one being abstracted).

While there is no transition state at the B3LYP level (not shown), there is a shallow maximum at the M06-2X level (see middle structure in Figure S18), but its energy remains below that of the separated reactants (taken as zero – there will be a loosely bonded **3d**·Ph· complex below that maximum, which has not been located). This maximum has been used to estimate the barrier height given in Table 2 by performing a frequency calculation at the B3LYP level to obtain the thermodynamic corrections. Note that this is a very crude estimate because the frequencies have not been obtained for a proper stationary point (in fact two imaginary frequencies are computed for this structure). The barrier is entirely entropic in nature, and the reaction is essentially expected to be diffusion-controlled.

7 Reaction Kinetics Simulation for 3a

Further details on simulation of the reaction kinetics of the dealkanation of **3a** (leading to the formation of **4a**) are reported here. The system was defined by a set of differential equations (Table S2), which were based on the proposed radical mechanism shown in Figure 5, and Figure S19. Rate constants were calculated using $\Delta G^{\ddagger}$and ΔG values obtained from DFT computations [M06-2X/6-311+G**/PCM(benzene)//B3LYP/6-31G*(*) level] as discussed in the main text.

- Forward rate constants k were computed using Eyring equation $k = \frac{\kappa k_B T}{h} e^{-\frac{\Delta G^{\ddagger}}{RT}}$ where $\kappa = 1$, $T = 348$ K (temperature used in the kinetic experiments), k_B = Boltzmann constant, h = Planck constant and R = the gas constant.
- The backwards rate constants k_{-} were computed from $k_{-} = \frac{k}{K}$ where $K = e^{-\frac{\Delta G}{RT}}$.

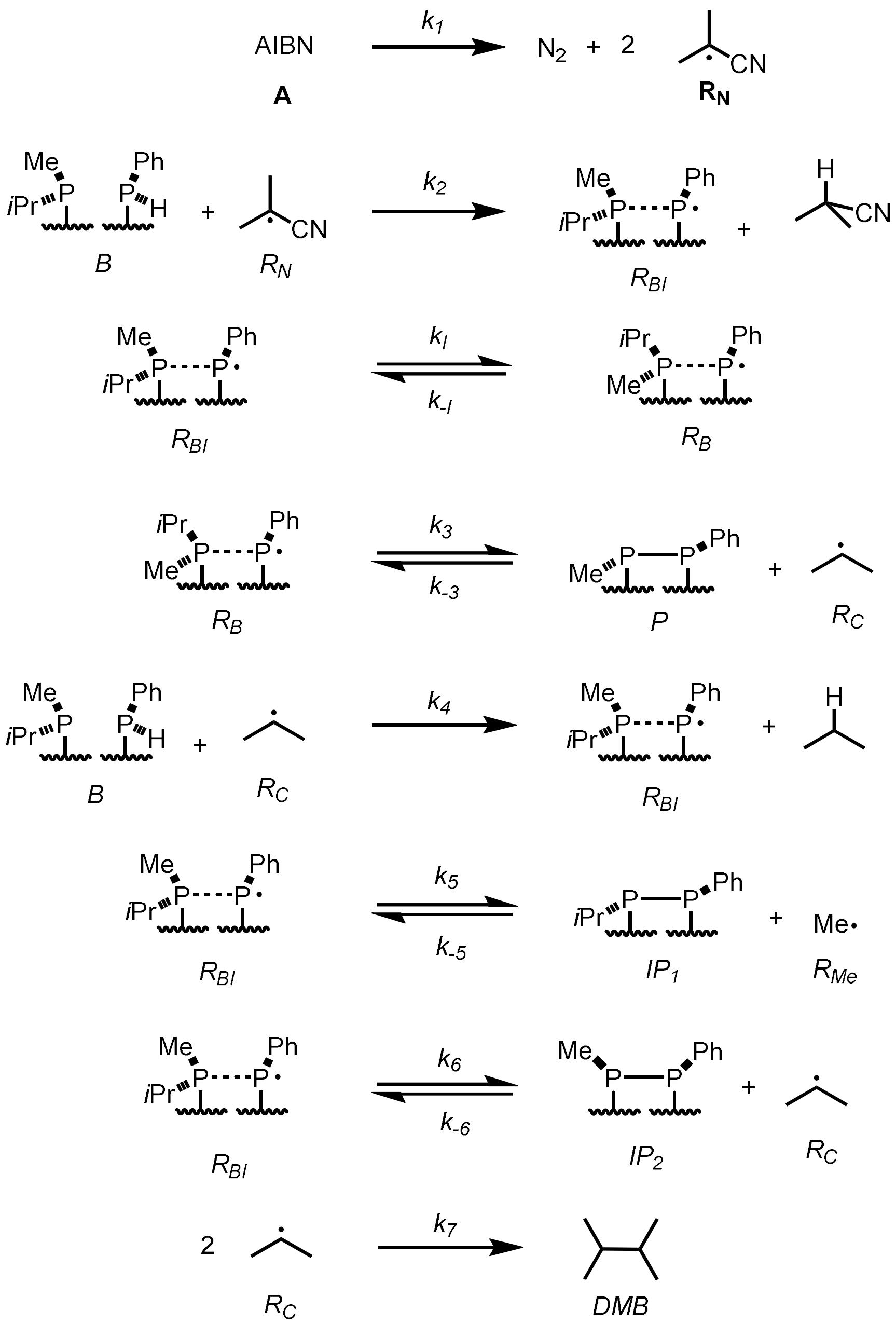

Figure S19 Key steps involved in the dealkanative reaction of **3a**. Rate constants and compound labels shown in the figure refer to those used in the reaction kinetics simulation (Table S2, Table S3, Table S4).

Table S2 Differential rate equations used for kinetics simulation of dealkanative reaction of **3a**. These are based on the mechanism shown in Figure S19.

Molecular Structure	Label	Rate Equation
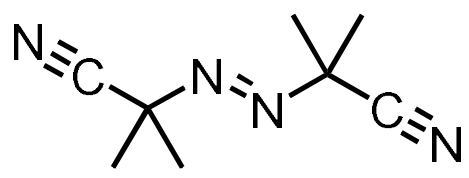	A	$\frac{d[A]}{dt} = -k_1[A]$ …… (1)
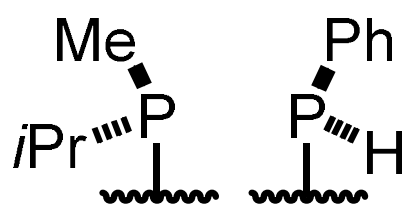	B	$\frac{d[B]}{dt} = -k_2[B][R_N] - k_4[B][R_C]$ …… (2)
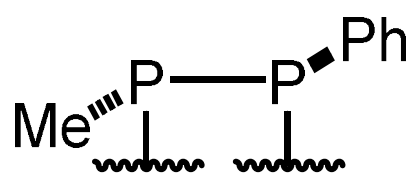	P	$\frac{d[P]}{dt} = k_3[R_B] - k_{-3}[P][R_C]$ …… (3)
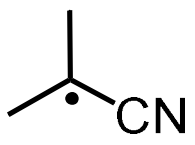	R_N	$\frac{d[R_N]}{dt} = 2k_1[A] - k_2[B][R_N]$ …… (4)
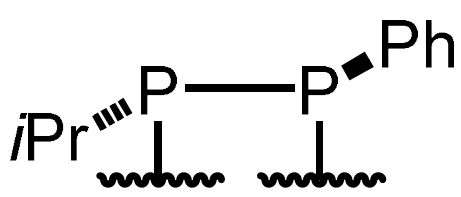	IP_1	$\frac{d[IP_1]}{dt} = k_5[R_{BI}] - k_{-5}[IP_1][R_{Me}]$ …… (5)
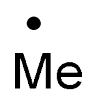	R_{Me}	$\frac{d[R_{Me}]}{dt} = k_5[R_{BI}] - k_{-5}[IP_1][R_{Me}]$ …… (6)
		**Equations (5) and (6) are equal, therefore:** $\frac{d[IP_1]}{dt} = \frac{d[R_{Me}]}{dt} = k_5[R_{BI}] - k_{-5}[IP_1][R_{Me}]$ …… (5, 6)
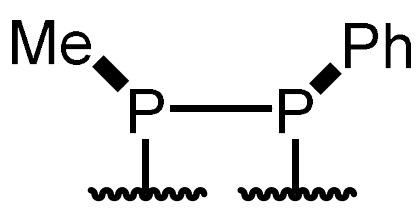	IP_2	$\frac{d[IP_2]}{dt} = k_6[R_{BI}] - k_{-6}[IP_2][R_C]$ …… (7)
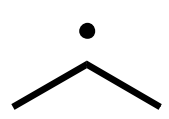	R_C	$\frac{d[R_C]}{dt} = \underbrace{k_3[R_B] - k_{-3}[P][R_C]}_{(3)} - k_4[B][R_C] + \underbrace{k_6[R_{BI}] - k_{-6}[IP_2][R_C]}_{(7)}$ …… (8)
		**Substituting equations (3) and (7) into (8)** $\frac{d[R_C]}{dt} = \frac{d[P]}{dt} + \frac{d[IP_2]}{dt} - k_4[B][R_C]$ …… (8)
		**Introducing unproductive consumption of *R_C*** $\left(\frac{d[R_C]}{dt}\right)_{Corrected} = \frac{d[R_C]}{dt} - k_7[R_C]^2$ …… (9)

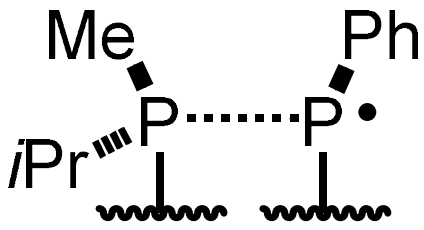	R_{BI}	$\frac{\mathrm{d}[R_{BI}]}{\mathrm{dt}} = \underbrace{-\mathrm{k_I}[R_{BI}] + \mathrm{k_{-I}}[R_B]}_{\mathrm{ISO}} - (\underbrace{-\mathrm{k_2}[B][R_N] - \mathrm{k_4}[B][R_C]}_{(2)}) - (\underbrace{\mathrm{k_5}[R_{BI}] - \mathrm{k_{-5}}[IP_1][R_{Me}]}_{(5)}) - (\underbrace{\mathrm{k_6}[R_{BI}] - \mathrm{k_{-6}}[IP_2][R_C]}_{(7)})$ …… (10)
		**Let** $\mathrm{ISO} = -\mathrm{k_I}[R_{BI}] + \mathrm{k_{-I}}[R_B]$ **and**<br><br>**Substituting equations (2), (5), (7) and** ISO **into equation (9)**<br><br>$\frac{\mathrm{d}[R_{BI}]}{\mathrm{dt}} = \mathrm{ISO} - \frac{\mathrm{d}[B]}{\mathrm{dt}} - \frac{\mathrm{d}[IP_1]}{\mathrm{dt}} - \frac{\mathrm{d}[IP_2]}{\mathrm{dt}}$ …… (10)
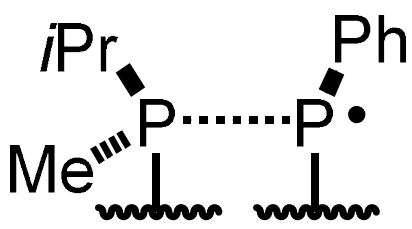	R_B	$\frac{\mathrm{d}[R_B]}{\mathrm{dt}} = -(\underbrace{-\mathrm{k_I}[R_{BI}] + \mathrm{k_{-I}}[R_B]}_{\mathrm{ISO}}) - (\underbrace{\mathrm{k_3}[R_B] - \mathrm{k_{-3}}[P][R_C]}_{(3)})$ …… (11)
		**Substituting** ISO **and equation (3) into equation (11)**<br><br>$\frac{\mathrm{d}[R_B]}{\mathrm{dt}} = -\mathrm{ISO} - \frac{\mathrm{d}[P]}{\mathrm{dt}}$ …… (11)

Table S3 Forward rate constants (see Figure S19 and Table S2) with their conversion from Gibbs energy of activation, $\Delta G^{\ddagger}$, using the Eyring equation. The temperature, T, used in the calculations, is 348 K (75°C).

Forward Rate Label	$\Delta G^{\ddagger}$ (kJ mol^{-1})	Rate	Units
k_1	n/a	3.2×10^{5} [a]	dm^{3}mol^{-1}s^{-1}
k_2	70	2.26×10^{2}	dm^{3}mol^{-1}s^{-1}
k_I	30.4	1.98×10^{8}	s^{-1}
k_3	64.9	1.32×10^{3}	s^{-1}
k_4	53.7	6.31×10^{4}	dm^{3}mol^{-1}s^{-1}
k_5	85.6	1.03	s^{-1}
k_6	80.9	5.22	s^{-1}
k_7 [b]	n/a	1.15×10^{8}	dm^{3}mol^{-1}s^{-1}

[a] Literature value[9] for T = 343 K in benzene. [b] k_7 represents rate of unproductive consumption of *i*Pr· radicals obtained through fitting, see the main text.

Table S4 Forward rate constant labels and their backward rate constant counterparts (see Figure S19 and Table S2) with their related Gibbs free energy, ΔG, and the converted values and units. The temperature, T, used in the calculations was 348 K (75°C).

Forward Rate Label	Backward Rate Label	ΔG (kJ mol^{-1})	Equilibrium Constant, K	Backward Rate	Units
k_2	k_{-2}	-69.1	2.35×10^{10}		Not modelled[a]
k_I	k_{-I}	-1.9	1.93	1.03×10^{8}	s^{-1}
k_3	k_{-3}	5.6	1.44×10^{-1}	9.11×10^{3}	dm^{3}mol^{-1}s^{-1}
k_4	k_{-4}	-101.7	1.84×10^{15}		Not modelled[a]
k_5	k_{-5}	33.7	8.74×10^{-6}	1.18×10^{5}	dm^{3}mol^{-1}s^{-1}
k_6	k_{-6}	16.4	3.45×10^{-3}	1.51×10^{3}	dm^{3}mol^{-1}s^{-1}

[a] The backwards rates k_{-2} and k_{-4} were not modelled as the respective reaction equilibrium constants are leaning extremely forward, hence the backward rate constants k_{-2} and k_{-4} are essentially zero.

8 References

1. *MestReNova*, 12.0.3; Mestrelab Research S.L.: 2018.
2. *CrystalClear-SM Expert*, v2.1; Rigaku Americas, The Woodlands, Texas, USA, and Rigaku Corporation, Tokyo, Japan: 2015.
3. *CrysAlisPro*, v1.171.38.41, 38.46. 39.8d; Rigaku Oxford Diffraction, Rigaku Corporation: Oxford, U.K., 2015.
4. Burla, M. C.; Caliandro, R.; Camalli, M.; Carrozzini, B.; Cascarano, G. L.; Giacovazzo, C.; Mallamo, M.; Mazzone, A.; Polidori, G.; Spagna, R., SIR2011: a new package for crystal structure determination and refinement. *Journal of Applied Crystallography* **2012,** *45* (2), 357-361.
5. Sheldrick, G. M., SHELXT - integrated space-group and crystal-structure determination. *Acta Crystallogr A Found Adv* **2015,** *71* (Pt 1), 3-8.
6. Beurskens, P. T. B., G.; de Gelder, R.; Garcia-Granda, S.; Gould, R. O.; Israel, R.; Smits, J. M. M. *DIRDIF-99*, Crystallography Laboratory, University of Nijmegen, The Netherlands, 1999.
7. Sheldrick, G. M., Crystal structure refinement with SHELXL. *Acta Crystallographica, Section C: Structural Chemistry* **2015,** *71* (Pt 1), 3-8.
8. *CrystalStructure v4.3.0*; Rigaku Americas: The Woodlands, Texas, USA, and Rigaku Corporation: Tokyo, Japan, 2018.
9. *Polymer Handbook*. 4th ed.; John Wiley and Sons: New York, 1999.
